# Ambient light alleviates retinal neurodegeneration in mice by powering mitochondria via the engineered optoenergetic rhodopsin

**DOI:** 10.1038/s41392-025-02450-1

**Published:** 2025-10-30

**Authors:** Run-Zhou Yang, Yiting Wang, Zhuanbin Wu, Yun Luo, Dian-Dian Wang, Yun Zou, Youzhi Liang, Jia-Kang Li, Su Zhang, Chun-Ping Huang, Wei-Rong Zeng, Si-Yuan Chang, Sen-Miao Li, Xiao-Yan Meng, Hui-Fang Sun, Pei-Pei Liu, Jinzhi Lei, Yang Xiang, Yu Gu, Biao Yan, Shi-Qing Cai, Jiayi Zhang, Jian-Sheng Kang

**Affiliations:** 1 Clinical Systems Biology Laboratories, The First Affiliated Hospital of Zhengzhou University, Zhengzhou, 450052 China; 2 State Key Laboratory of Brain Function and Disorders, MOE Frontiers Center for Brain Science, Institutes of Brain Science, Institute for Medical and Engineering Innovation, Eye & ENT Hospital, Fudan University; Shanghai Academy of Natural Sciences (SANS), Shanghai, 200032 China; 3 Shanghai Model Organisms Center, Inc., Shanghai, China; 4 Center for Excellence in Brain Science and Intelligence Technology, Chinese Academy of Sciences, Shanghai, 200031 China; 5 The Academy of Medical Sciences, Zhengzhou University, Zhengzhou, 450001 China; 6 National Clinical Research Center for Infectious Diseases, Guangdong Provincial Clinical Research Center for Tuberculosis, Shenzhen Third People’s Hospital, Southern University of Science and Technology, Shenzhen, 518112 China; 7 Metabolic Control and Aging, Human Aging Research Institute (HARI) and School of Life Science, Nanchang University, and Jiangxi Key Laboratory of Human Aging, Nanchang, 330031 China; 8 School of Mathematical Sciences, Center for Applied Mathematics, Tiangong University, Tianjin, 300387 China

**Keywords:** Molecular neuroscience, Experimental models of disease, Metabolic engineering, Metabolic disorders

## Abstract

The mitochondrial proton motive force (pmf) is a critical driver of cellular energy production and influences various cellular processes. Dysregulation of pmf is implicated in a range of diseases, including neurodegenerative diseases, mitochondrial diseases, cancer and aging-related pathologies. Currently, an efficient strategy to rescue ATP production and mitigate reactive oxygen species (ROS) generation under conditions of energy deprivation is lacking. Here, we engineered a light-sensitive, mitochondria-targeting proton-pumping rhodopsin (PPR), mt-EcGAPR, capable of generating an efficient pmf for ATP synthesis while simultaneously mitigating reactive oxygen species (ROS) generation during stress and decreasing DNA double-strand breaks (DSBs). Owing to its transparency to visible light, eye is the ideal candidate for the noninvasive application of mt-EcGAPR in the treatment of mitochondria-related retinal degenerative diseases. Using a silicone oil-induced ocular hypertension glaucoma mouse model, we demonstrate that ambient light activation of mt-EcGAPR significantly increased ATP production, suppressed ROS accumulation, and protected retinal ganglion cells (RGCs) from degeneration. Mechanistically, mt-EcGAPR inhibited endoplasmic reticulum (ER) stress-ATF6-gasdermin D (GSDMD)-mediated pyroptosis, thereby preserving retinal structure and function. This intervention ultimately led to improved visual acuity in glaucomatous eyes of mice. Collectively, our findings establish mt-EcGAPR as a promising therapeutic strategy for glaucoma and potentially other neurodegenerative diseases associated with mitochondrial dysfunction and impaired bioenergetics.

## Introduction

Mitochondria are eukaryotic endosymbiotic organelles that serve as central hubs for cellular energy production, thermogenesis, ion and metabolite homeostasis, and the regulation of cell survival. The mitochondrial proton motive force (pmf), which consists of a charge gradient (∆ψ_m_) and a chemical gradient (∆pH), plays a pivotal role in bioenergetics, calcium homeostasis and reactive oxygen species (ROS) production. The pmf is generated by the electron transport chain (ETC), which pumps protons from the mitochondrial matrix into the intermembrane space, creating an electrochemical gradient across the inner mitochondrial membrane. Maintaining an optimal pmf is critical for cellular health. A diminished pmf can impair ATP synthesis, whereas an excessively high pmf may lead to increased ROS production due to impaired electron transport within the ETC.^1^ Dysregulation of pmf has been implicated in a wide range of pathological conditions, including neurodegenerative diseases^2^ such as Parkinson’s and Alzheimer’s, metabolic disorders like diabetes,^3^ retinal degenerative diseases,^4^ cancer,^5^ and aging.^6^ Despite the central importance of pmf in health and disease, therapeutic strategies aimed at restoring pmf to a balanced and safe level remain limited. This is largely due to the complex interplay between pmf and various cellular processes, including energy metabolism, apoptotic signaling, ROS regulation, and mitochondrial dynamics.

Optogenetics has emerged as a powerful technique that utilizes light to modulate cellular activities with high spatiotemporal precision. Given the pivotal role of mitochondrial membrane potential (MMP) in cellular bioenergetics, targeting optogenetic tools to mitochondria presents a promising approach for manipulating pmf and potentially correcting mitochondrial dysfunction. Mitochondrion-targeted light-gated cations^7^ and anion channels^8^ have been demonstrated to potentially induce mitochondrial dysfunction. Light-driven proton-pumping rhodopsin (PPR), which is widely found in nature, ranging from archaebacteria to eukaryotic fungi,^9^ harnesses light energy to pump protons across cellular membranes, creating a proton motive force. In synthetic biology, PPRs have been successfully expressed in nonphotosynthetic microorganisms to enable light-driven ATP synthesis and the sustainable production of biochemicals.^10^ However, mitochondrial-targeted PPRs have not yet been successfully applied to mammalian models in vivo because the development of efficient delivery systems for these proteins into the crowded inner mitochondrial membrane remains a significant challenge. In particular, achieving this without compromising mitochondrial structure and functionality is difficult because of their large molecular size and inherent limitations in terms of mitochondrial membrane permeability. Furthermore, optogenetic proton pumps typically produce unidirectional hyperpolarization, which often leads to excessive ROS accumulation,^11^ undermining their therapeutic potential.

The inherent optical transmissivity of eyes provides a unique platform for novel therapeutic approaches, with optogenetics emerging as a noninvasive strategy with significant potential for treating retinal disorders. Among these conditions, glaucoma represents the leading cause of irreversible vision loss worldwide, primarily affecting retinal ganglion cells (RGCs). While elevated intraocular pressure (IOP) is widely recognized as a major risk factor, current IOP-lowering therapies frequently fail to adequately prevent disease progression and subsequent RGC degeneration. This has prompted researchers to explore alternative pathogenic mechanisms, among which mitochondrial dysfunction has emerged as a key contributor. Studies in both human patients and animal models have revealed mitochondrial abnormalities in glaucoma, including oxidative stress, ATP depletion, and deficiencies in mitochondrial complex I (MCI).^12–14^ These findings underscore the potential of targeting mitochondrial function as a novel therapeutic strategy for glaucoma. However, developing interventions that can effectively restore mitochondrial health and prevent RGC death remains a major challenge.

Here, we report the development of a novel mitochondrial-targeting PPR, termed mt-EcGAPR, designed to localize specifically to the inner mitochondrial membrane in mammalian cells. mt-EcGAPR achieves functional integration into the mitochondrial membrane without disrupting its overall function. Importantly, mt-EcGAPR operates near the physiological resting membrane potential, allowing for fine-tuned modulation of pmf while preserving ATP synthesis and maintaining ROS homeostasis. Particularly, the reversal potential of EcGAPR acts as a self-restricting factor for a beneficial pmf around −216 mV to prevent DNA double-strand breaks (DSBs) in cells expressing mt-EcGAPR under light exposure. This balanced modulation is a significant advancement over earlier optogenetic tools that tended to induce excessive hyperpolarization and oxidative stress.^11^ Functional validation of mt-EcGAPR was performed across multiple model organisms. In *Caenorhabditis elegans*, photostimulation of mt-EcGAPR effectively mitigated neuronal degeneration under conditions of mitochondrial stress. Similarly, in zebrafish larvae with pharmacologically induced MCI inhibition, mt-EcGAPR activation significantly reduced neuronal cell death, highlighting its neuroprotective potential. Most importantly, we tested mt-EcGAPR in a mouse model of ocular hypertension-induced glaucoma. In this model, activation of mt-EcGAPR led to a marked increase in retinal ATP levels, restoration of mitochondrial integrity, and a significant reduction in ROS accumulation. Mechanistically, we found that mt-EcGAPR activation attenuated endoplasmic reticulum (ER) stress and inhibited the ATF6-gasdermin D (GSDMD) signaling pathway. By mitigating this pathway, mt-EcGAPR appears to protect RGCs from pyroptosis. Remarkably, mt-EcGAPR was activated effectively even under ambient light conditions, eliminating the need for invasive light-delivery systems and enhancing its translational potential. Collectively, these results establish mt-EcGAPR as a promising therapeutic modality for glaucoma and mitochondrial related neurodegenerative disorders, such as diabetic retinopathy, through the optogenetic regulation of mitochondrial energetics.

## Results

### Molecular screening for mitochondrial-targeted PPRs

To target light-driven PPRs to mitochondria, we evaluated a variety of microbial rhodopsins. Given the evolutionary connection between mitochondria and α-proteobacteria,^15^ we hypothesized that α-proteobacterial PPRs might be effectively targeted to mitochondria. This led to the identification of twelve alpha proteorhodopsins from GenBank. Among these, alpha xanthorhodopsin (AXR) and alpha proteorhodopsin (APR) were synthesized with optimized human codon usage for screening. For a comprehensive assessment, we also included established PPRs such as bacteriorhodopsin (BR), Archaeorhodopsin T (ArchT),^16^ deltarhodopsin (HtdR),^17^ Gloeobacter rhodopsin (GR), green proteorhodopsin (GPR),^18^ Coccomyxa rhodopsin (CsR),^19^ and Leptosphaeria maculans (MAC)^20^ in our screening process. Phylogenetic analysis revealed that these microbial rhodopsins can be categorized into four families (Supplementary Fig. 1a).

**Fig. 1:**
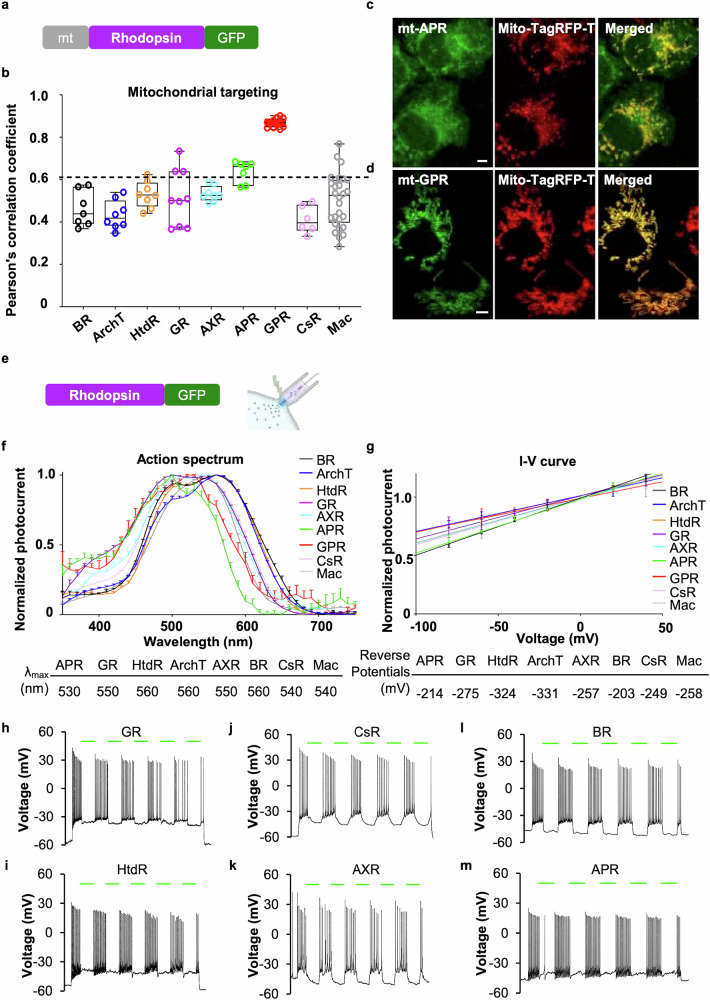
Screening of mitochondrion-targeted PPRs and their proton-pumping activities. **a** Mitochondrial-targeting strategy for microbial rhodopsins. The mitochondrial signal peptide (mt,4-cox8) and EGFP were fused to the N-terminus and C-terminus of microbial rhodopsins, respectively. **b** Mitochondrial targeting assay. The mitochondrial-targeted microbial rhodopsins were expressed in COS-7 cells. The colocalization of microbial rhodopsins with a mitochondrial marker (mito-tag RFP-T) was evaluated with Pearson’s correlation coefficient (Pearson’s *r*). The following microbial rhodopsins were investigated: BR (*n* = 7, black), ArchT (*n* = 8, blue), HtdR (*n* = 8, orange), GR (*n* = 9, purple), AXR (*n* = 8, cyan), APR (*n* = 7, green), GPR (*n* = 12, red), CsR (*n* = 6, pink), and MAC (*n* = 28, gray). The dashed horizontal line was used as a threshold for effective mitochondrial targeting. **c** Representative confocal images of mt-APR with the mitochondrial marker mito-TagRFP-T. Scale bar, 5 μm. **d** Representative confocal images of mt-GPR with the mitochondrial marker mito-TagRFP-T. Scale bar, 5 μm. **e** Schematic illustration of EGFP-fused PPRs expressed in HEK293T cells for patch clamp recordings. **f** Photocurrent spectra of the PPRs. Action spectra were measured via whole-cell patch–clamp imaging of HEK293T cells upon illumination with light (3.315 mW mm^−2^) through the visible spectrum at pH 7.4. The following PPRs were measured: BR (*n* = 3, black), ArchT (*n* = 10, blue), HtdR (*n* = 3, orange), GR (*n* = 4, purple), AXR (*n* = 5, cyan), APR (*n* = 4, green), CsR (*n* = 4, pink), and MAC (*n* = 4, gray). The data are presented as the mean ± SEM. The peak photocurrent for each PPR is listed below. **g** *I*–*V* curves of the PPRs. Normalized stationary photocurrents upon light stimulation (532 nm laser, 130 mW mm^−2^) were measured at pH 7.4 at holding potentials ranging from −80 mV to 40 mV with a 20 mV step. The following PPRs were measured: BR (*n* = 3, black), ArchT (*n* = 4, blue), HtdR (*n* = 3, orange), GR (*n* = 3, purple), AXR (*n* = 3, cyan), APR (*n* = 4, green), CsR (*n* = 7, pink), and MAC (*n* = 6, gray). All the photocurrents were normalized relative to the photocurrents at 0 mV. The data are presented as the means ± SEMs. The reverse potentials for each PPR determined from the linear regression analysis are listed below. **h**–**m** The proton-pumping activity of PPRs can silence neuronal activity. The following PRRs were investigated: GR (**h**), HtdR (**i**), CsR (**j**), AXR (**k**), BR (**l**), and APR (**m**). A series of green light pulses (532 nm, 130 mW mm^−2^, duration: 1 s, interval: 1 s) were delivered. The green lines indicate the light stimulation period. Time scale for the green line, 1 s. Action potentials were evoked through current injections

For mammalian mitochondria-targeting screening, a quadruply repeated mitochondrial signal peptide from cytochrome c oxidase subunit VIII (4-COX8, mt) was screened and selected. It was subsequently fused to the N-terminus of these PPR proteins, while enhanced green fluorescent protein (EGFP) was appended to their C-termini (Fig. 1a). These mt-fused PPRs were expressed in COS-7 cells, and their mitochondrial targeting ability was quantified by calculating Pearson’s correlation coefficients with mitochondrial markers (mito-TagRFP-T) (Fig. 1b). Pearson’s correlation coefficient indicated successful mitochondrial localization of GPR and APR, with coefficients of 0.87 ± 0.01 and 0.64 ± 0.02, respectively (Fig. 1c, d, and Supplementary Fig. 1h). In contrast, BR, ArchT, HtdR, GR, AXR, MAC, and CsR failed to target mitochondria effectively (Supplementary Fig. 1b–g). We also analyzed the colocalization of the ER, Golgi, plasma membrane, and mitochondrial proteins with mitochondrial markers (Supplementary Fig. 1h). The results revealed that proteins with coefficients less than 0.6 did not effectively target mitochondria. Previous studies have reported the mitochondrial targeting of BR,^21^ HtdR,^22^ and MAC^20^ in yeast, *Drosophila*, and *C. elegans*, respectively. However, these PPRs did not exhibit the same targeting capabilities in our assays using mammalian cells (Supplementary Fig. 1b, d, h).

The highly negative membrane potential of the mitochondrial inner membrane, approximately −180 mV,^23^ which is essential for ATP synthesis, generates an electrical field that counteracts the activity of outward proton pumps. It was necessary to investigate the functionality of these PPRs under such negative membrane potentials. We fused these PPRs with EGFP at the C-terminus to monitor the targeting and expression of PPRs-EGFP in HEK293T cells (Fig. 1e). Owing to the varying pumping activities of PPRs at different wavelengths, we utilized whole-cell patch–clamp recordings in HEK293T cells to characterize their action spectra. The results revealed that the peak photocurrent for the PPRs occurred between 530 and 560 nm (Fig. 1f), so further experiments were conducted using a green laser (532 nm). We subsequently recorded the *I*–*V* curves of these PPRs by measuring stationary photocurrents upon green light stimulation (532 nm) at different holding voltages in HEK293T cells (Fig. 1g). Notably, GPR did not produce detectable photocurrents in HEK293T cells, which is consistent with previous studies.^24^^,^^25^ All photocurrent-measurable PPRs showed reduced proton pump activity at more hyperpolarized membrane potentials, with reverse potentials estimated below −180 mV. These findings indicate that these PPRs maintain their proton-pumping ability under typical mitochondrial membrane conditions (Fig. 1g). The reliability of the reversal potentials for PPRs was validated by the high R-squared values for the *I*–*V* curve fitting, all of which were above 0.95 (Supplementary Table 1). Furthermore, photocurrent-measurable PPRs inhibited neuronal firing upon light stimulation (Fig. 1h–m), similar to the photoinhibition effect of ArchT reported previously.^16^

### Optimizing mitochondrial-targeted PPRs

**Fig. 2:**
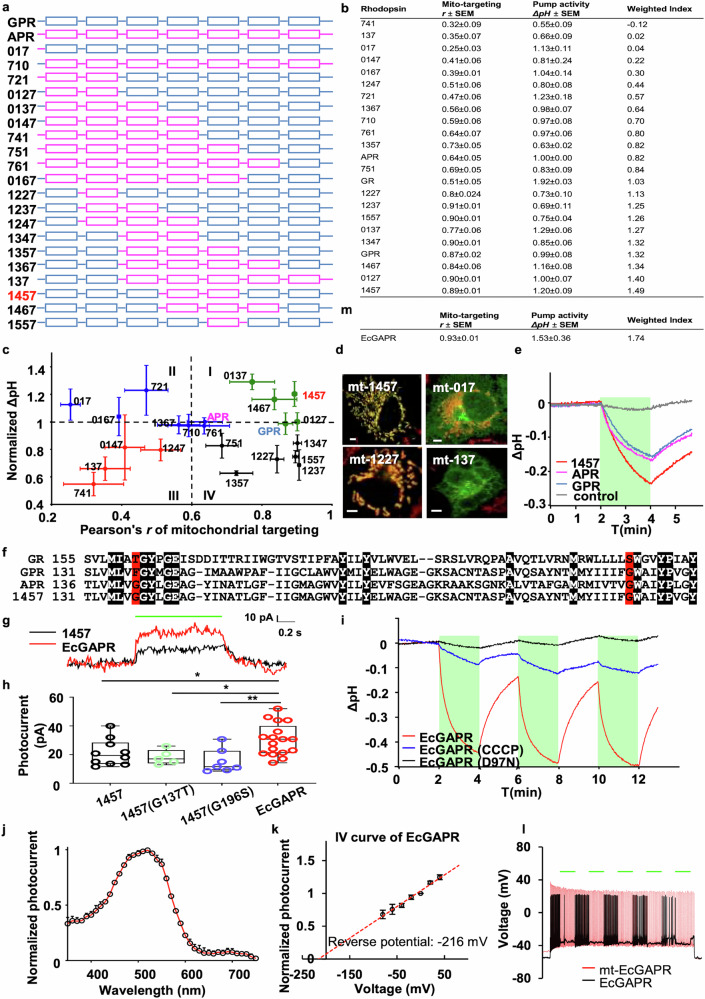
Constructing, characterizing, and optimizing APR-GPR chimeras. **a** Chimeric constructs of APR and GPR. Chimeras were generated by swapping homologous transmembrane segments of APR and GPR on the basis of sequence similarity. The transmembrane segments from GPR and APR are depicted in blue and magenta, respectively. The chimeras were named as follows: 710, 017, 721, 0127, 0137, 0147, 741, 751, 761, 0167, 1227, 1237, 1247, 1347, 1357, 1367, 137, 1457, 1467, and 1557. The chimera numbering system uses GPR as the backbone. The replaced GPR transmembrane (TM) helices are indicated by their helix numbers. For example, “1457” means that GPR TM4 and TM5 were replaced by APR TM4 and TM5, respectively. If the initial segment is from APR, the sequence is inverted (e.g., “751” signifies APR TM1–TM5 coupled with GPR TM6–TM7). **b** Pumping activities and mitochondrial targeting efficiencies of APR–GPR chimeras. The pumping activities were assessed by measuring the light-induced pH changes in *E. coli* suspensions, which were normalized to the values obtained for APR. The mitochondrial targeting efficiencies of the chimeric proteins were determined via Pearson’s correlation coefficient (*r*) with a mitochondrial marker. The table was sorted using a weighted index derived from the parameters r and ΔpH at a 2:1 ratio to evaluate the mitochondrial targeting efficiency and proton pumping functionality. This index serves as a key factor for identifying favorable chimeras. **c** Plot of all the chimeras with their pumping activities and mitochondrial targeting efficiencies. For quantification of pumping activity, the light-induced pH change of each chimera was normalized to the APR value. Pearson’s correlation coefficients between the mt-fused chimeric proteins and mitochondrial markers were utilized to determine the mitochondrial targeting efficiency. The data are presented as the means ± SEMs. The pH change assays of each chimera were conducted more than three times. Mitochondrial targeting was analyzed in more than four COS-7 cells. The chimeras could be grouped into four clusters according to the K-means cluster analysis of pumping activities and mitochondrial targeting efficiency (cluster I, green; cluster II, blue; cluster III, red; cluster IV, black). **d** Representative merged confocal images of the mt-fused chimeras (green channel) and TMRM (red channel) in each cluster (cluster I: 1457; cluster II: 017; cluster III: 1227; cluster IV: 137). Scale bars, 5 μm. **e** Representative traces of light-induced pH changes measured in *E. coli* suspensions upon illumination with a green laser (532 nm, 37.18 mW mm^−2^). Compared with the APR or GPR, Chimera 1457 presented greater pumping activity. The green bar indicates the light stimulation period. **f** Sequence alignment of GR, GPR, APR, and chimera 1457. The residues highlighted in red in chimera 1457 were mutated to the corresponding residues in GR. **g** Representative photocurrent traces of 1457 (black) and EcGAPR (red). Photocurrents were measured via whole-cell patch–clamp imaging of HEK293T cells upon illumination with a green laser (532 nm, 130 mW mm^−2^). The green bar indicates the period of light stimulation. Scale bar, horizontal, 0.2 s; vertical, 10 pA. **h** Quantification of outward stationary photocurrents of chimeric 1457 and its mutants. Compared with 1457 (*n* = 10) or single mutants (G137T, *n* = 5; G196S, *n* = 7), chimera 1457, with two mutations, G137T and G196S, named EcGAPR (*n* = 17), presented an increased photocurrent. *p < 0.05; **p < 0.01; *t* test. **i** Proton-pumping activity of EcGAPR. The pH of the *E. coli* suspensions was measured upon laser (532 nm, 37.18 mW mm^−2^) stimulation. EcGAPR showed a dramatic pH change, but the amplitude could be inhibited by CCCP (a pH gradient in which the protonophore collapses). EcGAPR(D97N), a mutant in which Asp97 is replaced by Asn, completely blocks pH changes, as Asp97 is involved in proton transport as a proton acceptor. **j** Photocurrent spectrum of EcGAPR measured via whole-cell patch–clamp in HEK293T cells. The photocurrent of EcGAPR (*n* = 5 cells) was measured upon illumination with light (3.315 mW mm^−2^) through the visible spectrum at pH 7.4. **k** *I*–*V* curve of EcGAPR. Stationary photocurrents of EcGAPR upon green laser stimulation (532 nm, 130 mW mm^−2^) were measured at pH 7.4 at holding potentials ranging from −80 mV to 40 mV with a 20 mV step. All the photocurrents are normalized to the photocurrents at 0 mV. The lines are linear fits to the data. The data are presented as the means ± SEMs (*n* = 6 cells). **l** Membrane potential recordings of mt-EcGAPR- or EcGAPR-expressing hippocampal neurons upon light stimulation. The green bars indicate the light stimulation period (532 nm, 37.18 mW mm^−2^, 1 s). Action potentials were invoked by current injection. Mt-EcGAPR (red) has no neuronal photoinhibitory effect compared with EcGAPR (black). **m** Mitochondrial targeting efficiency, proton pumping activity, and a weighted index of EcGAPRs. The results were aligned with the data presented in panel (**b**) for comparison

Proteorhodopsins, specifically GPR and APR, have potential for mitochondrial targeting (Fig. 1b–d). In human embryonic kidney 293 T cells, while GPR did not yield detectable photocurrents, APR demonstrated measurable photocurrent activity with a peak at 530 nm and a reversed potential of −213 mV (Fig. 1g, h). Notably, APR exhibited lower mitochondrial targeting efficiency than did GPR (Fig. 1c, d, and Supplementary Fig. 1g). These findings prompted the development of chimeric PPRs aimed at increasing both targeting accuracy and proton-pumping efficacy. To achieve this goal, we systematically constructed 20 chimeras by strategically swapping homologous segments between GPR and APR, leveraging their sequence similarity (Fig. 2a).

Owing to the challenges in measuring photocurrents for certain PPRs, such as GPR, in mammalian cells, we evaluated these chimeras in *E. coli* to assess their proton pumping efficacy. These chimeras were expressed in *E. coli* and subsequently resuspended in a buffer-free solution. The pumping activities were evaluated by monitoring light-induced pH changes (ΔpH) in an *E. coli* suspension via a pH microelectrode. To compare the pumping activities, these *E. coli* suspensions were adjusted to the same bacterial concentration (1 g/mL), illuminated with a green laser (532 nm, 37.18 mW cm^−2^) for 2 min, and this process was repeated 3 times with an interval of 2 min. The ΔpH values were compared with the peak of the first light stimulation and normalized to that of APR (Fig. 2b, c).

To evaluate the ability of each chimera to target mitochondria, a mitochondrial targeting signal (4cox8, mt) was appended to the N-terminal ends of the chimera, with EGFP fused to the C-terminal ends for monitoring purposes. These chimeras were expressed in COS-7 cells, and their mitochondrial targeting efficiencies were quantified via Pearson’s correlation coefficient (*r*), which was used to assess the correlation between the localization of the mt-fused chimeras and mitochondrial markers (Fig. 2b). These coefficients were also plotted and scaled along the horizontal axis of Fig. 2c. Consequently, the ΔpH values and correlation coefficients of the chimeras were subjected to K-means cluster analysis, resulting in four clusters, as indicated in Fig. 2c. The mitochondrial localization efficiencies of the chimeric samples across clusters are illustrated in Fig. 2d (cluster I: 1457, cluster II: 017, cluster III: 137, cluster IV: 1227), highlighting their representative targeting ability. The chimeras in cluster I demonstrated preferred mitochondrial targeting and pumping activity, whereas the chimeras in clusters II and III showed inadequate mitochondrial targeting (Fig. 2d). Compared with those in APR, the chimeras in clusters III and IV exhibited reduced proton pumping abilities (Fig. 2c).

A weighted index, incorporating parameters *r* and ΔpH at a 2:1 weight ratio, was used to evaluate the overall mitochondria-targeting efficiency and pump activity (Fig. 2b). Chimera 1457, with the fourth and fifth transmembrane segments of GPR replaced with those from APR, achieved the highest weighted index (Fig. 2b). Compared with those of GPR and APR, the pumping activity of chimera 1457 was greater (Fig. 2e), and the mitochondrial targeting efficiency of chimera 1457 was comparable to that of GPR (Fig. 2b, d).

Further evaluation revealed that the photocurrent of chimera 1457 in HEK293T cells was relatively low compared with that of other rhodopsins, such as GR. In contrast, GR caused a substantial pH change in *E. coli* (Fig. 2b). On the basis of the sequence similarity of the PPRs, we strategically introduced mutations at the retinal-binding residues of chimera 1457 to align them with those of GR (Fig. 2f). Through this mutagenesis optimization, we obtained a double mutant, 1457 (G137T, G196S), named EcGAPR (enhanced chimeric GPR and APR). This variant resulted in an increased photocurrent in HEK293T cells (Fig. 2g, h). The photocurrent of EcGAPR approached saturation at a light power of approximately 28 mW mm^−2^, exhibiting almost all-or-none responses to light stimulation (Supplementary Fig. 2a, b). To verify the proton pumping activity of EcGAPR, we added CCCP, a protonophore that disrupts the pH gradient across the membrane, to a suspension of *E. coli* expressing EcGAPR. Additionally, we mutated the Asp 97 residue in EcGAPR to Asn, which is analogous to Asp 85 in BR, a key residue involved in proton transfer. The results in Fig. 2i demonstrated that the light-induced ΔpH was significantly reduced by CCCP treatment, and the D97N mutation completely abolished the proton-pumping ability, suggesting that EcGAPR is a light-driven proton pump.

Patch–clamp recordings from HEK293T cells expressing EcGAPR revealed an action spectrum with a peak at 520 nm (Fig. 2j). The reversal potential of the photocurrent was approximately −216 mV (Fig. 2k), which is close to the physiological mitochondrial electrochemical gradient and typically ranges from −180 mV to −250 mV. Patch–clamp recordings in neurons expressing mt-EcGAPR-EGFP and EcGAPR-EGFP revealed the absence of detectable photocurrents on the plasma membrane, indicating the specific targeting of mt-EcGAPR-EGFP to mitochondria (Fig. 2l). The characteristics of all the screened PPRs are detailed in Supplementary Table 1. In summary, EcGAPR demonstrated optimal mitochondrial-targeting capability and pumping activity in PPR screening (Fig. 2m).

**Fig. 3:**
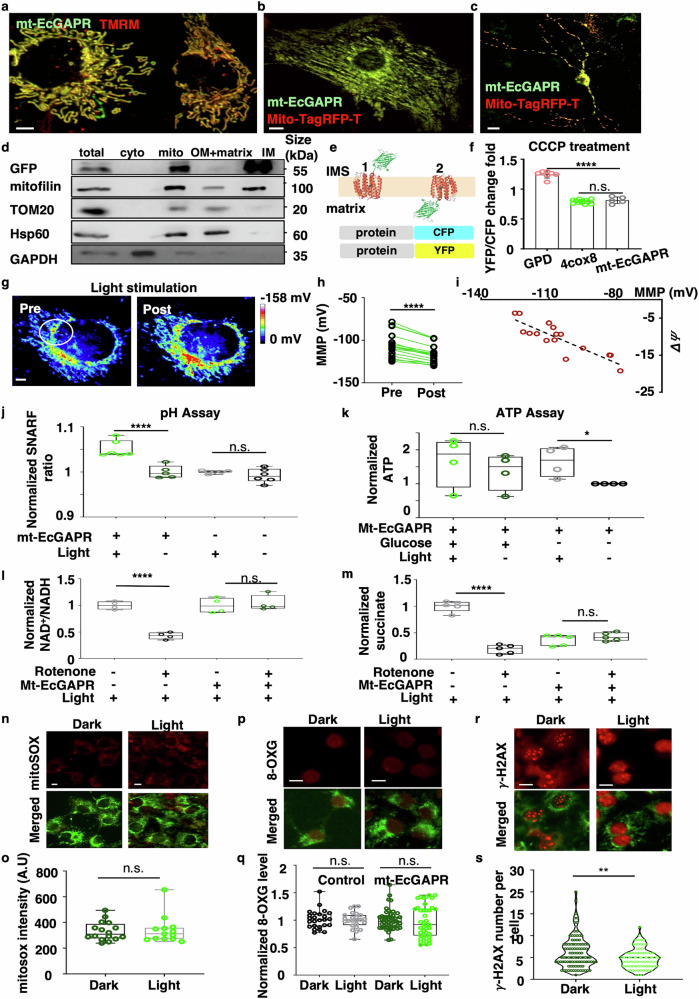
Optoenergetic effects of mitochondrial-targeted EcGAPR. **a** Confocal image of a COS7 cell line stably expressing mitochondrial-targeting signal-fused EcGAPR (mt-EcGAPR) and stained with TMRM, a mitochondrial voltage-sensitive marker. Scale bar, 5 μm. **b** Confocal image of primary cultured cardiomyocytes cotransfected with mt-EcGAPR-EGFP and Mito-TagRFP-T. Scale bar, 5 μm. **c** Confocal image of primary cultured hippocampal neurons cotransfected with mt-EcGAPR-EGFP and Mito-TagRFP-T. Scale bar, 5 μm. **d** Western blot analysis of the submitochondrial localization of mt-EcGAPR. HEK293T cells expressing mt-EcGAPR-EGFP were fractionated into distinct components: the cytosol, mitochondria, inner mitochondrial membrane, outer mitochondrial membrane, and matrix. The subcellular distribution of mt-EcGAPR-EGFP was compared with that of known markers, such as mitofilin (a mitochondrial inner membrane protein), Tom20 (a mitochondrial outer membrane protein), Hsp60 (a mitochondrial matrix protein), and GAPDH (a cytosolic protein). **e** Topology assay of mt-EcGAPR. mt-EcGAPR-EGFP exhibited two potential topologies when located in the inner mitochondrial membrane, with opposite pumping directions and localization of the C-terminal-fused fluorescent protein (state 1 and state 2). EYFP fluorescence is pH sensitive, whereas ECFP fluorescence is pH insensitive, allowing the EYFP/ECFP fluorescence ratio to serve as an indicator of pH changes. **f** YFP/CFP ratio change upon CCCP treatment in C-terminal EYFP/ECFP-fused mt-EcGAPR-, GPD-, and 4cox8-transfected cells. The CCCP, which collapses the pH gradient between the matrix and intermembrane space, acidifies the matrix and alkalizes the intermembrane space. pH changes were monitored following treatment with CCCP (25 μM) for C-terminal EYFP/ECFP-fused mt-EcGAPR, as well as two other proteins with known mitochondrial subspace locations: Glyceraldehyde-3-phosphate dehydrogenase (GPD), a mitochondrial intermembrane protein, and a fourfold repetitive signal peptide of cytochrome c oxidase 8 (4cox8), which specifically localizes to the mitochondrial matrix. The response of mt-EcGAPR-ECFP/EYFP to CCCP was significantly different from that of GPD-ECFP/EYFP, whereas it was similar to that of 4cox8-ECFP/EYFP (GPD, *n* = 7; 4cox8, *n* = 5; mt-EcGAPR, *n* = 11; GPD vs. mt-EcGAPR, *p* < 0.0001, ****; 4cox8 vs. mt-EcGAPR, *p* = 0.3082, n.s.). **g** Representative images of the mitochondrial membrane potential indicated by rhodamine 800 before and after light stimulation (515 nm, 320 s) in the mt-EcGAPR-stably transfected COS7 cell line. The mitochondrial membrane potential was calculated via the Nernst equation and is presented as a pseudocolor. The white circle indicates the region of light stimulation. Scale bar, 5 μm. **h** Quantification of the mitochondrial membrane potential before and after light stimulation (*n* = 16 cells, paired *t* test, *p* < 0.0001, ****). **i** Relationship between light-induced changes in the mitochondrial membrane potential (Δψ) and the initial MMP. **j** pH assay. Mitochondria were isolated from the mt-EcGAPR-EGFP COS7 cell line and stained with SNARF-1-AM. The SNARF-1 fluorescence ratio was measured before and after light stimulation (532 nm, 37.18 mW) for 10 min. **k** ATP assays. The mt-EcGAPR-EGFP COS7 cell line was cultured in glucose starvation medium (1 g/L) or high-glucose medium (4.5 g/L) in the dark or under light illumination (37.18 mW mm^−2^ green light). ATP significantly increased under light conditions under glucose starvation conditions (*p* = 0.03, one-tailed *t* test), whereas ATP did not differ under light conditions under high-glucose culture conditions (*p* = 0.5, *t* test). The data are presented as the means ± SEMs (*n* = 4 replicates). **l** NAD^+^/NADH assay. The mt-EcGAPR-EGFP-expressing HeLa cells were cultured in a CO_2_ incubator under light illumination (37.18 mW green light) with or without the addition of 1 μM rotenone. NAD^+^/NADH was significantly decreased (*p* = 2.4 × 10^−5^, *t* test) in rotenone-treated control cells but remained unchanged (*p* = 0.71, *t* test) in mt-EcGAPR-expressing cells. The data are presented as the means ± SEMs (*n* = 4 replicates). **m** Succinate assay. The mt-EcGAPR-expressing HeLa cells were cultured in a CO_2_ incubator under light illumination (37.18 mW of green light) with or without the addition of 1 μM rotenone. Succinate was significantly decreased (*p* < 0.0001, *t* test) in rotenone-treated control cells but remained unchanged in mt-EcGAPR cells (*p* = 0.325, *t* test). The data are presented as the means ± SEMs (*n* = 5 replicates). **n** Fluorescence images of mt-EcGAPR-EGFP stably transfected COS7 cells stained with mitoSOX before and after light stimulation. The cells supplemented with 10 μM all-trans retinal were illuminated with green light (532 nm, 900 ms, 1 Hz, 1000 lux) for 16 h in a culture chamber. After light stimulation, the cells were stained with mitoSOX, an indicator of ROS. Scale bars, 10 μm. **o** Quantification of mitoSOX fluorescence. There was no significant generation of ROS upon light stimulation (mt-EcGAPR under light, *n* = 42; mt-EcGPR in the dark, *n* = 41, *p* = 0.7796, *t* test). **p** Fluorescence images of mt-EcGAPR-EGFP stably transfected HeLa cells stained with 8-oxoguanine before and after light stimulation. The cells supplemented with 10 μM all-trans retinal were illuminated with green light (532 nm, 900 ms, 1 Hz, 1000 lux) for 16 h in a culture chamber. After light stimulation, the cells were stained with 8-oxoguanine, an indicator of DNA damage caused by ROS. **q** Quantification of 8-oxoguanine fluorescence. There was no significant DNA damage upon light stimulation (mt-EcGAPR under dark, *n* = 51; mt-EcGAPR under light, *n* = 42 cells; control cells under dark, *n* = 24; control cells under light, *n* = 25 cells; mt-EcGAPR dark vs. mt-EcGAPR light, *p* = 0.5985; control dark vs. control light, *p* = 0.7414, *t* test). **r** Fluorescence images of mt-EcGAPR-EGFP stably transfected HeLa cells stained with γ-H2AX before and after light stimulation. The cells supplemented with 10 μM all-trans retinal were illuminated with green light (37.18 mW) for 16 h in a culture chamber. After light stimulation, the cells were stained with an anti-γ-H2AX antibody, a marker of DNA double-strand breaks (DSBs). Scale bar, 10 μm. **s** Violin plot of γ-H2AX particles. The numbers of cells used for γ-H2AX analysis were as follows: mt-EcGAPR dark, *n* = 98; mt-EcGAPR light, *n* = 107 (*p* = 0.0017, *t* test)

As anticipated, mt-EcGAPR-EGFP exhibited robust mitochondrial localization in COS7 cells, primary cultured CMs, and neurons (Fig. 3a–c). Western blotting analysis of submitochondrial fractions from HEK293T cells expressing mt-EcGAPR-EGFP revealed a similar localization pattern to that of mitofilin, a canonical mitochondrial inner membrane protein (Fig. 3d), indicating that mt-EcGAPR-EGFP was exclusively targeted to the mitochondrial inner membrane. To determine the orientation of mt-EcGAPR-EGFP on the inner mitochondrial membrane, we explored the pH sensitivity of the fluorescent proteins (Fig. 3e). The C-terminal EGFP of mt-EcGAPR-EGFP could be positioned either in the intermembrane space (state 1) or the matrix (state 2). We utilized the YFP/CFP ratio to monitor pH changes, as YFP is more pH sensitive than CFP is. Matrix-targeted (4cox8-EYFP/ECFP) and intermembrane space-targeted (GPD-EYFP/ECFP) fluorescent proteins, along with the test protein (mt-EcGAPR-EYFP/ECFP), were expressed in COS-7 cells. The protonophore CCCP, which collapses the mitochondrial membrane potential and alters the pH gradient, acidifies the matrix while alkalinizing the intermembrane space. This controlled pH manipulation enables the determination of protein topology on the basis of changes in fluorescence ratios. The results (Fig. 3f) revealed that the C-terminus of mt-EcGAPR-EGFP faces the matrix, indicating that EcGAPR is correctly oriented within the inner membrane. Overall, on the basis of the screening results, EcGAPR was engineered as the optimal PPR with increased pumping activity and efficient mitochondrial targeting.

### Optoenergetic effects of mitochondrial EcGAPR

As a proton pump, mt-EcGAPR generates a proton gradient across the mitochondrial inner membrane, contributing to pmf. To investigate the optoenergetic effects of mt-EcGAPR, we established a COS-7 cell line stably expressing mt-EcGAPR-EGFP. We confirmed the proton-pumping activity of EcGAPR within mitochondria by loading mt-EcGAPR cells with the mitochondrial membrane potential (MMP) indicator dye rhodamine 800. The Δψ, which was calculated via the Nernst equation, is represented with pseudocolor in Fig. 3g. Light stimulation was applied to a specified region (white circle in Fig. 3g) via a 515 nm confocal laser. Following light stimulation, a significant increase in the Δψ was observed (Fig. 3h, *p* < 0.0001, paired *t* test), suggesting that EcGAPR retains its proton-pumping ability in mitochondria. Notably, the change in Δψ was inversely proportional to the initial Δψ (Fig. 3i), which was in line with the voltage-dependent I-V curve of EcGAPR activity (Fig. 2k). In contrast, the Δψ of cells lacking mt-EcGAPR did not change upon light stimulation (Supplementary Fig. 2c, d). Mitochondria were subsequently isolated from the mt-EcGAPR cell line and stained with the pH-sensitive dye SNARF-1-AM. The pH changes in isolated mitochondria before and after light stimulation were compared. The results indicated that light led to a significant increase in the pH of the matrix (Fig. 3j; light/dark: 1.05 ± 0.075, *P* < 0.0001, *t* test). Thus, both Δψ and ΔpH, which collectively form the pmf, significantly increased in response to light stimulation. As pmf is crucial for ATP synthesis via complex V of the ETC, the ATP level upon light illumination was measured in COS-7 cells stably expressing mt-EcGAPR. Interestingly, light stimulation only slightly increased ATP production under high-glucose conditions (4.5 g/L) (Fig. 3k). However, under glucose depletion, light stimulation of mt-EcGAPR-expressing COS7 cells led to a significant increase in ATP production (Fig. 3k, *P* < 0.05), whereas no effect was observed in cells lacking mt-EcGAPR (Supplementary Fig. 2e, *P* > 0.05). In the absence of sufficient glucose, the capacity to generate ATP through glycolysis and subsequent mitochondrial oxidative phosphorylation is reduced, leading to decreased mitochondrial membrane potential and a diminished proton gradient. The reduced MMP under glucose depletion enhanced the pumping activity of EcGAPR (Fig. 3k). These findings highlight the optoenergetic interplay of the mitochondrial EcGAPR, which couples light to cellular energy metabolism.

Furthermore, we observed that rotenone, a mitochondrial complex I inhibitor, caused a decrease in NADH consumption and a compensatory increase in succinate consumption (Fig. 3l, m; fold change: succinate, rotenone/control = 0.18, *P* < 0.0001, *t* test; NAD^+^/NADH, rotenone/control = 0.43, *P* < 0.0001, *t* test). Strikingly, light stimulation effectively reversed these alterations in the NAD^+^/NADH ratios and succinate levels in HeLa cells expressing mt-EcGAPR (Fig. 3l, m; fold change: succinate, rotenone/control = 1.1475, *P* = 0.3250, *t* test; NAD^+^/NADH, rotenone/control = 1.039, *P* = 0.7145, *t* test). These findings suggest that mt-EcGAPR, when stimulated by light, can generate sufficient pmf to sustain mitochondrial respiration and maintain the balance of NAD^+^/NADH. Collectively, our findings demonstrated that mt-EcGAPR is an optoenergetic tool capable of enhancing pmf under light stimulation, particularly under stress conditions such as glucose depletion and complex I inhibition.

### Self-restriction property of mt-EcGAPR

A previous study revealed that under light, the *E. coli* strain expressing GR produced 81% more ATP and had 82% higher levels of ROS than did the control group.^11^ Since an increase in the mitochondrial membrane potential (MMP) can lead to excessive ROS production,^26^ it was crucial to determine whether the mitochondrion-targeted EcGAPR would produce ROS when the cells were exposed to light. ROS levels were measured via the use of mitoSOX (Fig. 3n, o: light/dark = 1.028, *P* = 0.7796), a sensor for mitochondrial ROS, and dihydroethidium (DHE) (Supplementary Fig. 2f: DHE: light/dark = 1.002, *P* = 0.74), a sensor for cytosolic ROS. These findings indicated that light stimulation did not significantly increase ROS production in cells expressing mt-EcGAPR. Immunostaining for 8-oxoguanine, a marker of ROS-induced DNA damage,^27^ also revealed no significant increase in DNA oxidative damage due to light stimulation (Fig. 3p, q; light/dark = 0.9737, *P* = 0.5985). To further investigate DNA double-strand breaks (DSBs) that might result from oxidative damage, we performed immunostaining for γ-H2AX, a marker of DSBs and DNA repair.^28^ Interestingly, cells expressing mt-EcGAPR under light stimulation for 16 h presented a slight reduction in DSBs of approximately 24% (Fig. 3r, s; light/dark = 0.77, *P* = 0.0025, *t* test).

Intriguingly, EcGAPR is a voltage-dependent proton pump with a reversal potential of −216 mV (Fig. 2k). This reversal potential closely aligns with the physiological mitochondrial electrochemical gradient (−180 mV to −250 mV), suggesting that EcGAPR exhibits relatively low or reversed pumping activity under resting conditions. This inherent property likely contributes to its self-restriction behavior within mitochondria. Under depolarized conditions (low MMP), the proton pumping activity of EcGAPR is increased, enabling cells to generate adequate pmf for ATP production. Conversely, under normal or hyperpolarized conditions (high MMP), the activity of EcGAPR is reduced or reversed, preventing excessive ROS production and DSB damage (Fig. 3n, s). In contrast, the reversal potential of GR is −275 mV (Fig. 1h), which is more hyperpolarized than the resting membrane potential of *E. coli*.^29^ This difference might explain why GR caused ROS generation under light in *E. coli*. Our results suggested that the reversal potential of EcGAPR could be a key self-restricting factor for a safer pmf of approximately −216 mV to prevent ROS generation and DSBs in cells expressing mt-EcGAPR under light exposure, indicating that mt-EcGAPR is a safe optoenergetic tool for in vivo applications.

### Neuroprotection of mt-EcGAPR in a *C. elegans* model

We first tested the in vivo functionality of mt-EcGAPR in rescuing mitochondrial defects induced by rotenone in *C. elegans*. The four cephalic neurons (CEPs), identifiable by their extended dendrites in the head region (Supplementary Fig. 3a, b), serve as indicators for monitoring dopaminergic neuron degeneration associated with Parkinson’s disease (PD).^30^ Rotenone, a mitochondrial complex I inhibitor, can induce apoptosis by increasing mitochondrial ROS generation. Chronic exposure to rotenone is known to result in neuronal degeneration and symptoms reminiscent of PD in *C. elegans*.^31^ The dopamine transporter promoter (*dat-1*) is used to express mt-EcGAPR specifically in dopaminergic neurons. The mitochondrial targeting of mt-EcGAPR was confirmed by the colocalization of mt-EcGAPR and TOMM20 (Supplementary Fig. 3c–e). Light stimulation was performed with a green LED (~540 nm, 10,000 lux) in an incubator maintained at 20 °C for 5 days (Supplementary Fig. 3f). Light stimulation had protective effects on CEP neurons only in those *C. elegans* with mt-EcGAPR expression and retinal supplementation all-trans-retinal (ATR) (Supplementary Fig. 3g, h; +ATR, +light vs −ATR, −light, *P* < 0.0001; +ATR, +light vs −ATR, +light, *P* < 0.0001; +ATR, +light vs +ATR, −light, *P* < 0.0001). Worms expressing mt-EcGAPR without retinal supplementation presented with CEP neuron loss upon light exposure, ruling out the possibility of a protective effect from light itself. The movement paths before and after light exposure, recorded over a span of 2 min, are depicted in Supplementary Fig. 3i, j. The speeds of *C. elegans* expressing mt-EcGAPR with rotenone treatment significantly increased after light exposure (Supplementary Fig. 3i–k; locomotion speed, mm/s, mean ± SEM: light: 1.41 ± 0.19, dark: 0.79 ± 0.07, *P* = 0.0147), suggesting that light exposure might preserve CEP neurons and improve motor deficits in worms expressing mt-EcGAPR with retinal supplementation. We also coinjected *dat1::4cox8::GFP* and *dat1::mcherry* as a control (Supplementary Fig. 4a–c), which revealed that light stimulation had no protective effect on *C. elegans* with 4cox8-GFP expression in the presence of ATR supplementation (Supplementary Fig. 4d; +ATR, +light vs −ATR, −light, *P* > 0.05; +ATR, +light vs −ATR, +light, *P* > 0.05; +ATR, +light vs +ATR, −light, *P* > 0.05).

Taken together, these results demonstrated that photoactivation of mt-EcGAPR effectively mitigated neurodegeneration and restored normal movement in a *C. elegans* model with mitochondrial complex I and pmf deficiencies induced by rotenone treatment. These findings highlight the therapeutic potential of mt-EcGAPR in addressing mitochondrial dysfunction-associated disorders.

### Photoprotective effects of mt-EcGAPR against cell death in zebrafish

Paraquat (PQ, 1′-dimethyl-4,4′-bipyridinium dichloride), which acts as a mitochondrial toxicant by disrupting MCI, leads to increased production of reactive oxygen species (ROS) and causes cell death in zebrafish.^32^ We investigated the protective function of mt-EcGAPR under light stimulation via PQ-induced zebrafish models to assess cell death. Zebrafish embryos (AB strain) were injected with either the pcDNA3-mt-EcGAPR plasmid or a nonpumping mutant counterpart, pcDNA3-mt-EcGAPR (D97N). At 2 days post-fertilization (dpf), the embryos were exposed to PQ and subjected to green LED light (~540 nm, 10,000 lux with 900 ms ON and 100 ms OFF cycles) for 24 h in a controlled incubator set at 28.5 °C (Supplementary Fig. 5a, b). A significant increase in the number of apoptotic hair cells was detected in the PQ-treated group compared with the control group (Supplementary Fig. 5c, d, g; cell number, mean ± SEM: vehicle, 0.1 ± 0.03; PQ, 5.2 ± 0.067; *P* < 0.0001). Notably, the number of apoptotic hair cells was substantially lower in embryos injected with mt-EcGAPR and treated with light than in those injected with the mt-EcGAPR(D97N) mutant (Supplementary Fig. 5e–g; mean ± SEM: PQ + D97N = 4.2 ± 0.13, PQ + mt-EcGAPR = 0.6 ± 0.101, *P* < 0.0001). These findings suggest that mt-EcGAPR has a photoprotective effect on PQ-induced apoptosis in zebrafish. For the analysis of macrophage migration, we utilized the TG (*zlyz:EGFP*) transgenic line. Embryos were injected with either pcDNA3-mt-EcGAPR or its D97N mutant at the one-cell stage (Supplementary Fig. 5h). At 2 dpf, the plants were exposed to PQ and subjected to green LED light for 48 h under the same conditions (Supplementary Fig. 5h). The fluorescence images were analyzed after conversion to an 8-bit grayscale (Supplementary Fig. 5o–q). PQ treatment significantly increased macrophage migration (Supplementary Fig. 5i, j), which was effectively mitigated in photostimulated embryos expressing mt-EcGAPR (Supplementary Fig. 5k–n, and r; mean cell count ± SEM: vehicle = 2.4 ± 0.0889, PQ = 101.9 ± 2.77, *P* < 0.0001; PQ + D97N = 98.4 ± 2.273, PQ + mt-EcGAPR = 27.6 ± 1.474, *P* < 0.0001). The D97N mutant served as a robust negative control, facilitating rigorous validation of the energetic effects mediated by mt-EcGAPR.

Collectively, our findings demonstrate that mt-EcGAPR under light stimulation provides robust protection against PQ-induced cellular damage in zebrafish models by reducing cell death and attenuating inflammatory responses. Given that PQ is significantly more toxic than rotenone is, these results highlight the versatile protective capacities of mt-EcGAPR in mitigating oxidative stress and mitochondrial dysfunction.

### Light activation of mt-EcGAPR rescues ATP deficits and ROS accumulation in ocular hypertension model mice

The use of light stimulation as a therapeutic intervention strategy is highly advantageous for treating retinal degenerative diseases, as light can easily pass through the cornea, pupil, and lens and reach the retina. Previous studies have demonstrated that glaucomatous RGCs exhibit impaired mitochondrial function,^33^ including decreased MMP,^34^ decreased ATP production^35^ and increased ROS levels,^36^ likely leading to RGC death. Thus, we explored the possibility of RGC protection by mt-EcGAPR in an ocular hypertension mouse model with glaucoma symptoms.

**Fig. 4:**
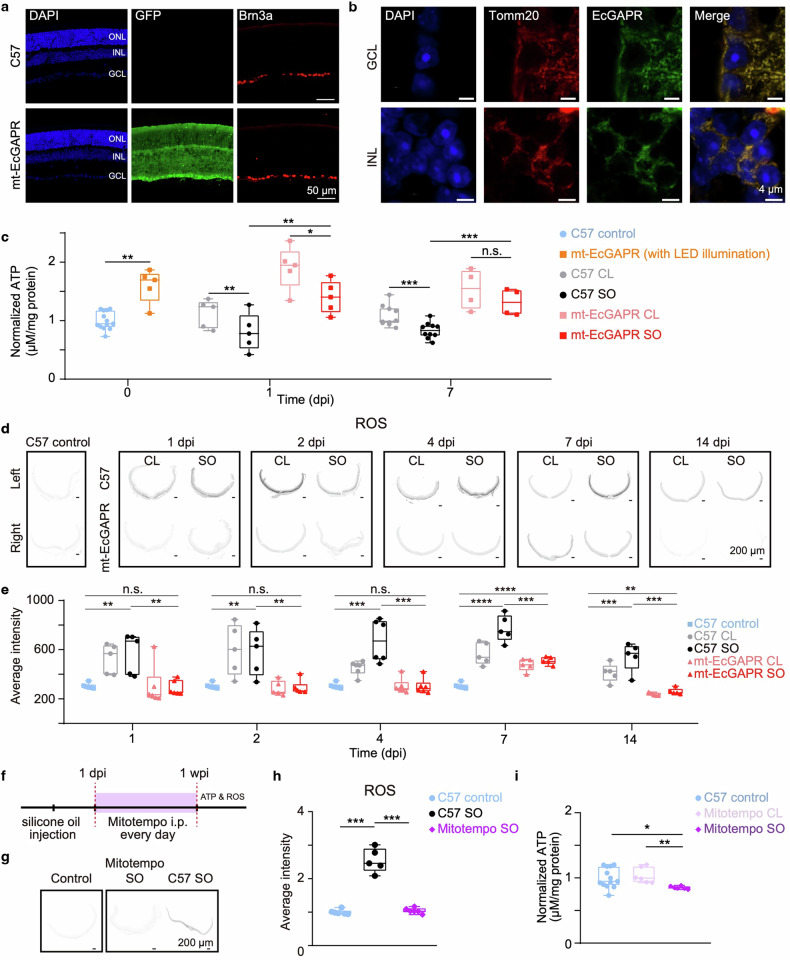
Light activation of mt-EcGAPR rescues ATP deficits and ROS accumulation in ocular hypertension model mice. **a** Representative retina vertical sections of C57BL/6 J mice (first row) and mt-EcGAPR-2A-EGFP mice (second row). Blue: DAPI, a nuclear counterstain. Green: GFP representing the expression of mt-EcGAPR. Red: Brn3a, a marker for retinal ganglion cells (RGCs). Scale bar, 50 μm. **b** Colocalization of mitochondria and mt-EcGAPR in the retinal ganglion cell layer (GCL) and inner nuclear layer (INL) of mt-EcGAPR mice. Blue: DAPI. Red: Tomm20, a marker of mitochondria. Green: mt-EcGAPR immunostained with myc antibody. Scale bar, 4 μm. **c** Retinal ATP levels in C57BL/6 J mice and mt-EcGAPR mice before and after silicone oil (SO) injection. Blue circles, nonglaucomatous control C57BL/6 J mice (*n* = 13). Orange rectangles represent nonglaucomatous control mt-EcGAPR mice with 4-h LED illumination (*n* = 5). Gray circles, Contralateral (CL) eyes of C57BL/6 J mice. Black circles, silicone-oil-injected (SO) eyes of C57BL/6 J mice. Pink rectangles, CL eyes of mt-EcGAPR mice. Red rectangles, SO eyes of mt-EcGAPR mice. *N* = 5 biological replicates for each strain at 1 dpi. *N* = 10 (C57BL/6 J mice) and *n* = 4 (mt-EcGAPR mice) at 7 dpi. Light stimulation significantly increased retinal ATP levels in mt-EcGAPR mice. **d** Representative images of retinal ROS levels in C57BL/6 J mice and mt-EcGAPR mice stained with dihydroethidium (DHE) before SO injection and at 1, 2, 4, 7, and 14 days after SO injection. Scale bar, 200 μm. **e** Retinal ROS levels in C57BL/6 J mice and mt-EcGAPR mice after SO injection. Blue rectangles, control C57BL/6 J mice (*n* = 6). The gray circles represent the CL eyes of C57BL/6 J mice. Black circles, SO eyes of C57BL/6 J mice. Pink tangles and CL eyes of mt-EcGAPR mice. Red triangles, SO eyes of mt-EcGAPR mice. *N* = 5, 5, 6, 5, and 5 for C57BL/6 mice and *n* = 6, 5, 6, 5, and 5 for mt-EcGAPR mice at 1 dpi, 2 dpi, 4 dpi, 7 dpi, and 14 dpi, respectively. **f** Timeline of mito-TEMPO experiments. Mito-TEMPO was administered daily via intraperitoneal injection following SO injection. **g** Representative images of retinal ROS in C57BL/6 J mice with or without Mito-TEMPO 1 week after SO injection and in nonglaucomatous control C57BL/6 J mice. Scale bar, 200 μm. **h** Retinal ROS levels in the eyes of C57BL/6 J mice with or without mito-TEMPO one week after SO injection and in nonglaucomatous control C57BL/6 J mice. Blue circles, control C57BL/6 J mice (*n* = 6). Black circles, SO eyes of C57BL/6 mice (*n* = 5). The SO eyes of C57BL/6 J mice with mito-TEMPO are purple diamonds (*n* = 6). **i** Retinal ATP levels of SO eyes and CL eyes of C57BL/6 mice with mito-TEMPO one week after SO injection and nonglaucomatous control C57BL/6 J mice. Blue circles, control C57BL/6 J mice (*n* = 13). Light purple diamonds, CL eyes of C57BL/6 J mice with mito-TEMPO (*n* = 6). Purple diamonds, SO eyes of C57BL/6 J mice with mito-TEMPO (*n* = 6). The data are presented as the means ± SEMs. Statistical results: *p < 0.05; **p < 0.01; ***p < 0.001; ****p < 0.0001; *t* test

We developed a knock-in mouse line with ubiquitous expression of mt-EcGAPR. This line was engineered to express mt-EcGAPR with a myc tag appended to the N-terminus of EcGAPR and 2A-EGFP fused to the C-terminus of EcGAPR (Supplementary Fig. 6a). These knock-in mice had lived for more than one year without significant weight changes (Supplementary Fig. 6b). Compared with that in C57BL/6 J mice, the expression of mt-EcGAPR in the retina of knock-in mice was confirmed by immunohistochemistry, with no apparent alterations in retinal structure (Fig. 4a). Confocal imaging further confirmed the colocalization of mt-EcGAPR with mitochondria (Fig. 4b). A silicone oil (SO)-induced ocular hypertension model^37^ was employed in this study. Stable, elevated intraocular pressure (IOP) was induced for up to 9 weeks after SO injection (Supplementary Fig. 6c–e). Retinal ATP levels were significantly lower in both C57BL/6 J and mt-EcGAPR mouse silicone oil-injected (SO) eyes than in contralateral (CL) eyes (Fig. 4c). All the mice were subjected to 530 nm green LED illumination (7200 lux) 4 h per day for 9 weeks following SO injection. LED illumination significantly increased retinal ATP levels in mt-EcGAPR mice without ocular hypertension, as well as in the SO eyes of mt-EcGAPR mice (Fig. 4c).

The retinal ROS levels were measured via DHE staining. In the SO eyes of C57BL/6 J mice, significant ROS accumulation was observed, particularly at 4–7 days following SO injection (Fig. 4d). Interestingly, a transient increase in retinal ROS levels was also observed in the CL eyes of C57BL/6 J mice during the first two days post-SO injection (Fig. 4e). In contrast, mt-EcGAPR mice presented significantly lower retinal ROS levels within 14 days after SO injection, with only a slight increase in the first week (Fig. 4d, e). To compare the effects of the optoenergetic activation of mt-EcGAPR with those of a traditional antioxidant approach, we administered mito-TEMPO, a mitochondrion-targeted ROS scavenger, intraperitoneally daily at a dose of 0.7 mg/kg body weight following SO injection (Fig. 4f). While Mito-TEMPO effectively reduced retinal ROS levels (Fig. 4g, h), it had no significant effect on retinal ATP levels (Fig. 4i). These results demonstrate that the optoenergetic activation of mt-EcGAPR effectively enhances ATP levels while mitigating oxidative stress in the glaucomatous retina, suggesting a potential therapeutic advantage over traditional antioxidant therapies.

### Transcriptomic and proteomic data indicate the protective role of mt-EcGAPR in mitigating ER stress and pyroptosis in ocular hypertension mice

**Fig. 5:**
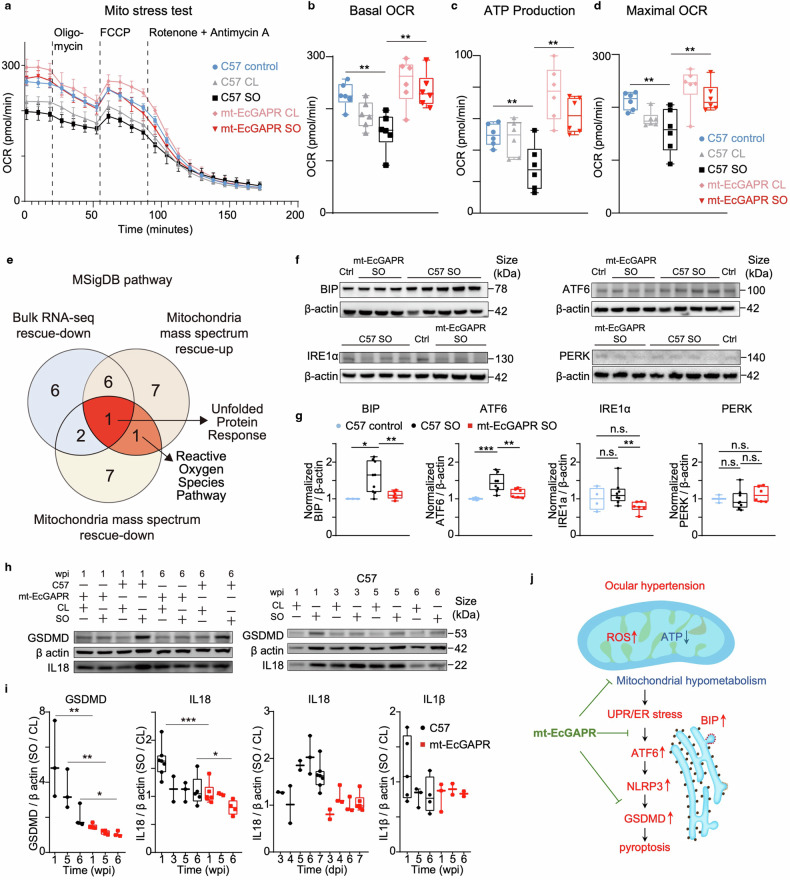
Light activation of mt-EcGAPR rescues mitochondrial dysfunction, alleviates ER stress, and inhibits pyroptosis in ocular hypertension model mice. **a** Oxygen consumption rate (OCR) curves of retinal patches of SO eyes and CL eyes from C57BL/6 J mice and mt-EcGAPR mice one day after SO injection and nonglaucomatous control C57BL/6 J mice. Blue circles, control C57BL/6 J mice. Gray tangles, CL eyes of C57BL/6 J mice. Black rectangles, SO eyes of C57BL/6 Jmice. Pink diamonds, CL eyes of mt-EcGAPR mice. Red diamonds, SO eyes of mt-EcGAPR mice. **b**–**d** Basal OCR (**b**), ATP production (**c**), and maximal OCR (**d**) of C57BL/6 J mice and mt-EcGAPR mice one day after SO injection and nonglaucomatous control C57BL/6 J mice. Blue circles, nonglaucomatous control C57BL/6 J mice (*n* = 6). Gray tangles, CL eyes of C57BL/6 J mice (*n* = 6). Black rectangles, SO eyes of C57BL/6 J mice (*n* = 6). Pink diamonds, CL eyes of mt-EcGAPR mice (*n* = 6). Red diamonds, SO eyes of mt-EcGAPR mice (*n* = 6). **e** Venn diagram of the pathways associated with the rescue-down genes, rescue-up proteins, and rescue-down proteins. **f** Representative Western blots for the ER stress markers BIP and ATF6 and a loading control (β-actin). **g** Quantification of relative protein expression of BIP, ATF6, IRE1α, and PERK. Blue circles, nonglaucomatous control C57BL/6 J mice. Black circles, SO eyes of C57BL/6 J mice. Red rectangles, SO eyes of mt-EcGAPR mice. **h** Representative Western blots of GSDMD and IL18 with β-actin as the loading control. **i** Quantification of GSDMD, IL-18, and IL-1β protein relative expression. Black circles, C57BL/6J mice. Red rectangles, mt-EcGAPR mice. **j** Summary of the protective mechanisms of the optoenergetic activation of mt-EcGAPR in ocular hypertension mice. Fonts highlighted in red represent upregulated factors in ocular hypertension mice; blue represents downregulation; and green represents rescue. The data are presented as the means ± SEMs. Statistical results: *p < 0.05; **p < 0.01; ***p < 0.001; *t* test

To gain deeper insight into the underlying mechanisms of the protective effects of mt-EcGAPR, we conducted comprehensive analyses of mitochondrial function and performed both bulk RNA-seq and mitochondrial protein mass spectrometry. The Seahorse mito-stress test revealed significant increases in the basal and maximal oxygen consumption rates (OCRs), as well as ATP production, in the retinae of mt-EcGAPR mice compared with those of C57BL/6 J mice 1 day after SO injection (Fig. 5a–d), indicating improved mitochondrial function in mt-EcGAPR mice.

Bulk RNA-seq of the retina revealed 1725 upregulated and 1266 downregulated genes in the SO eyes of C57BL/6 J mice compared with those of nonglaucomatous controls (Supplementary Fig. 7a). In contrast, comparison of SO eyes from LED-illuminated mt-EcGAPR mice with SO eyes from nonglaucomatous controls revealed 303 upregulated genes and 276 downregulated genes (Supplementary Fig. 7b). Notably, 125 genes upregulated in the SO eyes of C57BL/6 J mice were downregulated in mt-EcGAPR mice (rescue-up genes), and 81 genes downregulated in the SO eyes of C57BL/6 J mice were upregulated in mt-EcGAPR mice (rescue-down genes) (Supplementary Fig. 7c). Similarly, mitochondrial proteomics revealed 113 rescue proteins that were upregulated in the eyes of SO C57BL/6 J mice but downregulated in mt-EcGAPR mice, as well as 40 rescue proteins that were downregulated in C57BL/6 J mice but upregulated in mt-EcGAPR mice (Supplementary Fig. 7d).

RNA-seq and proteomics revealed that the unfolded protein response (UPR) was the only shared enriched pathway (Fig. 5e, and Supplementary Fig. 7e–g). The UPR is closely related to endoplasmic reticulum (ER) stress,^38^ and studies have shown that mitochondrial dysfunction and ER stress are strongly correlated.^39,40^ Additionally, MSigDB enrichment analysis revealed that pathways associated with calcium transport, protein metabolism, lipid metabolism, and cholesterol metabolism were enriched in both the bulk RNA-seq and mitochondrial protein mass spectrometry data (Supplementary Fig. 7). Previous studies have highlighted the critical role of mitochondria-associated endoplasmic reticulum membranes (MAMs) in the synthesis and transport of calcium ions, proteins, and lipids,^41–43^ which is consistent with the enrichment results.

BIP (also known as GRP78), a key molecular chaperone and central regulator of the UPR, was significantly upregulated in the SO eyes of C57BL/6 J mice but remained unchanged in mt-EcGAPR mice (Fig. 5f). These findings suggested that ER stress was activated in the SO eyes of C57BL/6 J mice but was mitigated in mt-EcGAPR mice. We also measured the expression of ATF6, IRE1α, and PERK, which are key components of three distinct ER stress-mediated pathways. Our results revealed that, compared with that in nonglaucomatous controls, ATF6 expression was significantly upregulated in the SO eyes of C57BL/6 J mice, whereas no significant change was detected in the SO eyes of mt-EcGAPR mice (Fig. 5f, g). These findings indicate that the optoenergetic activation of mt-EcGAPR might alleviate ER stress by suppressing the ATF6-mediated pathway.

In addition to the UPR, the ROS pathway was enriched with rescue-up and rescue-down proteins (Fig. 5e). Previous studies have emphasized the crucial roles of ER stress,^44^ mitochondrial dysfunction,^45^ and ROS accumulation^46^ in driving pyroptosis. Furthermore, recent studies have demonstrated that pyroptosis significantly contributes to RGC death in glaucoma.^47,48^ To investigate the potential role of pyroptosis in our model, we examined the expression of key pyroptosis-related genes, including GSDMD, IL-18, and IL-1β. One week after SO injection, we observed a significant increase in the expression of these genes in the SO eyes of C57BL/6 J mice, followed by a return to baseline levels in subsequent weeks. In contrast, the expression of these markers remained stable in mt-EcGAPR mice (Fig. 5h, i). Overall, our findings demonstrated that the optoenergetic activation of mt-EcGAPR rescued mitochondrial hypometabolism, alleviated the ER stress–ATF6 axis, and inhibited pyroptosis, leading to enhanced neuroprotective effects against glaucoma (Fig. 5j).

### Optoenergetic activation of mt-EcGAPR preserves retinal ganglion cells and vision in ocular hypertension model mice

**Fig. 6:**
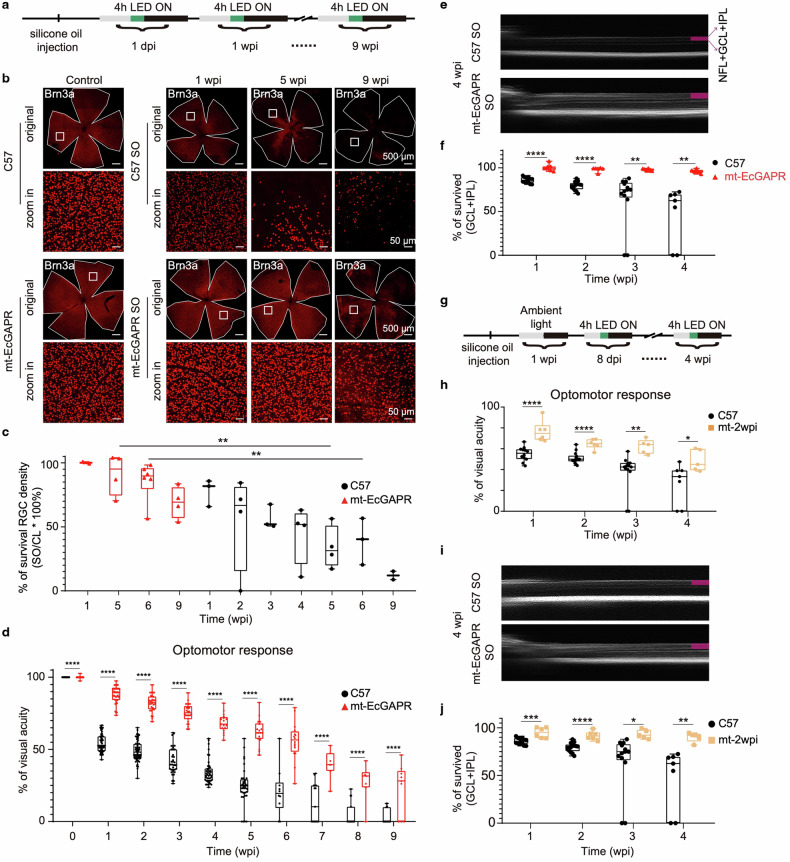
Optoenergetic activation of mt-EcGAPR partially rescues RGCs and improves vision in ocularly hypertensive mice. **a** Timeline of the LED light intervention experiments. Daily 4-h LED stimulation was administered to the mice following SO injection. **b** Representative immunohistochemistry staining images of whole-mount retinae from C57BL/6 J and mt-EcGAPR mice. Row 1: Representative images of C57BL/6 J mice before, 1 week, 5 weeks, and 9 weeks after SO injection. Row 3: Representative images of mt-EcGAPR mice before, 1 week, 5 weeks, and 9 weeks after SO injection. Scale bar: 500 μm. Rows 2 and 4: Zoomed-in images of the areas indicated by white rectangles in Rows 1 and 3, respectively. Scale bar, 50 μm. **c** Relative survival RGC density of C57BL/6 J mice and mt-EcGAPR mice after SO injection. Black circles, C57BL/6 J mice. Red triangles, mt-EcGAPR mice. *N* = 3, 4, 3, 4, 4, 3, and 2 for the C57BL/6 J mice and *n* = 2, 4, 6, and 4 for the mt-EcGAPR mice. **d** Visual acuity measured by the optomotor response of C57BL/6 J mice and mt-EcGAPR mice before and after SO injection. Black circles, C57BL/6 J mice. Red triangles, mt-EcGAPR mice. *N* = 54, 58, 35, 44, 38, 10, 15, 15, and 15 for C57BL/6 J mice and *n* = 40, 30, 26, 17, 18, 16, 7, 7, and 9 for mt-EcGAPR mice. **e** Representative optical coherence tomography (OCT) images of the SO eyes of C57BL/6 J mice and mt-EcGAPR mice 4 weeks after SO injection. Magenta rectangles indicate the inner retina, which includes nerve fiber layer (NFL), ganglion cell layer (GCL) and inner plexiform layer (IPL). Because it is hard to seperate NFL, inner retina is presented as (GCL + IPL) in the following results. **f** Relative survival inner retina thickness (GCL + IPL) of C57BL/6 mice and mt-EcGAPR mice after SO injection. Black circles, C57BL/6 mice. Red triangles, mt-EcGAPR mice. *N* = 12, 14, 13, and 7 for C57BL/6 J mice and *n* = 7, 6, 6, and 6 for mt-EcGAPR mice. **g** Timeline of the delayed LED light intervention experiments. Four-hour LED stimulation was initiated 2 weeks post-SO injection (wpi). **h** Visual acuity was measured by the optomotor response of C57BL/6 J mice and mt-EcGAPR mice subjected to LED stimulation 2 weeks post-SO injection. Black circles, C57BL/6 J mice. Orange rectangles, mt-EcGAPR mice. *n* = 12, 14, 13, and 7 for C57BL/6 J mice, and *n* = 6, 6, 5, and 5 for mt-EcGAPR mice. **i** Representative OCT images of the SO eyes of C57BL/6 mice and mt-EcGAPR mice. LED stimulation was initiated 2 weeks after SO injection, and images were acquired at 4 weeks post-SO injection. Magenta rectangles indicate the inner retina. **j** Relative survival inner retina thickness (GCL + IPL) of C57BL/6 J mice and mt-EcGAPR mice subjected to LED stimulation 2 weeks post-SO injection. Black circles, C57BL/6 J mice. Orange tangles, mt-2wpi mice. *N* = 12, 14, 13, and 7 for C57BL/6 J mice and *n* = 6, 6, 5, and 5 for mt-EcGAPR mice. The data are presented as the means ± SEMs. Statistical results: *p < 0.05; **p < 0.01; ***p < 0.001; ****p < 0.0001; *t* test

Given that gradual RGC loss is a hallmark of glaucoma,^49^ we assessed RGC survival via immunohistochemistry. Compared with C57BL/6 J mice, mt-EcGAPR mice presented higher RGC survival rates at 1, 5, and 9 weeks following SO injection (100%, 91%, and 69% in mt-EcGAPR mice vs. 78%, 34%, and 12% in C57BL/6 J mice at 1, 5, and 9 weeks, respectively; Fig. 6a–c). We also measured visual acuity via the optomotor response. Compared with C57BL/6 J mice, mt-EcGAPR mice maintained significantly better visual acuity throughout the 9-week observation period (88%, 62%, and 22% in mt-EcGAPR mice vs. 54%, 25%, and 3% in C57BL/6 J mice at 1, 5, and 9 weeks, respectively; Fig. 6d).

Previous studies have indicated that the inner retina, which includes the nerve fiber layer (NFL), ganglion cell layer (GCL), and inner plexiform layer (IPL), thins down as glaucoma progresses.^50^ To assess this effect, we measured the relative thickness of the inner retina before and after SO injection via optical coherence tomography (OCT). Compared with that of C57BL/6 J mice, the inner retina of mt-EcGAPR mice was thicker (Fig. 6e, f). Collectively, our findings demonstrated that the optoenergetic activation of mt-EcGAPR not only increased the survival of retinal ganglion cells (RGCs) but also preserved visual function in an experimental glaucoma model. Furthermore, clinical data indicate that many patients are first diagnosed with glaucoma at a late stage.^50^ We examined whether delayed optoenergetic activation of mt-EcGAPR might still protect RGCs and visual function by starting LED illumination one week after SO injection. Compared with C57BL/6 J mice, mt-EcGAPR mice presented significantly greater visual acuity and thicker inner retinae (Fig. 6g–j).

To evaluate the translational potential of optoenergetic activation, we administered AAV-EF1α-mt-EcGAPR via intravitreal injection. Three weeks after AAV virus injection, we injected SO and subsequently applied LED illumination for 4 h per day for 6 weeks (Supplementary Fig. 8a). Compared with that of C57BL/6 J mice and AAV-EGFP-injected mice, visual acuity was partially restored in AAV-EF1α-mt-EcGAPR-injected mice. Immunohistochemistry staining revealed that mt-EcGAPR was widely expressed after intravitreal injection, with an average transduction efficiency of approximately 36%. We also conducted dose-escalation experiments using two additional doses, 3.32 E8 and 3.32 E9 viral genomes/eye. These results indicated that neither dosage was toxic to RGCs. In addition, the expression of mt-EcGAPR could last for at least 9 weeks after intravitreal injection. Repeated injection may be necessary for long-term expression of mt-EcGAPR, which is a common procedure in anti-VEGF injections for clinical treatment. Together, these data support the potential therapeutic applicability of intravitreal delivery (Supplementary Fig. 8b–h).

### Ambient light can activate mt-EcGAPR and partially rescue RGCs and vision in ocular hypertension mice

**Fig. 7:**
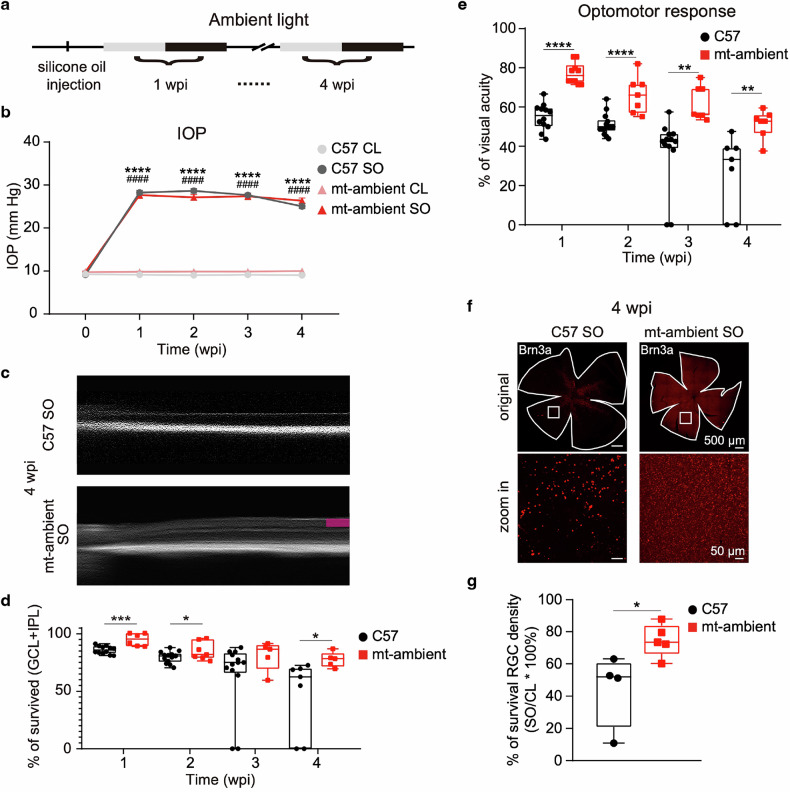
Ambient light activates mt-EcGAPR and partially rescues RGCs and vision in ocular hypertension model mice. **a** Timeline of the ambient light exposure experiments. The mice were exposed to ambient light following SO injection. The ambient light refers to the lighting used in the housing for the mice without additional green light supplementation, which is ~100 lux. **b** IOP curves of C57BL/6 J mice and mt-EcGAPR mice before and after SO injection exposed to ambient light. Light gray circles, CL eyes of C57BL/6 J mice. The medium gray circles represent the SO eyes of C57BL/6 J mice. Pink tangles and CL eyes of mt-EcGAPR mice. Red triangles, SO eyes of mt-EcGAPR mice. *N* = 15, 15, 15, and 15 for C57BL/6 J mice and *n* = 12, 8, 8, and 8 for mt-EcGAPR mice. **c** Representative optical coherence tomography (OCT) images of SO eyes in C57BL/6 J mice and mt-EcGAPR mice 4 weeks post-SO injection exposed to ambient light. Magenta rectangles indicate the inner retina. The magenta rectangle is absent in the OCT image of SO eye in C57BL/6 mice because the inner retina is almost degenerated. **d** Relative thickness of the inner retina (GCL + IPL) in C57BL/6 J mice (black circles) and mt-EcGAPR mice (red triangles) after SO injection and exposure to ambient light. Red triangles, mt-EcGAPR mice. *N* = 12, 14, 13, and 7 for C57BL/6 J mice and *n* = 6, 7, 5, and 5 for mt-EcGAPR mice. **e** Visual acuity measured by the optomotor response in C57BL/6 J mice and mt-EcGAPR mice after SO injection and exposure to ambient light. Black circles, C57BL/6 J mice. Red triangles, mt-EcGAPR mice. *N* = 12, 14, 13, and 7 for C57BL/6 J mice and *n* = 10, 7, 7, and 7 for mt-EcGAPR mice. **f** Representative immunohistochemistry staining images of whole-mount retinae. Row 1: Example images of the SO eyes of C57BL/6 J and mt-EcGAPR mice 4 weeks after SO injection. Scale bar, 500 μm. Row 2: Zoomed-in images of the areas indicated by white rectangles in Row 1; scale bar: 50 μm. **g** Relative survival RGC density of C57BL/6 J mice and mt-EcGAPR mice 4 weeks after SO injection exposed to ambient light. Black circles, C57BL/6 J mice (*n* = 4). Red rectangles, mt-EcGAPR mice (*n* = 5). The data are presented as the means ± SEMs. Statistical results: *p < 0.05; **p < 0.01; ***p < 0.001; ****p < 0.0001; *t* test

Intriguingly, 1 week after SO injection, mt-EcGAPR mice presented significantly better visual acuity and thicker inner retinae than C57BL/6 J mice did, even in the absence of additional LED illumination (Fig. 6h–j). Considering the broad excitation spectrum and light sensitivity of EcGAPR (Fig. 2j and Supplementary Fig. 2b), we maintained mt-EcGAPR and C57BL/6 J mice under standard indoor lighting conditions (~100 lux) following SO injection (Fig. 7a). Compared with C57BL/6 J mice, mt-EcGAPR mice presented significantly thicker inner retinae at 4 weeks (Fig. 7b–d). The optomotor response also revealed that more vision was preserved in mt-EcGAPR mice (Fig. 7e). Significantly increased RGC survival was also observed in mt-EcGAPR mice (Fig. 7f, g). Taken together, these results demonstrated that ambient light was sufficient to activate mt-EcGAPR and achieve protective effects.

Studies have shown that aging is associated with increased accumulation of ROS and deficits in ATP production, which may contribute to vision loss.^51^ We measured the visual function of C57BL/6 J and mt-EcGAPR mice aged 17–30 months (Supplementary Fig. 9a). Compared with age-matched C57BL/6 J mice, mt-EcGAPR mice presented greater visual acuity at 100% contrast (Supplementary Fig. 9b). Additionally, there was slightly greater contrast sensitivity in mt-EcGAPR mice than in C57BL/6 J mice at the same age (Supplementary Fig. 9c, d).

**Fig. 8:**
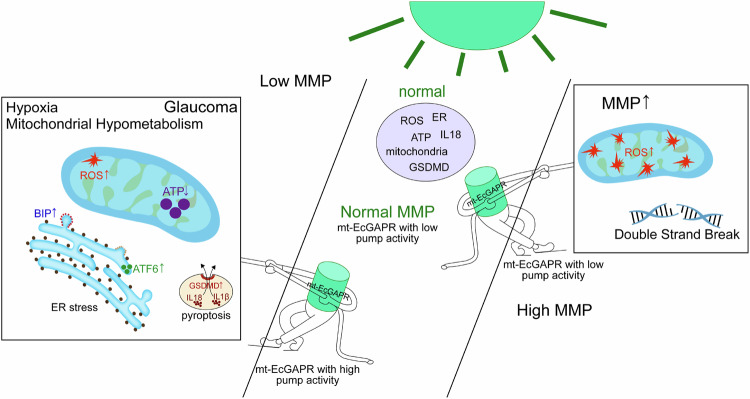
Summary of the protective mechanisms of the light activation of mt-EcGAPR. In glaucomatous pathogenesis, mitochondrial membrane potential (MMP) decline triggers excessive reactive oxygen species (ROS), ATP depletion, endoplasmic reticulum stress, and pyroptotic cell death. Light-activated mt-EcGAPR reverses these impairments by restoring MMP while reducing DNA double-strand breaks (DSBs). Under both low or high MMP conditions, its self-restriction property and ambient light (~100 lux) sensitivity ensure optimal mitochondrial proton motive force (pmf) around −216 mV to prevent excessive ROS and DSBs. This adaptive regulation preserves energy metabolism efficiency while sustaining balanced cellular stress responses and immune homeostasis, even in pathological MMP environments with compromised nutrient or oxygen availability

In summary, we have engineered mt-EcGAPR, a novel light-sensitive mitochondrial proton pump exhibiting a unique “tug-of-war” property. Under light, mt-EcGAPR displays high activity under low mitochondrial membrane potential (MMP) and low activity under high MMP, dynamically maintaining a safe pmf of ~216 mV for beneficial ATP and ROS homeostasis. Our findings demonstrate that in low-MMP conditions, such as glaucoma, the optoenergetic activation of mt-EcGAPR effectively rescues mitochondrial dysfunction by increasing ATP production, inhibiting ROS accumulation, and suppressing ER stress-ATF6-GSDMD axis-mediated pyroptosis (Fig. 8). Our study provides a convenient strategy for maintaining mitochondrial homeostasis and preventing cellular dysfunction in disease states.

## Discussion

Early studies have demonstrated that the acid-basic transition of isolated chloroplast mixtures can drive ATP synthesis, highlighting the potential of pH gradients as an energy-generating mechanism.^52^ Furthermore, incorporating the isolated purple membrane from *Halobacterium halobium* (which contains the light-driven proton pump bacteriorhodopsin) into lipid vesicles with mitochondrial ATPase has been shown to enable photophosphorylation.^53^ In the context of mammalian therapies, genetically transferring proton-pumping rhodopsin is a promising approach for treating diseases associated with mitochondrial dysfunction, particularly those involving deficiencies in mitochondrial respiratory complexes, such as complex I.^54,55^ However, microbial proton-pumping rhodopsins pose significant challenges because of their highly hydrophobic nature and the difficulty in effectively targeting them to mitochondria. To obtain the optimal optoenergetic proton pump, we applied four stringent criteria. First, the mitochondrion-targeted PPR must preserve normal mitochondrial morphology and function. Second, it should exhibit robust proton-pumping activity. Third, its mitochondrial targeting should be efficient in many different species and cell types, and it should not cause a leaky membrane current. Finally, the PPR should have a suitable reversal potential to prevent potential damage, such as depolarization or ROS generation. Guided by these criteria, we engineered mt-EcGAPR, a proton pump that meets the requirements for mitochondrial targeting and function. Our screening revealed that only rhodopsins from proteobacteria, which share an evolutionary origin with mitochondria, could be effectively targeted to mammalian mitochondria. This finding suggests that mammalian mitochondria retain the ability to accommodate proteins encoded by ancestral genes that were either lost or relocated to the nucleus during evolution.^56^ Chimeric studies have further demonstrated that swapping transmembrane segments can alter the targeting pattern of chimeric rhodopsins, underscoring the critical role of interactions between transmembrane helices in achieving optimal mammalian mitochondrial targeting and functionality.

The self-restricting property of the EcGAPR is attributed to its voltage dependence (Fig. 2k). Under nutrient-rich conditions, the hyperpolarized mitochondrial inner membrane potential restricts the pump activity of mt-EcGAPR. However, in response to specific stressors, such as exposure to inhibitors, including rotenone and paraquat, or nutrient depletion, the mitochondrial membrane potential becomes less polarized, enabling EcGAPR to exhibit elevated pumping activity (Fig. 8). This environmentally responsive behavior of mt-EcGAPR in mammalian cells is consistent with previous findings that heterogeneously expressed proteorhodopsin in *E. coli* provides a growth advantage under anaerobic conditions where energy availability is restricted.^57^ Furthermore, while Gloeobacter rhodopsin (GR) is known to generate ROS upon light activation,^11^ EcGAPR results in a less hyperpolarized potential (−216 mV) than does GR (−275 mV). In addition, mathematical models estimated that the resting pmf (*pmf*_*0*_) of COS-7 cells was −222 mV, with a 95% confidence interval for *pmf*_*0*_ being between −222 mV and −257 mV (see details in the Supplementary Text). The underestimation of *pmf*_*0*_ is likely attributed to the established understanding of a 0.4–0.6 pH unit gradient between mitochondrial cristae and the matrix, whereas more precise measurements reveal an actual transmembrane pH difference (ΔpH) spanning approximately 0.6–0.9 units, necessitating revised calculations for *pmf*_*0*_ estimation.^58^ Similarly, mt-EcGAPR under photostimulation reduced DNA oxidative damage compared with that in the control without photostimulation (Fig. 3r, s). Therefore, the optimized reversal potential of EcGAPR offers a significant advantage by preventing ROS production and subsequent oxidative DNA damage under normal physiological conditions (Figs. 3n–s and 4). We also conducted control experiments using permeability transition pore (PTP) inhibitors and measured the intracellular calcium levels. There was no significant change in the calcium level before and after light stimulation. Furthermore, the use of a negative control, mt-EcGAPR (D97N), a point mutation of mt-EcGAPR that disrupts the function of the proton pump, resulted in no protective effect in zebrafish (Supplementary Fig. 5). These data help exclude the potential involvement of endogenous stabilizers and suggest that the observed protective effects are specific to the proton pump of mt-EcGAPR. In addition, the proton-pumping activity of mt-EcGAPR modulated mitochondrial dynamics without disrupting permeability or calcium homeostasis.

Owing to their high energy demands, photoreceptors are particularly vulnerable to energy deficits. Mitochondrial hypometabolism has been implicated in several retinal disorders, including retinitis pigmentosa (RP), age-related macular degeneration (AMD), and diabetic retinopathy.^59,60^ Specifically, AMD patients exhibit abnormal mitochondrial structure,^61^ mtDNA mutations, and mitochondrial dysfunction^62^ in the RPE. Similarly, animal models of diabetic retinopathy exhibit alterations in mitochondrial inner membrane components, increased mitochondrial membrane permeability, and reduced ETC activity.^63^ Optogenetic therapies have shown promise in clinical trials for treating retinal degenerative diseases, particularly RP. For example, Sahel et al. demonstrated partial vision restoration in an RP patient through intravitreal gene delivery of an optogenetic protein to RGCs coupled with light stimulation from specialized goggles.^64^ Similarly, Bionic Sight LLC’s BS01 optogenetic therapy, which is currently in a phase 1/2 dose escalation study (NCT04278131), has reported partial restoration of light and motion detection in RP patients. Several other clinical trials (NCT02556736, NCT04919473, NCT04945772, and NCT05417126) have also reported visual improvements following treatment, underscoring the potential and feasibility of applying optogenetic therapies to retinal degenerative diseases. In contrast, glaucoma, despite being the leading cause of irreversible blindness worldwide^65^ and despite decades of research efforts, remains challenging to manage clinically. Current treatments focus primarily on intraocular pressure (IOP) reduction, which often yields unsatisfactory effects, such as ongoing degeneration of RGCs. Prosseda et al. achieved IOP reduction in a glaucoma mouse model by enhancing aqueous humor outflow via optogenetic activation of CRY2-OCRL, a CIBN/CRY2-based optogenetic construct.^66,67^ Additionally, Geeraerts et al. demonstrated reduced RGC degeneration through optogenetic modulation of neuronal activity in the superior colliculus (SC) of mice.^68^ However, these approaches face significant barriers for clinical translation owing to their invasiveness, requiring open-skull surgery, and reliance on high-intensity light stimulation. Interestingly, nonoptogenetic photobiomodulation therapies have shown promise in certain retinal conditions. For example, LumiThera’s Valeda light delivery system, approved for dry AMD treatment by the FDA, utilizes multiwavelength treatment (4 mW/cm^2^ at 590 nm, 65 mW/cm^2^ at 660 nm and 0.6 mW/cm^2^ at 850 nm) to preserve visual acuity and retinal anatomy in dry AMD patients (NCT04065490). Despite these advancements, a critical limitation of current therapeutic approaches is their inability to effectively halt the continuous degeneration and pyroptosis of RGCs.

In this study, the activation of mt-EcGAPR rescued ATP deficits, reduced ROS accumulation, and reversed mitochondrial hypometabolism in an ocular hypertension mouse model. Notably, ambient light (~100 lux) was sufficient to activate mt-EcGAPR, achieving protective effects on RGCs and vision, demonstrating its significant potential as a therapeutic intervention. In contrast to blue light stimulation, which has been reported to induce retinal pigment epithelium (RPE) and photoreceptor damage,^69,70^ mt-EcGAPR exhibits an activation spectrum peaking at 520 nm (Fig. 2j and Supplementary Table 1) with a low activation threshold (~100 lux ambient light, in comparison to clear-sky outdoor (>10,000 lux) lighting conditions). In particular, the human eye exhibits peak sensitivity to green light within the wavelength range of 500–565 nm^71,72^ which overlaps with the peak photon flux of natural light in the blue-green region (450–550 nm) as defined by the ASTM G-173 standard for natural daylight spectra. This spectral overlap optimizes human visual acuity under natural daylight conditions and explains the evolutionarily optimized spectral profile of PPRs, as illustrated in Fig. 1F. This spectral profile significantly mitigates potential phototoxicity concerns, enhancing its clinical applicability. Moreover, the pumping activity of EcGAPR is dependent on light intensity (Supplementary Fig. 2b), with the photocurrent reaching a plateau at approximately 30 mW/mm^2^, equivalent to approximately 20,490 lux with a wavelength of 555 nm for the human eye.^73^ Consequently, mt-EcGAPR is expected to exhibit increased activity under bright outdoor lighting and reduced activity under indoor conditions. The physiological-level reverse potential of mt-EcGAPR ensures minimal oxidative stress. The self-restricting property (Fig. 2k) of EcGAPR limits activity at -216 mV, preventing MMP hyperpolarization beyond this threshold. Moreover, our in vivo data revealed that illumination at low light intensities (~100 lux) had protective effects (Fig. 7). Therefore, at ambient light intensities, the proton pump remains sufficiently active and maintains a safe pmf of approximately −216 mV. While mt-EcGAPR demonstrates sustained photocurrent saturation at about 30 mW/mm^2^ (Supplementary Fig. 2b, equivalent to ~20,490 Lux) and maintains the beneficial pmf around −216 mV under bright outdoor conditions (>10,000 lux), its long-term protective effects and potential adverse outcomes require further systematic evaluation. Additionally, while our current data do not indicate any off-target effects or aberrant expression of mt-EcGAPR at the plasma membrane (Fig. 2l), comprehensive preclinical testing remains essential to identify and reduce potential risks, particularly in the context of AAV-mediated delivery.

As demonstrated, our mt-EcGAPR has three key advantages in applications for eye diseases: genetic-encoded cell specificity, ambient light-mediated activation, and inherent biosafety due to its self-restricting properties. Importantly, our results indicate that mt-EcGAPR may also mitigate age-related vision loss linked to mitochondrial dysfunction and oxidative stress. The optoenergetic activation of mt-EcGAPR suppresses the ER stress–ATF6–pyroptosis axis caused by mitochondrial dysfunction, positioning it as a promising therapeutic agent for not only glaucoma but also other retinal neurodegenerative diseases, such as diabetic retinopathy. Moreover, in PQ-induced zebrafish models, mt-EcGAPR effectively reduces cell death and attenuates inflammatory responses under light stimulation. Intriguingly, a trend toward reduced expression of NLRP3 (NOD-, LRR-, and pyrin domain-containing protein 3), a marker of inflammation, was observed in mt-EcGAPR mice compared with that in C57BL/6 J control mice, although the difference was not statistically significant, suggesting the dominance of the ER stress–ATF6–pyroptosis axis in the early stages of glaucoma (Supplementary Fig. 7h, i).

In summary, our study introduces mt-EcGAPR as a novel approach to bypass defective ETC complexes and provide a safe, beneficial proton motive force under energy-deficient conditions. This strategy offers a new perspective for treating glaucoma and other neurodegenerative disorders associated with mitochondrial dysfunction. Consequently, mt-EcGAPR represents a groundbreaking optoenergetic tool that not only restores mitochondrial function but also mitigates potential phototoxicity risks, making it a versatile platform for treating retinal diseases.

## Materials and methods

### Ethics

All experimental procedures involving animals were conducted in accordance with Shanghai Medical College Institutional Animal Care of Fudan University and the Institutional Animal Care and Use Committee of Zhengzhou University guidelines. All animal experiments were approved by Institutional Animal Care and Use Committee of Fudan University (No. 20211028-016) and conducted in full compliance with its ethical guidelines.

### Plasmid construct and site-directed mutagenesis

Human codon-optimized bacteriorhodopsin (BR, Accession ID: P02945), deltarhodopsin (HtdR, Accession ID: O93740), xanthorhodopsin (AXR, Accession ID: WP_007675008), Gloeobacter rhodopsin (GR, Accession ID: WP_011140202), Leptosphaeria rhodopsin (MAC, Accession ID: AAG01180.1), and Alpha proteorhodopsin (APR, Accession ID: WP_014952819) are synthesized by Genewiz and fused to GFP. Archaeaerhodopsin T (ArchT, Accession ID: ABT17417.1) and Green absorbing proteorhodopsin (GPR, Accession ID: AF349993_1) were obtained from Addgene (Addgene Id: #31177 and #33780, respectively). Coccomyxa rhodopsin (CsR, Accession ID: I0YUS5) was gifted by Dr. Arend Vogt (Insitut für Biologie Experimentelle Biophysik, Charité-Universitätsmedizin Berlin). For membrane targeting, all microbial rhodopsins are cloned into pEGFP-N1 vectors with restriction sites Sal I and BamH I. For mitochondrial targeting, cox8 (NP_004065, 1–29 aa) with 4 repeats is fused to the N-terminal of microbial rhodopsins and cloned to the pEGFP-N1 plasmid with restriction sites Nhe I and Sal I. Chimeric GPR and APR constructs were generated by the overlapping-PCR method. Site-directed mutagenesis was performed by the two-step megaprimer PCR method as previously described.^74^ For expression in *E. coli*, microbial rhodopsins were cloned into the pET28c (+) vector using the restriction sites Nco I and BamH I. For expression in *C. elegans*, the dat-1 promoter (−1326 ~ −1) amplified from the genome using SalI and BamHI was cloned into the ppd95.75 vector, the mt-EcGAPR or 4cox8 gene was linked to the same vector using the restriction sites BamHI and Age I. For lentivirus packaging, mt-EcGAPR is cloned into the pCDH–CMV–puromycin vector using the restriction sites Nhe I and Not I. For expressing in zebrafish, mt-EcGAPR or mt-mt-EcGAPR(D97N) was cloned to pcDNA3.1 using the restriction sites Kpn I and Xba I. For expressing in mice, CAG-mt-EcGAPR-2A-EGFP was cloned into the donor vector using the in-fusion cloning method. All the plasmids were confirmed by sequencing (Genewiz).

### Electrophysiology in HEK cells and neurons

Whole-cell patch clamp recordings were performed using a custom-built opto-electro system that included an Axopatch 700B amplifier (Molecular Devices), a Digidata 1440 A (Molecular Devices) digitizer, an Optoscan (Cairn Research Ltd., UK) monochromator, and an imaging system (Olympus) at room temperature. A customized Micro-Manager software was used to control the devices. Data were acquired at a sampling rate of 10 kHz. Micropipettes were pulled from filamented glass capillaries (Sutter Instrument, BF150-86-10) using a micropipette puller (Sutter Instrument, P1000) to achieve a tip resistance of 4–6 MΩ. The micropipette was filled with intracellular buffer (potassium gluconate 120 mM, KCl 3 mM, HEPES 10 mM, NaCl 8 mM, CaCl_2_ 0.5 mM, EGTA 5 mM, ATP-Mg 2 mM, GTP 0.3 mM, pH 7.2) and positioned using a micromanipulator (Sutter, MP285). The extracellular buffer for recording was Tyrode’s buffer (NaCl 145 mM, KCl 3 mM, HEPES 10 mM, glucose 10 mM, pH 7.4). For recording photocurrents in HEK 293t cells, all-trans retinal (2 μM) was added to the culture media 1 hr after calcium phosphate transfection and cultured for 12–16 h. Photocurrents of HEK 293t cells are recorded at voltage clamp mode and measured at 0 mV unless mentioned. Cultured neurons are transfected at days in vitro (DIV) 4–5 and grown to DIV 8–12 for recording. Neurons were voltage clamped at −70 mV and recorded at current clamp mode. The action spectrum was recorded upon light originating from a Xenon bulb with an intensity of 3.315 mW mm^−2^ through a 40× objective (Olympus). A custom protocol was set to trigger the monochromator to generate light pulses of 300 ms duration with a 20 nm bandwidth and wavelength stepping from 350 nm to 750 nm. Current–voltage (*I*–*V*) curves were obtained by applying voltage steps (−80 mV to 40 mV in 20 mV increments) while stimulating the cells with green light pulses (532 nm laser, 130 mW mm^−2^, 1 s pulse duration, 1 s inter-pulse interval). Light-induced silencing of neurons was investigated by injecting currents (20–180 pA) to elicit action potentials concurrently with green light stimulation (532 nm laser, 130 mW mm^−2^, 1 s pulse duration, 1 s inter-pulse interval).

### Expression in *E. coli*

*Escherichia coli* BL21(DE3) cells, transformed with a pET-28c(+) plasmid harboring the desired microbial rhodopsin gene, were cultured to induce protein expression. Briefly, cells were grown in LB with 30 μg/mL kanamycin at 37 °C and 180 rpm to an OD600 of 0.8. Expression was then induced by adding 10 μM all-trans retinal and 1 mM isopropyl β-D-1-thiogalactopyranoside (IPTG), followed by further incubation for 18 hours at 28 °C and 180 rpm. Bacterial cells were collected by centrifugation and checked for their color changes to determine whether the microbial rhodopsins have been properly expressed.

### pH recording of *E. coli* suspension

*E. coli* expressing microbial rhodopsins were harvested by centrifugation at 8000*g* for 10 min at room temperature. The cells were then resuspended in a buffer-free solution (150 mM NaCl, 50 mM MgSO_4_) to a final concentration of 0.1 g/ml. *E.coli* suspensions were illuminated with a 532 nm laser, and pH changes were monitored using a pH Needle Electrode (Microelectodes) connected to an A/D converter (Digidata 1440 A). Temperature signals was acquired using a temperature sensor connected to a temperature controller (Warner). Data acquisition was performed with AxoScope 10.4 software (Axon Instrument) at a sampling rate of 1 kHz (1000 Hz).

The pH signals were temperature-calibrated by the following formula:

$${\text{pH}}=\frac{{V}_{\mathrm{ref}}-{V}_{\mathrm{pH}}}{2.303R\left(T+273.15\right)/F}$$

where *V*_ref_ is the standard electrode potential when pH is 7.0; *V*_pH_ is the voltage measured by the pH electrode; *R* is the gas constant; *T* is the recorded temperature in degrees Celsius; and *F* is the Faraday constant.

*E. coli* suspension was illuminated with three green pulses (532 nm, 37.18 mW mm^−2^, pulse duration: 2 min, pulse interval: 2 min). Pumping activities were quantified as the peak of pH change during the first 2-min illumination period. All pH changes were normalized to the value of APR. For CCCP treatments, CCCP was added to the *E. coli* suspension to a final concentration of 10 μM and incubated in the dark for 20 min.

### Hippocampal neuron culture and transfection

Hippocampi were dissected from postnatal day 0 (P0) C57BL pups. Briefly, after digestion with trypsin and triturating with fire-polish pipettes, the hippocampal cells recovered by centrifugation were plated onto 12 mm coverslips, and after 2 h, 2 ml plating medium (100 ml of plating medium: 89 ml Minimal Essential Medium (Invitrogen), 0.5 g glucose, 0.5 mM glutamine, 2 g NaHCO_3_, 10 mg bovine transferrin (Calbiochem), 2.5 mg insulin, 10% FBS) was added to each 35 mm dish. From the second day of culture, half of the medium was replaced with feeding medium twice a week (Feeding medium (100 ml): 97 ml minimal essential medium, 0.5 g glucose, 0.5 mM glutamine, 2 g NaHCO_3_, 10 mg bovine transferring, 3 mM cytosine-p-arabinofuranoside (Sigma-Aldrich) and 2% B27 medium supplement (Invitrogen)). For electrophysiology recordings, neurons are transfected at DIV4–5 and cultured to DIV8–12.

### Primary cardiomyocyte culture and transfection

Hearts were dissected from postnatal day 0 (P0) C57BL pups. The hearts were cut into small pieces and washed with HBSS three times. The tissues were digested with trypsin for 5 min and collagenase II (Sigma-Aldrich) for another 30 min. After triturating with fire-polish pipettes, the cells were plated on matrix gel (Corning) coated coverslips. Cardiomyocytes were cultured in plating medium, and half of the medium was replaced with feeding medium twice a week. Cardiomyocytes were transfected by electroporation. Briefly, before cells were plated on coverslips, plasmid DNA (20–30 μg) was prepared in electroporation solution (20 mM HEPES, 135 mM KCl, 2 mM MgCl_2_, 0.5% Ficoll 400, 1% DMSO, pH 7.6, adding 0.2 mM ATP and 0.5 mM glutathione before use). Cells were resuspended in 700 μl electroporation solution and transferred to a BTX cuvette (4 mm gap) for electroporation. The electroporation protocol used was: 180 V, 10 ms pulse duration, 2 pulses with a 1-s interval. After electroporation, cells were resuspended in plating medium and plated onto coverslips following the standard procedure. Cardiomyocytes were ready to express the transfected plasmid at DIV3.

### Cell line culture and transfection

HEK293t, COS-7, and HeLa cells were grown in Dulbecco’s modified Eagle medium (DMEM, Gibco) supplemented with 10% FBS. Cells were maintained at 37 °C in a humidified incubator with 5% CO_2_ and 95% air. For transient expression, cells are transfected using the calcium phosphate precipitation method.

### Calcium phosphate precipitation transfection

Cells were grown in Dulbecco’s modified Eagle medium (DMEM, Gibco) supplemented with 10% FBS to a confluence of 60–70%. Cells were transferred to serum-free DMEM for 40 minutes. The calcium transfection mixture was prepared by combining 10 μg of plasmid DNA, 5 μl of 2.5 M CaCl_2_, and ddH_2_O to a final volume of 40 μl. To this mixture, 40 μl of 2×HEBS (274 mM NaCl, 10 mM KCl, 1.4 mM Na_2_HPO4, 15 mM D-glucose, 42 mM HEPES, pH 7.07) was added dropwise while vortexing. After 13 min, the mixture was added dropwise to the cells in serum-free DMEM, incubated for 1 h at 37 °C with 5% CO_2_. Following incubation, the serum-free DMEM was removed, and the cells were treated with 15% glycerol (in PBS, v/v) for 30 s. The cells were then washed once with PBS and subsequently cultured in DMEM supplemented with 10% FBS at 37 °C with 5% CO_2_.

### Stable transfection cell line construction

Stable transfected cell lines were constructed by electroporation or lentivirus transduction, followed by screening. Vectors used for stable transfection were pEGFPN1 for electroporation and pcdh-puromycin for lentivirus transduction, respectively. For electroporation, 10^6^ cells are harvested by trypsin digestion and centrifugation. Cells were resuspended in PBS and subjected to electroporation using a BTX electroporation system. The protocol used was: 175 V, 100 ms pulse duration, 1 s interval, 2 pulses, in a 4 mm gap cuvette. Following electroporation, cells were plated and cultured in complete DMEM supplemented with G418 (0.7 mg/ml, Gibco) for selection. Untransfected cells gradually died off over time. Single clones that survived were isolated and expanded in 10% FBS DMEM containing 0.7 mg/ml Geneticin (G418) for at least 3 weeks. Clones were checked for expression level by fluorescence confocal microscopy. Clones with the highest expression level were used for further study and maintained in 10% FBS DMEM without Geneticin.

For lentivirus transduction, the virus was added to the medium, and cells were cultured for 3 days for virus infection and expression. For stable screening, puromycin (20 μg/mL) was added to the medium for sustained culture. All the genomic DNA of the stable transfected cell lines is extracted to verify the existence of the plasmid sequence by PCR and sequencing (Genewiz).

### Confocal imaging and colocalization analysis

All images were acquired with fluorescence laser scanning confocal microscopy (FV1000, Olympus) at room temperature. A 60×/1.2w objective (Olympus) is used for colocalization analysis. For mitochondrial colocalization evaluation, confocal images were cropped to a small area containing a single cell using Image J (NIH). Images are de-noised using the ‘Despeckle’ command. Plugin Colocalization Indices^75^ was used for colocalization analysis, and the Pearson’s correlation coefficients (CC) were obtained to assess mitochondrial targeting efficiency.

### Cluster analysis

A K-Means clustering analysis was performed on the Pearson’s correlation coefficients and normalized ΔpH values of chimeras using customized Python scripts. Before clustering, the data underwent standardization to normalize the data; the standardization formula used is:

$${X}_{\mathrm{scaled}}=\frac{X-\mu }{\sigma }$$

where *X* is the variable value, *μ* is the mean, and *σ* is the standard deviation.

Subsequently, the K-Means algorithm was executed to partition the data into *k* = 4 clusters such that the within-cluster sum of squares is minimized, as articulated by the optimization problem:

$$\mathop{\min }\limits_{{\mu }_{1},\ldots ,{\mu }_{k}}\mathop{\sum }\limits_{{\rm{i}}=1}^{{\rm{k}}}\mathop{\sum }\limits_{x\epsilon {s}_{i}}{{\rm{||}}x-{\mu }_{i}{\rm{||}}}^{2}$$

where S_*i*_ is the subset of data points assigned to cluster *i*, and *μi* is the centroid of cluster *i*. The algorithm iteratively adjusts the centroids and reassigns data points to clusters until convergence is achieved. A scatter plot was generated using matplotlib, with each cluster represented by distinct colors and error bars indicating the standard error of the mean (SEM).

### Mitochondrial membrane potential imaging

The mt-EcGAPR-EGFP stable transfected COS7 cells were loaded with rhodamine 800 (50 nM) for 20 min in Tyrode’s buffer. Cells were imaged with a laser scanning microscope (Olympus FV1000, Japan) in Tyrode’s buffer at room temperature. To achieve region-specific light stimulation, a region of interest (ROI) containing a single cell was selected on the confocal image. The selected cell was then illuminated with a confocal 515 nm laser in bleaching mode for 320 s. For measuring the mitochondrial membrane potential, the following formula was used:

$$\psi =-\frac{{RT}}{F}\mathrm{ln}\left(\frac{{F}_{\mathrm{sig}}}{{F}_{\mathrm{bg}}}\right)$$

where *T* is the absolute temperature, *R* is the gas constant, *F* is the Faraday constant, and *F*_bg_ and *F*_sig_ represent the fluorescence of the image in the background and inside the mitochondria, respectively. The images were analyzed with ImageJ (NIH). Briefly, the confocal image was first background-subtracted using the average background signal. It was then converted to a 16-color pseudo-color image. Contrast and brightness adjustments were applied to make the pixel with the highest signal intensity appear white. The mitochondrial fluorescence signal (*F*_sig_) for a single cell was quantified by drawing an ROI that encompassed the mitochondria within the cell. Background signal (*F*_bg_) was measured by drawing an ROI in an area devoid of cells. The MMP was calculated as follows, with a temperature value of 298 K (25 °C):

$$\psi =-59{\log }_{10}\left(\frac{{F}_{\mathrm{sig}}}{{F}_{\mathrm{bg}}}\right)$$

### Mitochondria isolation

Mitochondria were isolated from cells using differential centrifugation. Briefly, cells were digested with trypsin and collected by centrifugation at 1000 rpm for 5 min at 4 °C. The cell pellet was washed twice with PBS and resuspended in mitochondrial isolation buffer (250 mM sucrose, 5 mM HEPES, 1 mM EGTA, pH 7.4). The suspension was then manually homogenized in a glass homogenizer on ice (4 °C) using a limited number of strokes (approximately 20) with trypan blue added to monitor cell lysis. Homogenization was stopped when ~90% of cells were stained blue, indicating successful disruption of the plasma membrane. The homogenate was then transferred to a 1.5 ml EP tube and centrifuged at 3000 rpm for 5 min at 4 °C. The supernatant containing the mitochondria was collected, and the pellet containing undigested cells and nuclei was discarded. The supernatant was further centrifuged at 3000 rpm for 5 min at 4 °C to remove any cellular debris. Finally, crude mitochondria were obtained by centrifuging the supernatant at 13,000 rpm for 30 min at 4 °C.

### Submitochondrial fractionation

Following isolation by differential centrifugation, mitochondria were washed twice with mitochondria isolation buffer and centrifuged at 13,000 rpm for 5 min each time to remove residual contaminants. To isolate submitochondrial compartments, mitochondria were then subjected to digitonin extraction. Mitochondria were resuspended in mitochondria isolation buffer containing 0.4 mg/mL digitonin and incubated on ice for 15 min. Digitonin selectively permeabilizes the outer mitochondrial membrane (OMM). Subsequently, the suspension was centrifuged at 13,000 rpm for 10 min at 4 °C. The resulting pellet (mitoplast fraction) contained the inner mitochondrial membrane (IMM) and matrix, while the supernatant contained solubilized OMM and intermembrane space (IMS) proteins. Mitoplast proteins were further fractionated using the Membrane and Cytosol Protein Extraction Kit (Beyotime) according to the manufacturer’s instructions. Briefly, the mitoplast was resuspended in membrane protein extraction reagent A, followed by sonication to disrupt the IMM. The lysate was then centrifuged at 14,000 rpm for 40 min at 4 °C. The supernatant (matrix fraction) contained soluble matrix proteins, whereas the pellet (membrane fraction) was enriched for IMM proteins. Finally, the membrane fraction was solubilized in membrane protein extraction reagent B for western blotting. The purity of various components of the extracted mitochondrial substructures was analyzed by immunoblotting using TOM20 (Santa Cruz, sc-17764, 1:500, mouse) as the mitochondrial outer membrane marker, Hsp60 (Enzo, ADI-SPA-806-F, 1:1000, mouse) as the mitochondrial matrix marker, and mitofilin (Proteintech, 10179-1-AP, 1:1000, rabbit) as the mitochondrial inner membrane marker, GAPDH (Abcam, ab9484, 1: 1000) as the cytosol marker.

### Topology assay

To determine the topology of mt-EcGAPR on the inner mitochondrial membrane, the C terminus of mt-EcGAPR was tagged with EYFP or ECFP. These constructs were co-transfected into COS-7 cells via the calcium phosphate precipitation method. Given that EYFP exhibits pH-sensitive fluorescence, while ECFP fluorescence remains stable across a range of pH values, the EYFP/ECFP fluorescence ratio serves as a reliable pH indicator. The protonophore CCCP was utilized to induce pH alterations by causing acidification of the mitochondrial matrix and alkalinization of the intermembrane space. The fluorescence changes were monitored using a confocal laser scanning microscope (FV1000, Olympus). To validate the pH alterations, 4cox8-EYFP/ECFP was used as matrix indicators, and GPD (glycerol-3-phosphate dehydrogenase)-EYFP/ECFP was used as intermembrane space indicators.

### Immunoblotting

HEK293t cells expressed with mt-EcGAPE-EGFP were analyzed for Western blotting. Cells were cultured until the confluence reached 70–80%. Transfection was performed using the QuickShuttle transfection reagent (Biodragon). The transfection mixture was prepared by combining 2 μg of plasmid with 4 μl of QuickShuttle, each diluted in 50 μl of 0.85% NaCl, resulting in a final volume of 100 μl. The mixture was added to the cell culture and incubated at 37 °C for 24 h. Transfected cells were examined under a microscope to confirm plasmid transfection expression, cell death, and floating conditions. The cells were washed with PBS, and 100 μl of cell lysis buffer (1% Triton, 1% DOC, 100 mM PMSF, in pH 7.4 TBS) was added to a 35 mm culture dish. The cells were slowly shaken on ice for 30 min and then centrifuged at 12000 rpm for 10 min at 4 °C, and the supernatant was collected.

Protein concentrations were determined using the BCA method, and the lysates were adjusted to equivalent protein concentrations. An appropriate amount of protein was mixed with 5× reducing loading buffer and centrifuged at 13,000 rpm for 5 min. Microbial rhodopsins are highly hydrophobic membrane proteins; the proteins cannot be boiled at 100 °C, otherwise they will precipitate. The protein samples were loaded onto a polyacrylamide gel and subjected to electrophoresis at 130 V for 80 min. The resolved proteins were then transferred onto a nitrocellulose (NC) membrane at 100 V for 120 min. The membrane was blocked with 10% non-fat milk in Tris-buffered saline with Tween-20 (TBST) for 1–2 h at room temperature, followed by an overnight incubation at 4 °C with the primary antibody. After three washes with TBST for 5 min each to remove unbound primary antibody, the membrane was incubated with a secondary antibody conjugated to horseradish peroxidase, diluted 1:2000 in PBS, for 1–2 h at room temperature. The membrane was washed three times with TBST for 5 min each time, and the target band was detected by ECL luminescence.

### ROS and DNA damage imaging

Stable transfected cell lines are illuminated with a custom-made programmed green LED (530 nm, 1000 lux, 900 ms ON, 100 ms OFF) in a CO_2_ thermostat incubator. For ROS imaging, after illumination for 4 h, cells are stained with mitoSOX (5 μM, Invitrogen), a mitochondrial superoxide indicator, or dihydroethidium (DHE, 5 μM, Invitrogen), a cytosol ROS indicator. The cells were imaged with a fluorescence microscope (Olympus IX83, Japan). For DNA damage imaging, after illumination for 16 h in the incubator, cells are fixed and stained with γ-H2AX antibody (CST, mouse, 1:400) or 8-oxoguanine antibody (MAB3560, Sigma-Aldrich, mouse, 1:200).

### SNARF-1-AM staining

For pH imaging, isolated mitochondria were loaded with SNARF-1-AM (Invitrogen) to a final concentration of 20 μM in mitochondrial isolation buffer for 20 min at 37 °C. After loading, the mitochondria were centrifuged at 12,000 rpm and resuspended in mitochondrial isolation buffer. SNARF-1 AM was excited by 559 nm, and emission was collected at 580–600 nm and 640–700 nm. The pH was determined by calculating the ratio of the two emission channels.

### ATP assay

COS-7 cells stably transfected with mt-EcGAPR were cultured in Dulbecco’s Modified Eagle Medium (DMEM) supplemented with 10% FBS until reaching 70–80% confluence. Before the experiment, the growth medium was replaced with either high glucose DMEM or low glucose DMEM, both supplemented with 10% FBS and 1 μM retinal, and the cells were incubated for 18 h at 37 °C. Subsequently, the cells were transferred to Tyrode’s solution or PBS, respectively, each supplemented with 12 mM calcium (Ca^2+^) and 12 mM magnesium (Mg^2+^), and exposed to green light (532 nm, 0.708 mW/cm²) at room temperature (25 °C) for 2 h. Intracellular ATP levels were quantified using the ATP Assay Kit (Beyotime) following the manufacturer’s protocol.

### Succinate and NAD^+^/NADH assay

Mt-EcGAPR stable-transfected cells were seeded on 3.5 cm culture dishes and grown in 10% FBS DMEM to 70–80% confluence. Before light treatment, 1 μM all-trans retinal was added. Cells were then treated with either 1 μM rotenone (Sigma-Aldrich) or DMSO control, followed by illumination with green light (530 nm, 1000 lux, 900 ms ON, 100 ms OFF) at 37 °C for 18 h. Cells were trypsinized, harvested by centrifugation, and assayed for succinate using the Succinate Assay Kit (ab204718, abcam) and NAD^+^/NADH levels using the NAD^+^/NADH Assay Kit (ab65348, abcam) according to the manufacturer’s instructions.

### Nematode stains and transgenic *C. elegans*

The wild-type Bristol N2 strains were obtained from the Caenorhabditis Genetics Center. Nematodes were cultured at 20 °C on standard nematode growth medium (NGM) plates seeded with *E. coli* OP50. Transgenic worms were generated following the standard protocol by injecting the respective plasmids into N2 worms. Following is detailed information of transgenic strains: SQC0210 yfhEx0210 (*Pdat-1::mt-EcGAPR::GFP* at 10 ng μl^−1^; *Pdat-1::Tomm20::mCherry* at 10 ng μl^−1^); SQC0211 yfhEx0211 (*Pdat-1:: 4cox8::GFP* at 10 ng μl^−1^; *Pdat-1::mCherry* at 10 ng μl^−1^). SQC0212 yfhEx0212 (*Pdat-1::mt-EcGAPR::GFP* at 10 ng μl^−1^; *Pdat-1::mCherry* at 10 ng μl^−1^).

### Rotenone treatment

The nematode growth medium (NGM) was supplemented with rotenone to a final concentration of 4 μM (rotenone concentrations exceeding 8 μM affected the reproduction of *C. elegans*), and all-trans retinal was added to the *E. coli* OP50 at a final concentration of 500 μM. The F1 *C.elegans* with positive expression were picked to the NGM containing rotenone and retinal food. Synchronized worms were obtained by bleaching the adults using hypochlorite/NaOH. L4 stage worms were loaded on NGM plates with rotenone. These worms were cultured for 5–7 days and examined for the degeneration of dopamine neurons. For light treatment, NGM plates were seeded with *E. coli* OP50 supplied with 500 μM ATR or DMSO. Worms were incubated in the dark for 1 day for uptake of the ATR and then illuminated with green light (~540 nm, 10000 lux, 900 ms ON, 100 ms OFF) until the day of neuron examination. The light was pulsed at a frequency of 1 Hz (900 ms ON, 100 ms OFF) to prevent inactivation of the proton pump and rotenone. For examination, 50 worms were picked using a worm picker and immobilized in 2% sodium azide, pre-dropped on a 2% agarose pad. The cephalic dendrites (CEPs) from the nerve ring to the nose tip were examined. For clearly counting the number of survival CEPs, we used the yfhEx0212 strain. The cephalic dendrites (CEPs) from the nerve ring to the nose tip were examined. Confocal scanning microscopy was employed to record images using Z-stack settings with 0.1 μm intervals along the *z*-axis, and images from different layers were merged to quantify CEP neuron numbers.

### Locomotion assay of *Caenorhabditis elegans*

Light (~540 nm, 10000 lux, 900 ms ON, 100 ms OFF) was illuminated before behavior analysis. After light stimulation, *C. elegans* were analyzed for locomotion activity. In the behavior assay, a single worm was picked on a fresh NGM plate without rotenone and imaged for 2 min using a stereo microscope (Stemi 508, ZIESS).

### Zebrafish maintenance

Adult zebrafish were maintained at 28.5 °C on a 14 h light/10 h dark cycle. Embryos were maintained at 28.5 °C in fish water (0.2% Instant Ocean Salt in deionized water).

### Acridine orange staining

Fertilized one-cell zebrafish embryos were injected with 100 pg pcDNA3 containing mt-EcGAPR or mt-EcGAPR(D97N) at a concentration of 50 ng/μL. Exposure of zebrafish embryos to paraquat (100 μg/mL) can induce a robust apoptosis.^32^ To assess the effects of mt-EcGAPR or mt-EcGAPR(D97N) on PQ-induced apoptosis, 2-day zebrafish were treated with vehicle control (fish water), PQ (100 μg/mL) or PQ (100 μg/mL) plus D97N (100 pg per embryo) or plus mt-EcGAPR (100 pg per embryo) with green LED (~540 nm, 10,000 lux, 900 ms ON, 100 ms OFF) for 24 h. All embryos were incubated at 28.5 °C. After treatment, 3-dpf embryos were immersed in 5 μg/ml AO (acridinium chloride hemi-[zinc chloride], Sigma-Aldrich), a nucleic acid intercalating dye that selectively binds to dying cells’ double-stranded DNA, in fish water for 60 min. Next, zebrafish were rinsed thoroughly in fish water three times (5 min/wash) and anaesthetized with 0.016% MS-222 (tricaine methanesulfonate, Sigma-Aldrich, St. Louis, MO). Zebrafish were then oriented on their lateral side and mounted with 3% methylcellulose in a depression slide for observation by fluorescence microscopy.

### Zebrafish in vivo macrophage migration assays

The monitor the macrophages, the *TG(zlyz:EGFP)* transgenic lines were used, which have been described previously.^32^ Fertilized one-cell *TG(zlyz:EGFP)* transgenic zebrafish embryos were injected with 100 pg pcDNA3 containing mt-EcGAPR or mt-EcGAPR (D97N) at a concentration of 50 ng/μL. At 2-dpf, embryos were treated with 100 μg/mL paraquat (PQ) for 48 h in a 12-well plate format (thirty embryos per well) (BD Falcon). Exposure of zebrafish embryos to paraquat (100 μg/mL) can induce a robust macrophage migration.^76^ To assess the effects of mt-EcGAPR or mt-EcGAPR (D97N) on PQ-induced macrophage migration, 2-day zebrafish were treated with vehicle control (fish water), PQ (100 μg/mL) or PQ (100 μg/mL) plus mt-EcGAPR(D97N) (100 pg per embryo) or plus mt-EcGAPR (100 pg per embryo) with green LED (~540 nm, 10,000 lux, 900 ms ON, 100 ms OFF) for 48 h. All embryos were incubated at 28.5 °C. After treatment, 4-dpf embryos were washed with fish water three times and anaesthetized with 0.016% MS-222 (tricaine methanesulfonate, Sigma-Aldrich, St. Louis, MO), and the number of macrophages recruited to the body trunk was counted.

### Zebrafish image acquisition

Embryos and larvae were analyzed with a Nikon SMZ18 Fluorescence microscope and subsequently photographed with digital cameras. The quantitative assessment of the images was performed using morphometric analysis software, NIS-Elements D4.6 (Japan) and ImageJ (NIH, USA). Inverted fluorescent images were used for processing. Positive signals were defined by particle number using ImageJ. Ten animals for each treatment were quantified, and the total signal per animal was averaged.

### Virus preparation

Lentivirus was produced from 293t cells transfected with gene plasmid, capsid (pMD2.G), and helper plasmids (psPAX) by the calcium phosphate precipitation method. Viruses were harvested from cell medium and concentrated with an ultrafiltration membrane (100 K, Millipore, USA). AAV DJ serotype virus was utilized for transfecting the retina in mice. To produce the AAV virus, gene plasmid, capsid (pAAV-DJ), and helper plasmids (pHelper) were co-transfected into 293t cells through the calcium phosphate precipitation method. After 48 h of transfection, viruses were harvested from cell pellets using four cycles of frozen-thaw methods. The titers of the virus were determined by quantitative real-time PCR.

### Knock-in mouse generation

The CAG-mt-myc-EcGAPR-2A-EGFP-WPRE-polyA gene was targeted to the Rosa26 locus using the CRISPR/Cas9 system. Briefly, gRNA and Cas9 mRNA were obtained by in vitro transcription. Donor vector containing target gene CAG-mt-myc-EcGAPR-2A-EGFP-WPRE-polyA, 5’ and 3’ homologous arms was constructed by the in-fusion cloning method. A mouse fertilized egg was injected with gRNA and Cas9 mRNA, and the donor vector. F0 mice were identified and crossed with WT mice to generate positive F1 mice.

### Immunohistochemistry and cell counting

For whole-mounted retinae, mice were perfused with PBS and then 4% PFA (Sigma, US) at different time points after SO injection. Eyes were enucleated and fixed in 4% PFA for 0.5 h. Retinae were then dissected and fixed in 4% PFA overnight at 4 °C. Retinae were washed with PBS, incubated in 0.5% TritonX-100 for 1 h, and then blocked with 10% DST (Donkey Serum in Tris-buffered saline) for 2 h at room temperature. Then retinae were transferred into the primary antibody (anti-Brn3a antibody, Santa Cruz Biotechnology (SC-31984), 1:500) solution and incubated for 30–36 h at 4 °C. After washed in PBS, retinae were transferred to the secondary antibody (Donkey anti-Goat conjugated to Alexa Flour 594, 1:800, Jackson ImmunoResearch, USA) solution and incubated for 2.5 h at room temperature in the dark. After the secondary antibody was washed off, whole-mounted retinae were air dried and mounted with AQUA-MOUNT (Thermo Scientific, US). Confocal images were obtained by a confocal multi-photon scanning microscope (AIR-MP, Nikon Inc., Japan) or a confocal laser scanning microscope (FluoView FV3000, Olympus, Japan) and processed by ImageJ software (NIH).

For retinal frozen section immunostaining, mice were perfused with PBS and then 4% PFA (Sigma, US). Eyes were enucleated, and retinae were dissected. Retinae were fixed in 4% PFA overnight at 4 °C. 10%, 20% and 30% sucrose solutions were used in turn to dehydrate the fixed retinae. Retinae were embedded in OCT compound (Sakura) and stored at −80 °C overnight. Sections of 14 μm were cut by a cryostat (Leica CM 1950, Leica, Germany). Slices were washed with PBS 3 times (15 min per time), incubated in 0.05% TritonX-100 for 0.5 h, and blocked with 10% DST for 1 h at room temperature. Then slices were then incubated with the primary antibody (anti-GFP antibody, Aves Labs, Inc. (GFP-1020), 1:500; anti-Brn3a antibody, Santa Cruz Biotechnology (SC-31984), 1:500) solution for 20–24 h at 4 °C. After incubation, the slices were washed 3 times (15 min per time) using PBS and incubated with secondary antibody (Donkey anti-Chicken conjugated to Alexa Flour 488, 1:800, Jackson ImmunoResearch, USA; Donkey anti-Mouse conjugated to Alexa Flour 647, 1:800, Jackson ImmunoResearch, USA; Donkey anti-Goat conjugated to Alexa Flour 594, 1:800, Jackson ImmunoResearch, USA) solution at room temperature for 2.5 h in dark. Then the slices were washed 3 times (15 min per time), stained in 1:3000 DAPI solution for 10 min, and washed 3 times (15 min per time). Slices were air dried and mounted with AQUA-MOUNT (Thermo Scientific, US). Confocal images were obtained by a confocal multi-photon scanning microscope (AIR-MP, Nikon Inc., Japan).

To perform cell counting of Brn3a-positive retinal ganglion cells (RGCs), the retina was divided into four sections and subsequently flattened. Within each section, two square fields measuring 0.05 mm^2^ were chosen for analysis, one located in the central area (800 μm away from the optic papilla) and another in the peripheral area (1600 μm away from the optic papilla). The cell numbers within these fields were quantified, averaged across the entire retina, and used to calculate the ratio of Brn3a-positive RGC density between eyes with silicone-oil-injected eyes (SO eyes) and contralateral eyes (CL eyes).

### Ocular hypertension mouse model

Glaucoma was induced using a modified SO injection model as previously described.^37^ Briefly, mt-EcGAPR mice and C57BL/6 J mice aged from 9 to 12 weeks were anesthetized by intraperitoneal injection of 1.25% Avertin (Nanjing Aibei Biotechnology Co., Ltd., China). Mice were placed in their left-lateral position 5–10 min after injection. One drop of sodium hyaluronate was applied onto the cornea of the right eye to keep it moist during the whole procedure. A 32 G × 4 mm needle was used to pierce through the cornea close to the limbus at the superotemporal side, reaching the anterior chamber with the least injury. After the aqueous humor was drained, silicone oil (SO) (Arciolane 1300 Syringe 10 mL, ARCADOPHTA SARL, Toulouse, France) was injected into the anterior chamber through the puncture point using a homemade device. The injection was stopped when the SO covered most areas of the iris. Levofloxacin hydrochloride eye gel (0.015 g in 5 g) was then applied onto the injected right eye to prevent infection.

### Light stimulation

During light stimulation, mice were placed in a customed LED acrylic cylinder with a diameter of 26 cm, and food and water were available over the upper end of the cylinder. The cylinder walls were lined with LED strips emitting 530-nm light at an intensity of 7200 lux. The environmental illuminance was calculated as the average illuminance detected near the cylinder wall (four directions).

### Mice DHE staining

Mice were injected intravenously with DHE (20 mg/kg) via the tail vein. 90 minutes post-injection, mice were anesthetized and perfused with PBS, followed by 4% paraformaldehyde (PFA). Eye cups were then dissected, immersed in 4% PFA overnight at 4 °C, and subsequently dehydrated in a graded sucrose series (10%, 20%, and 30%). Finally, the eye cups were embedded in optimal cutting temperature (O.C.T) compound and stored at −80 °C.

### Mito-TEMPO treatment

Mito-TEMPO was dissolved in phosphate-buffered saline (PBS) and administered intraperitoneally daily at a dose of 0.7 mg/kg following SO injection.

### Retinal ATP measurement

Mice were anesthetized with an intraperitoneal injection of 1.25% Avertin. Whole retinae were dissected and placed in 1.5 mL Eppendorf tubes containing 150 μL of ATP lysis buffer. The samples were then subjected to ultrasonication. Subsequently, the suspensions were centrifuged at 12,000 rpm for 10 min, and the supernatants were collected. Retinal ATP level was measured in the supernatants according to the manufacturer’s instructions of the ATP assay kit (Beyotime). Total protein concentration in each sample was determined using a BCA protein assay kit (Thermo). Finally, the ATP level per retina was calculated by dividing the measured ATP content by the total protein content.

### Optical coherence tomography

Mice were anesthetized with an intraperitoneal injection of 1.25% Avertin. Compound tropicamide eye drops were then applied to dilate the pupils. Once pupillary dilation was complete, mice were positioned in front of the camera. Scanning OCT images were acquired across the optic papilla for subsequent analysis.

### Seahorse mito-stress assay

Mito-stress test was performed by Seahorse Extracellular Flux Analyzer (Agilent Technology). Mice were anesthetized with an intraperitoneal injection of 1.25% Avertin. Several fresh retinal patches (1-mm diameter) were obtained and stored in oxygenated AMES solution (120 mmol/L NaCl, 22.6 mmol/L NaHCO_3_, 3.1 mmol/L KCl, 0.5 mmol/L KH_2_PO_4_, 1.5 mmol/L CaCl_2_, 1.2 mmol/L MgSO_4_, and 6 mmol/L glucose). Retinal patches were then transferred to the islet plate filled with intracellular fluid (135 mmol/L NaCl, 10 mmol/L HEPES, 3.1 mmol/L KCl, 0.5 mmol/L KH_2_PO_4_, 1.5 mmol/L CaCl_2_, 1.2 mmol/L MgSO_4_, 6 mmol/L glucose, pH 7.4 adjusted with NaOH) and equilibrated in a 37 °C incubator for 30 minutes. Concurrently, a standard plate was loaded, and calibration was initiated. Upon completion of calibration, oligomycin (20 μM), FCCP (2 μM), rotenone, and antimycin A (1.5 μM) were pre-added to the islet plate and then loaded together with the islet plate.

### RNA sequencing and data analysis

For bulk RNA-seq, whole fresh retinae were dissected immediately and transferred to 1.5 mL Eppendorf tubes. The retinae were then frozen in liquid nitrogen and sent to Novogene for sequencing. Differentially expressed genes were identified using DESeq2 (an R package) based on the count files provided by Novogene. Gene Ontology (GO) and Kyoto Encyclopedia of Genes and Genomes (KEGG) pathway analyses were performed using GProfiler (https://biit.cs.ut.ee/gprofiler/gost). Enrichment analysis based on the Molecular Signatures Database (MSigDB) was conducted using Enrichr (https://maayanlab.cloud/Enrichr/).

### Mitochondrial protein mass spectrometry analysis

For mitochondrial proteomic analysis, fresh retinae were dissected immediately to minimize protein degradation. Mitochondria were isolated using the Mitochondria Isolation Kit (Thermo, Cat# 89874) according to the manufacturer’s instructions. Following isolation, the mitochondrial suspension was sonicated and centrifuged at 12,000 rpm for 10 min. The supernatant was collected, and the total protein concentration was measured by a BCA assay kit. The protein concentration of the supernatant was then adjusted to 1 mg/ml. Samples were subsequently sent to the Core Facility of Shanghai Medical College, Fudan University, for further analysis.

Based on the resulting spectral data, orthogonal partial least squares discriminant analysis (OPLSDA) was employed to identify differentially expressed proteins. A VIP (variable importance in projection) value exceeding 1 was used as the cutoff for significance. Subsequently, custom Matlab and R scripts were utilized to identify differentially expressed proteins across groups, considering a *p*-value threshold of 0.05 and a fold-change greater than 1.4.

Functional enrichment analysis was performed to elucidate the biological processes associated with the identified differentially expressed proteins. Gene Ontology (GO) and Kyoto Encyclopedia of Genes and Genomes (KEGG) pathway enrichment analyses were conducted using GProfiler (https://biit.cs.ut.ee/gprofiler/gost). Additionally, enrichment analysis based on the Molecular Signatures Database (MSigDB) was carried out using Enrichr (https://maayanlab.cloud/Enrichr/).

### Western blot of retina

At different time points after SO injection, mice were anesthetized, and fresh retinae were dissected and placed into the pre-cooled EP tubes containing 150 μL RIPA lysis buffer supplemented with protease and phosphatase inhibitors (RIPA: protease inhibitor: phosphatase inhibitor = 20:3:2). The samples were subjected to ultrasonication, followed by centrifugation at 12,000 rpm for 15 min. The supernatant was collected, and the total protein concentration was measured using a BCA kit. Based on the calculated concentration, the supernatant was diluted with RIPA lysis buffer and 5× loading buffer to a final concentration of 20 μg/mL. The diluted samples were heated at 99.9 °C for 10 min and then aliquoted and stored at −80 °C.

For Western blotting, the frozen samples were reheated at 99.9 °C for 4 min and centrifuged slowly. 15 μL samples were separated on SDS-PAGE gels and transferred to PVDF membranes. Membranes were blocked, incubated with primary antibodies (rabbit anti-BIP, ATF6, IRE1α, PERK, GSDMD, IL18, IL1β, and mouse anti-β-actin at 1:1000 dilution), followed by incubation with appropriate HRP-conjugated secondary antibodies. Protein bands were visualized using ECL, imaged with E-blot (Yibote, China), and quantified using ImageJ software.

### IOP measurement

The IOP was measured by a TonoLab tonometer (Colonial Medical Supply, Espoo, Finland) weekly after SO injection. Mice were anesthetized by intraperitoneal injection of 1.25% Avertin, and IOP measurements were taken approximately 20 min later. The average of 6 continuous IOP readings was calculated as one measurement, and three such measurements were obtained at 5-min intervals.

### Visual acuity and contrast sensitivity test

Visual acuity was assessed using an opto-motor response (OMR) system comprising four LCD monitors arranged in a square, with a camera positioned above to track the mice. Black and white drifting gratings at 100% contrast were presented clockwise (for left eye) or counterclockwise (for right eye) at a constant speed (12 degrees/s) on the monitors, generated by the Psychopy toolbox. All mice were habituated one day before the test. Mice were placed unrestrained on a pedestal in the center of these 4 monitors. In the beginning, screens were masked with gray to calm the mice down. When mice stopped moving, the gray background was replaced by drifting gratings at a low spatial frequency (about 0.1 cycle/degree). Once tracking behavior was observed, the spatial frequency was increased in the following successive trials until the maximum frequency was identified. The maximum spatial frequency of the silicone-oil-injected eye and the contralateral eye was recorded, respectively. The results were expressed as the percentage of visual acuity in SO eyes relative to CL eyes.

For contrast sensitivity testing, the spatial frequency was fixed at 0.031, 0.044, 0.064, 0.092, 0.103, 0.192, 0.272, 0.35, and 0.38 cycles/degree. The lowest contrast of each spatial frequency that could be detected by the mice was recorded, and the reciprocal of this value was calculated as the contrast sensitivity.

### Intravitreous injection of AAVs

C57BL/6 J mice aged from 6-8 weeks were anesthetized by intraperitoneal injection of 1.25% Avertin (Nanjing Aibei Biotechnology Co., Ltd., China). A glass pipette (3.5” Drummond # 3-000-203-G/X, Drummond Scientific Company, USA), filled with the viral solution, was carefully inserted into the vitreous cavity of each eye. A volume of 1.8 μl of viral solution (3 × 10^10^ gc/mL, genome copies per milliliter) was then injected into the eye using NanoJect II (Drummond Scientific Company, USA). The pipette was left in place for 10 s before being withdrawn. After 3 weeks, allowing for expression of the injected AAVs, the treated mice were utilized in subsequent experiments related to glaucoma.

### Ambient light exposure

The mt-EcGAPR mice were maintained under a standard 12-h light–dark cycle. During the light period, ambient light intensity varied within the cage: front (107 Lux), back (4 Lux), left (12 Lux), right (23 Lux), and center (4 Lux).

### Quantification and statistical analysis

Data analysis was performed using MATLAB (MathWorks, USA), ImageJ (NIH, USA), Excel (Microsoft, USA), Seahorse Wave (Agilent), pClampfit (Molecular Devices), Perl, and Python as specified. Data are presented as mean ± SEM unless otherwise noted. Normality and homoscedasticity of data were assessed. For data that did not meet these assumptions, non-parametric tests were used. Otherwise, Student’s *t*-tests were employed. Statistical significance is indicated as follows: n.s. (*p* > 0.05), *p < 0.05, **p < 0.01, ***p < 0.001, ****p < 0.0001. Detailed information on sample sizes and statistical tests for each experiment is provided in the figure legends and the main text.

All electrophysiology data are analyzed by pClampfit (Molecular Devices) and customized Perl scripts. For action spectrum analysis, the photocurrents at each wavelength of light pulses were averaged and plotted as a single point. The photocurrents were normalized to the range of 0–1. The points of different cells are averaged and presented as mean ± SEM. For *I*–*V* curve analysis, the photocurrents of each light pulse, held at the same voltage, are averaged and subtracted from the baseline current. All the photocurrents were normalized to the photocurrents at 0 mV. The normalized photocurrent points of different cells were averaged and presented as mean ± SEM. The theoretical reversal potential was derived from linear fitting to the data. For displaying the data of light-induced neuron silencing, the data of 5 light pulses were displayed. For displaying the photocurrents of the chimera 1457 and EcGAPR, the photocurrents under a single fixed light pulse were extracted and subtracted from base baseline current. For light-induced pH changes analysis, the maximum pH changes of the first light pulse were all normalized to the value of APR. The normalized pH changes were presented as mean ± SEM.

## Supplementary information

Supplementary_Materials

Raw WB results

## Data Availability

All data supporting the findings of this study are available within the article and its Supplementary Information files. Please contact Jian-Sheng Kang (kjs@zzu.edu.cn) for reagents and resources generated in this study. Plasmid and mice generated in this study are available from the lead contact upon request. Bulk RNA-seq data have been deposited at https://ngdc.cncb.ac.cn/omix, accession number: OMIX011940. Mitochondrial protein mass spectrum data have been deposited at https://ngdc.cncb.ac.cn/omix, accession number: OMIX009216.
