# Supplementary material for: Ambient light alleviates retinal neurodegeneration in mice by powering mitochondria via the engineered optoenergetic rhodopsin
Source: Signal Transduct Target Ther. 2025 Oct 30;10:355. doi: 10.1038/s41392-025-02450-1 (PMC12572652; doi:10.1038/s41392-025-02450-1)
Supplement: Supplementary file 1 — Supplementary_Materials [file 41392_2025_2450_MOESM1_ESM.docx]

Supplementary Materials for

Ambient Light Alleviates Retinal Neurodegeneration in Mice by Powering Mitochondria via an Engineered Optoenergetic Rhodopsin

Run-Zhou Yang^1#^, Yiting Wang^2#^, Zhuanbi­­n Wu^3^, Yun Luo^4^, Dian-Dian Wang^1,5^, Yun Zou^2^, Youzhi Liang^2^, Jia-Kang Li^1,7^, Su Zhang^6^, Chun-Ping Huang^7^, Wei-Rong Zeng^7^, Si-Yuan Chang^4^, Sen-Miao Li^1,5^, Xiao-Yan Meng^1,5^, Hui-Fang Sun^1,5^, Pei-Pei Liu^1^, Jinzhi Lei^8^, Yang Xiang^7^, Yu Gu^2^, Biao Yan^2^, Shi-Qing Cai^4^, Jiayi Zhang^2*^, Jian-Sheng Kang^1,5*^

Correspondences: kjs@zzu.edu.cn and jiayizhang@fudan.edu.cn

**This PDF file includes:**

Supplementary Text

Materials and Methods

Figures. S1 to S9

Tables S1

Supplementary Text

Estimating the resting proton motive force (*pmf*)

At the cellular level, the rate of $O_{2}^{-}$ is

$\frac{d\left[ O_{2}^{-} \right]}{dt}=k^{+}\bullet[O_{2}]-k^{-}\bullet[O_{2}^{-}]$ (A)

where $k^{+}$ represents the generation rate of $O_{2}^{-}$, $k^{-}$ represents the clear rate of $O_{2}^{-}$*, n_0_* represents the initial value of $O_{2}^{-}$. Without loss of generality, we assume that

$\left[ O_{2} \right]+\left[ O_{2}^{-} \right]=1$ (B)

Then, the equation (A) yields

$\frac{d\left[ O_{2}^{-} \right]}{dt}=k^{+}-({k^{+}+k}^{-})\bullet[O_{2}^{-}]$ (C)

Since about 0.4-4% of oxygen could be converted into the free radical superoxide ($O_{2}^{-}$) in normal oxidative phosphorylation ^1^, it was reasonable to assume that the resting pmf (*pmf_0_*) balanced with the intracellular antioxidative system, and that the ROS level could be below the threshold of activating the antioxidative system when the pmf was less negative than the *pmf_0_*. For simplicity, we could use the step function to simulate $k^{-}$, so that $k^{-}$ = 0 when |$pmf| \leq{|pmf}_{0}|$. Then, the integration of equation (c) gives the following:

$\left[ O_{2}^{-} \right]=n_{0}\bullet e^{-k^{+}t}+1-e^{-k^{+}t}$ (D)

where *n_0_* represents an initial value of [$O_{2}^{-}$]; *t* is the time. Since most ROS are short-lived (~milliseconds) and their intracellular levels are low (~picomolar) ^2^, *n_0_* is taken to be zero. Interestingly, the transient nature and high reactivity of ROS suggest that their cumulative signaling effects and associated nuclear DNA oxidative damage in the nucleus (**Fig. 3**) may involve quantum tunneling mechanisms. The nuclear accumulation of ROS, such as H₂O₂, is primarily governed by classical diffusion processes due to its small molecular size and neutral charge ^3^. Conversely, localized quantum tunneling effects may contribute to critical steps of DNA oxidative damage ^4^.

When considering a brief timescale on the millisecond scale, the Taylor expansion of the equation (D) can be approximated as

$\left[ O_{2}^{-} \right]\cong k^{+}\bullet t$ (E)

The speed of generating $O_{2}^{-}$ is proportional to *pmf* based on the Ohm’s law, so that $k^{+}$ $\propto|pmf|$. In experiments, we used normalized 8-oxoguanine (oxG) and DNA double-stranded break values to represent DNA oxidative damage (**Fig. 3**). The mode is illustrated below.

Model 1. Schematic diagram of the generation of 8-oxoguanine

The DNA oxidative damage (8-oxoguanine) is mainly repaired through the base excision repair pathway involving multiple steps in eukaryotes, such as the recruitment of UV-damaged DNA binding protein DDB2 ^5^, the DNA repair glycosylases 8-oxoguanine glycosylase (OGG1), MUTYH and Nudix Hydrolase 1 (NUDT1) ^6^, so that the repair of 8-oxoguanine is a slower process compared to the almost instant DNA oxidative damage. Therefore, for the sake of simplicity, we assume that the repair of DNA oxidative damage is negligible when |$pmf| \leq{|pmf}_{0}|$. The total number of guanines is an invariant (~10^9^ for the human genome):

$p_{g}+p_{oxG}=1$ (F)

where $p_{g}$ represents $\frac{g}{G_{0}}$; $p_{oxG}$ represents $\frac{oxG}{G_{0}}$; *G_0_* represents the total number of guanines in the genome.

Consider the equation

$\frac{d_{p_{oxG}}}{dt}=\left( 1-p_{oxG} \right)\bullet k\bullet\left| pmf \right|\bullet t, p_{oxG}(0)= p_{0}$ (G)

which is valid in a short time (milliseconds), and where *k* is a positive scaling factor. We solve the above equation to have

${1-p}_{oxG}\left( t \right)=\left( 1-p_{0} \right)e^{- \frac{k\bullet\left| pmf \right|\bullet t^{2}}{2}}$ (H)

which gives

$p_{oxG}\left( t \right)=1-\left( 1-p_{0} \right)e^{- \frac{k\bullet\left| pmf \right|\bullet t^{2}}{2}}= {p_{0}e}^{- \frac{k\bullet\left| pmf \right|\bullet t^{2}}{2}}+1-e^{- \frac{k\bullet\left| pmf \right|\bullet t^{2}}{2}}$ (I)

Let

$p= 1-e^{- \frac{k\bullet\left| pmf \right|\bullet t^{2}}{2}}=p_{oxG}-p_{0}e^{- \frac{k\bullet\left| pmf \right|\bullet t^{2}}{2}} , |pmf| \leq|{pmf}_{0}|$ (J)

where *p* represents a measurable and normalized value of oxG (**Fig. 3q**). The equation (J) suggests that the 8-oxoguanine might amplify the ROS signal and lead to more evident DNA double-stranded breaks (**Fig. 3r, s**). In addition, *p* also represents a probability of the oxG generation when |$pmf| \leq{|pmf}_{0}|$, the equation (J) suggests that |*pmf*| is inversely proportional to the variances of the oxG results. Consistently, the standard deviation of the oxG results under the *pmf_0_* without photo-stimulation is smaller than the standard deviation of the results under photo-stimulation (**Fig. 3q**), which consistently suggests that the *pmf_0_* of mitochondria in COS-7 cells might be slightly more hyperpolarized than -216 mV. Conversely, the experimental phenomena support that the mathematical modeling is rational.

Normalizing the *pmf* in the equation (J) with the less hyperpolarized *pmf_on_* under photo-stimulation (**Fig. 3q**), we have

${|pmf}_{0}|=|{pmf}_{on}|\bullet\frac{\ln\left( 1-p \right)}{\ln\left( 1-p_{on} \right)}$ (K)

The real *p* values are usually quite small (~10^-6^ for the human genome) ^7^, so that the Taylor expansion of the equation (H) gives

${|pmf}_{0}|=|{pmf}_{on}|\bullet\frac{p}{p_{on}}$ (L)

where the *p/p_on_* is invariant under *p* scaling, such as a scale of *p* to 1 (**Fig. 3q**).

Consequently, the *pmf_0_* was estimated around -222 mV with the equation (L) using the experimental mean values of the normalized *p* values (**Fig. 3q**). Since the 95% confidence interval for the reversal potential of mt-EcGAPR was between -250 mV and -190 mV (**Supplementary Table 1**), consequently, the linear characteristic of the equation (L) suggested that the 95% confidence interval for the *pmf_0_* was between -195 mV and -257 mV, which overlapped substantially with the range (-210 mV to -270 mV) reported by Mitchell and Moyle ^8^. Overall, the analysis tells us that the resting proton motive force of mitochondrion is slightly more negative than -216 mV (the reversal potential of EcGAPR, **Fig. 2k and Supplementary Table 1**), consequently, the range of the *pmf_0_* is most likely from -222 mV to -257 mV (the upper half of the above 95% confidence interval).

Materials and Methods

**Materials**

**Key resources table:**

| **Reagent or Resource** | **Source** | **Identifier** |
| --- | --- | --- |
| Antibodies | | |
| Goat anti-rabbit IgG H&L (HRP) | Abcam | Cat# ab6721; RRID: AB_955447 |
| Goat anti-mouse IgG (H+L) HRP | Sharebio | Cat# SB-AB0102; RRID: N/A |
| Goat anti-Brn3a | Santa Cruz Biotechnology | Cat# SC-31984; RRID: AB_2167511 |
| Chicken anti-GFP | Aves Labs, Inc. | Cat# GFP-1020; RRID: AB_10000240 |
| Rabbit anti-PKC-alpha | Abcam | Cat# ab32376; RRID: AB_777294 |
| Donkey anti-Chicken conjugated to Alexa Flour 488 | Jackson ImmunoResearch | Cat# 703-545-155; RRID: AB_2340375 |
| Donkey anti-Mouse conjugated to Alexa Flour 647 | Jackson ImmunoResearch | Cat# 715-605-150; RRID: AB_2340862 |
| Donkey anti-Goat conjugated to Alexa Flour 594 | Jackson ImmunoResearch | Cat# 705-585-003; RRID: AB_2340432 |
| Moue anti-His-tag | Abcam | Cat# ab18184; RRID: AB_444306 |
| Mouse anti-TOM20 | Santa Cruz | Cat# sc-17764; RRID: AB_ 628381 |
| Mouse anti-Hsp60 | Enzo | Cat# ADI-SPA-806-F; RRID: AB_ 11177888 |
| Mouse anti-GAPDH | Abcam | Cat# ab9484; RRID: AB_ 307274 |
| Mouse anti-gamma-H2AX | Cell Signaling Techonology | Cat# 80312; RRID: AB_2799949 |
| Mouse anti-8-oxoguanine | Sigma | Cat# MAB3560; RRID: AB_ 94925 |
| Mouse anti-Myc-tag | Proteintech | Cat# 60003-2-Ig; RRID: AB_2734122 |
| Rabbit anti-Myc-tag | Proteintech | Cat# 16286-1-AP; RRID:AB_11182162 |
| Rabbit anti-mitofilin | Proteintech | Cat# 10179-1-AP; RRID: AB_2127193 |
| Goat anti-mouse Alexa Fluor 555 | Thermo | Cat# A-21422; RRID: AB_2535844 |
| Goat anti-Rabbit Alexa Fluor 633 | Thermo | Cat# A-21070; RRID: AB_2535731 |
| Mouse anti-TOMM20 | Sigma | Cat# WH0009804M1; RRID:AB_1843992 |
| Rabbit anti-BIP | Proteintech | Cat# 11587-1-AP; RRID:AB_2119855 |
| Rabbit anti-PERK | Proteintech | Cat# 24390-1-AP, RRID:AB_2879521 |
| Mouse anti-β-actin | Sharebio | Cat# SB-AB2001 |
| Rabbit anti-ATF6 | Proteintech | Cat# 24169-1-AP;RRID:AB_2876891 |
| Rabbit anti-GSDMD | Abcam | Cat# 219800;  RRID:AB_2888940 |
| Rabbit anti-IL 18 | Abcam | Cat# ab207323;  RRID:AB_2895063 |
| Rabbit anti-IL1 β | Immnunoway | Cat# YM4682; RRID:AB_3662998 |
| Bacterial and virus strains | | |
| AAV virus (DJ serotype): EF1α-mtecGAPR-EGFP | This paper | N/A |
| Lenti virus: CMV-mtecGAPR-EGFP | This paper | N/A |
| E. coli: Strain OP50 | SQC lab | N/A |
| E. coli: Strain BL21(DE3) | Cwbio | Cat# CW0809 |
| E. coli: Strain DH5α | Cwbio | Cat# CW0808 |
| E. coli: Strain Stbl3 | TransGen Biothech | Cat# CD521-01 |
| Chemicals, peptides, and recombinant proteins | | |
| Avertin | Nanjing Aibei Biotechnology Co., Ltd | N/A |
| RIPA buffer | Thermo Scientific | 89900 |
| Arciolane 1300 Syringe (silicone oil) | Arcadophta Sar | Arciolane 1300 |
| NaCl | Sigma | [S5886](https://www.sigmaaldrich.cn/CN/zh/product/sigma/s5886) |
| NaHCO_3_ | Sigma | S6014 |
| KCl | Sigma | [P5405](https://www.sigmaaldrich.cn/CN/zh/product/sigma/p5405) |
| KH_2_PO_4_ | Sigma | P0662 |
| CaCl_2_ | Sigma | C5670 |
| MgSO_4_ | Sigma | M7506 |
| HEPES | Sigma | H4034 |
| NaOH | Sigma | 655104 |
| Glucose | Sigma | G7021 |
| kanamycin | Sangon | A100408 |
| Ampicillin | Sangon | A100339 |
| Sucrose | Sigma | S9378 |
| EGTA | Sigma | E4378 |
| CCCP | Sigma | C2759 |
| Phosphatase inhibitor cocktail | Roche | 4906845001 |
| Protease inhibitor cocktail | Roche | 04693116001 |
| SuperSignal West Atto | Thermo Scientific | A38554 |
| AQUA-MOUNT | Thermo Scientific, USA | N/A |
| Minimal Essential Medium | Gibco | 51200038 |
| Bovine transferrin | Calbiochem | 616420 |
| Cytosine-p-arabinofuranoside | Sigma | C1768 |
| B27 medium supplement | Invitrogen | A1895601 |
| Collagenase II | Sigma | C6885 |
| Geneticin | Gibco | 345810 |
| MitoSOX | Invitrogen | M36005 |
| SNARF-1-AM | Invitrogen | C-1271 |
| Rotenone | Sigma | R8875 |
| MS-222 | Sigma | E10521 |
| All-trans retinal | Sigma | R2500 |
| Trypsin XI | Sigma | T1005 |
| DNase type IV | Sigma | D5025 |
| Cytosine-p-arabinofuranoside | Sigma | C6645 |
| Matrigel | Corning | 356234 |
| Puromycin | Sangon | A610593 |
| Insulin | Sigma | I5500 |
| Ficoll 400 | Sigma | F8016 |
| ATP | Sigma | FLAAS |
| glutathione | Sangon | A600229 |
| QuickShuttle Enhanced | Biodragon | KX0110042 |
| QuickShuttle Basic | Biodragon | KX0110041 |
| sodium azide | Sigma | S2002 |
| Dulbecco’s modified Eagle medium (high glucose) | Gibco | 11995081 |
| Dulbecco’s modified Eagle medium (low glucose) | Gibco | 11885084 |
| Trypan blue | Sangon | A601140 |
| β-carotene | Cayman | 16837 |
| OCT compound | Sakura Finetek, USA | 4583 |
| paraquat | Sigma | 36541 |
| Acridine Orange hemi(zinc chloride) salt | Sigma | A6014 |
| IPTG | sangon | A600168 |
| Nco I | NEB | R0193 |
| SalI | NEB | R3138 |
| BamHI | NEB | R3136 |
| Nhe I | NEB | R3131 |
| Not I | NEB | R3189 |
| Kpn I | NEB | R3142 |
| Xba I | NEB | R0145 |
| Triton X100 | sangon | A110694 |
| Chenodeoxycholic acid | Sigma | C9377 |
| Digitonin | Sigma | D141 |
| Dihydroethidium (DHE) | Beyotime | S0063 |
| Levofloxacin hydrochloride | Santan, Japan | NA |
| Paraformaldehyde | Beyotime | P0099 |
| DAPI | Sigma | D9542 |
| mito-TEMPO | Sigma | SML0737 |
| Critical commercial assays | | |
| BCA protein assay kit | Thermo Scientific | 23225 |
| Membrane and Cytosol Protein Extraction Kit | Beyotime | P0033 |
| ATP Assay Kit | Beyotime | S0026 |
| ATP Assay Kit | Beyotime | S0027 |
| Succinate Assay Kit | Abcam | ab204718 |
| NAD/NADH Assay Kit | Abcam | ab65348 |
| Mito Stress Test Kit | Agilent Technology | 103015-100 |
| Experimental models: Cell lines | | |
| HEK 293t | ATCC | Cat# CRL-3216; CVCL_0063 |
| COS-7 | ATCC | Cat# CRL-1651; CVCL_0224 |
| Hela | ATCC | Cat# CCL-2; RRID: CVCL_0030 |
| Experimental models: Organisms/strains | | |
| Mouse: Strain C57 BL/6 | Shanghai JieSiJie Laboratory Animal Co., Ltd. | RRID: IMSR_JAX:000664 |
| Mouse: Strain R26-e (CAG- mt-EcGAPR -2A-EGFP) | Shanghai Model Organisms Center, Inc. | N/A |
| Zebrafish: Strain TG (zlyz:EGFP) | Shanghai Model Organisms Center, Inc. | N/A |
| *C. elegans*: Strain wild-type Bristol N2 | Caenorhabditis Genetics Center | N/A |
| Recombinant DNA | | |
| Bacteriorhodopsin (BR) (humanized codon) | This paper | GenBank Id: P02945 |
| detarhodopsin (HtdR) (humanized codon) | This paper | GenBank Id: O93740 |
| xanthorhodopsin (AXR) (humanized codon) | This paper | GenBank Id: WP_007675008 |
| Gloeobacter rhodopsin (GR) (humanized codon) | This paper | GenBank Id: WP_011140202 |
| Leptosphaeria rhodopsin (MAC) (humanized codon) | This paper | GenBank Id: AAG01180.1 |
| Alpha proteorhodopsin (APR) (humanized codon) | This paper | GenBank Id: WP_014952819 |
| Archaerhodopsin T (ArchT) | Addgene | Cat# 31177; RRID: Addgene_31177 |
| Green absorbing proteorhodopsin (GPR) | Addgene | Cat# 33780; RRID: Addgene_33780 |
| Coccomyxa rhodopsin (CsR) | Arend Vogt | GenBank Id: I0YUS5 |
| pCDH-CMV-puromycin | JSK lab | N/A |
| pEGFPN1 | JSK lab | N/A |
| pcDNA3.1-mt-EcGAPR | This paper | N/A |
| pcDNA3.1-mt-EcGAPR(D97N) | This paper | N/A |
| pet28c(+) | JSK lab | N/A |
| ppd95.75 | SQC lab | N/A |
| *pdat-1::mt-EcGAPR::GFP* | This paper | N/A |
| *pdat-1::Tomm20::mCherry* | This paper | N/A |
| *pdat-1:: 4cox8::GFP* | This paper | N/A |
| *pdat-1::mCherry* | This paper | N/A |
| GPD-EYFP | This paper | N/A |
| GPD-ECFP | This paper | N/A |
| Software and algorithms | | |
| ImageJ | NIH | https://imagej.net/ij/download.html; RRID:SCR_003070 |
| NIS-Elements D4.6 | Nikon | https://www.nikoninstruments.com/Products/Software; RRID:SCR_014329 |
| Adobe Photoshop 7.0 | Adobe | http://www.adobe.com/products/photoshop.html; RRID:SCR_014199 |
| Adobe Illustrator 2021 | Adobe | http://www.adobe.com/products/illustrator.html; RRID:SCR_010279 |
| pClamp | Molecular Devices | http://www.moleculardevices.com/products/software/pclamp.html; RRID:SCR_011323 |
| R Project for Statistical Computing | Free software | https://www.r-project.org/; RRID:SCR_001905 |
| Perl Programming Language | Free software | https://www.perl.org/; RRID:SCR_018313 |
| Python Programming Language | Free software | <http://www.python.org/>; RRID:SCR_008394 |
| MATLAB | Mathworks | RRID:SCR_001622 |
| Seahorse Wave | Agilent | RRID:SCR_014526 |
| Excel | Microsoft | RRID:SCR_016137 |
| Other | | |
| TonoLab tonometer | Icare | Tonolab |
| temperature controller | Warner | N/A |
| Ultrasonic cell disruptor | BMH, Germany | N/A |
| Laser scanning confocal microscope | Olympus | FV1000 |
| Micro-Combination pH Needle Electrode | Microelectrodes, Inc | N/A |
| Laser scanning confocal microscope | Olympus | FV3000 |
| Multi-photon scanning microscope | Nikon Inc, Japan | AIR-MP |
| Freezing microtome | Leica, Germany | CM 3050S |
| Freezing microtome | Leica, Germany | CM 1950 |
| Fluorescence microscope | Olympus | IX83 |
| Fluorescence microscope | Nikon | SMZ18 |
| NanoJect II | Drummond Scientific Company, USA | 3-000-205A |
| Glass pipette (for injection) | Drummond Scientific Company, USA | 3.5" Drummond # 3-000-203-G/X |
| Filamented glass capillaries (for patch clamp) | Sutter Instrument | BF150-86-10 |
| Programable micropipette puller | Sutter Instruments, USA | P2000 |
| Stereo microscope | ZIESS | Stemi 508 |
| Electroporation apparatus | BTX, USA | ECM830 |
| DigiData | Molecular Devices | 1440A |
| Axopatch amplifier | Molecular Devices | 700B |
| Monochromator | Cairn Research Ltd., UK | Optoscan |
| Filamented glass capillaries | Sutter Instrument, | BF150-86-10 |
| Micromanipulator | Sutter | MP285 |
| Seahorse Extracellular Flux Analyzer | Agilent Technology | XF24 |
| Optical coherence tomography | Mocean | 3000Plus |
| E-blot Western blot imaging system | Yibote Life Science | Touch Imager |
| Ultrasonic Homogenizer | COLE-PARMER INSTRUMENTS | CPX 750 |

**Microbes**

*Escherichia coli* DH 5α, BL21(DE3), STBL3, and OP50 cells were cultured in LB medium.

**Cell culture**

HEK293t, HeLa, and COS-7 cells were cultured in DMEM Medium (GIBCO) supplemented with 10% FBS in an incubator at 37 ℃ and 5% CO_2_.

**Mice**

All animal care and experiments were performed by the Shanghai Medical College Institutional Animal Care of Fudan University and the Institutional Animal Care and Use Committee of Zhengzhou University guidelines. Wild-type (C57BL/6J) mice were obtained from the Shanghai JieSiJie Laboratory Animal Co., Ltd. (Shanghai, China). Mt-EcGAPR mice were generated by Shanghai Model Organisms Center, Inc. (Shanghai, China). 9 -12 weeks C57BL/6J mice (male) and mt-EcGAPR mice (male) were used in the experimental glaucomatous experiments. All mice were maintained in a 12-hour light-dark cycle at 18-22 ℃. All experiments were conducted during the light cycle. Growing evidence indicates a protective effect of estrogen in glaucoma^9^. In our previous studies using female mice, we observed substantial variability in the experimental outcomes. To minimize variability caused by hormonal fluctuations during the estrous cycle, only male mice were included in this study.

**Zebrafish**

Adult zebrafish were maintained at 28.5 ℃ on a 14 h light/10 h dark cycle.

**References**

1. Evans, J. L. *et al*. Oxidative Stress and Stress-Activated Signaling Pathways: A Unifying Hypothesis of Type 2 Diabetes. *Endocr. Rev.* **23**, 599–622 (2002).
2. Murphy, M. P. *et al*. Guidelines for measuring reactive oxygen species and oxidative damage in cells and in vivo. *Nat. Metab.* **4**, 651–662 (2022).
3. Malinouski, M., Zhou, Y., Belousov, V. V., Hatfield, D. L. & Gladyshev, V. N. Hydrogen Peroxide Probes Directed to Different Cellular Compartments. *PLOS ONE* **6**, e14564 (2011).
4. Slocombe, L., Sacchi, M. & Al-Khalili, J. An open quantum systems approach to proton tunnelling in DNA. *Commun. Phys.* **5**, 1–9 (2022).
5. Kumar, N. *et al*. Global and transcription-coupled repair of 8-oxoG is initiated by nucleotide excision repair proteins. *Nat. Commun.* **13**, 974 (2022).
6. Banda, D. M. *et al*. Repair of 8-oxoG:A Mismatches by the MUTYH Glycosylase: Mechanism, Metals & Medicine. *Free Radic. Biol. Med.* **107**, 202–215 (2017).
7. Ohno, M. *et al*. A genome-wide distribution of 8-oxoguanine correlates with the preferred regions for recombination and single nucleotide polymorphism in the human genome. *Genome Res.* **16**, 567–575 (2006).
8. Mitchell, P. & Moyle, J. Estimation of membrane potential and pH difference across the cristae membrane of rat liver mitochondria. *Eur. J. Biochem.* **7**, 471–484 (1969).
9. Zhao, S. H. *et al*. Comparative insights into the role of sex hormones in glaucoma among women and men. *Prog. Retin. Eye Res.* **105**, 101336 (2025).

Figure. S1.


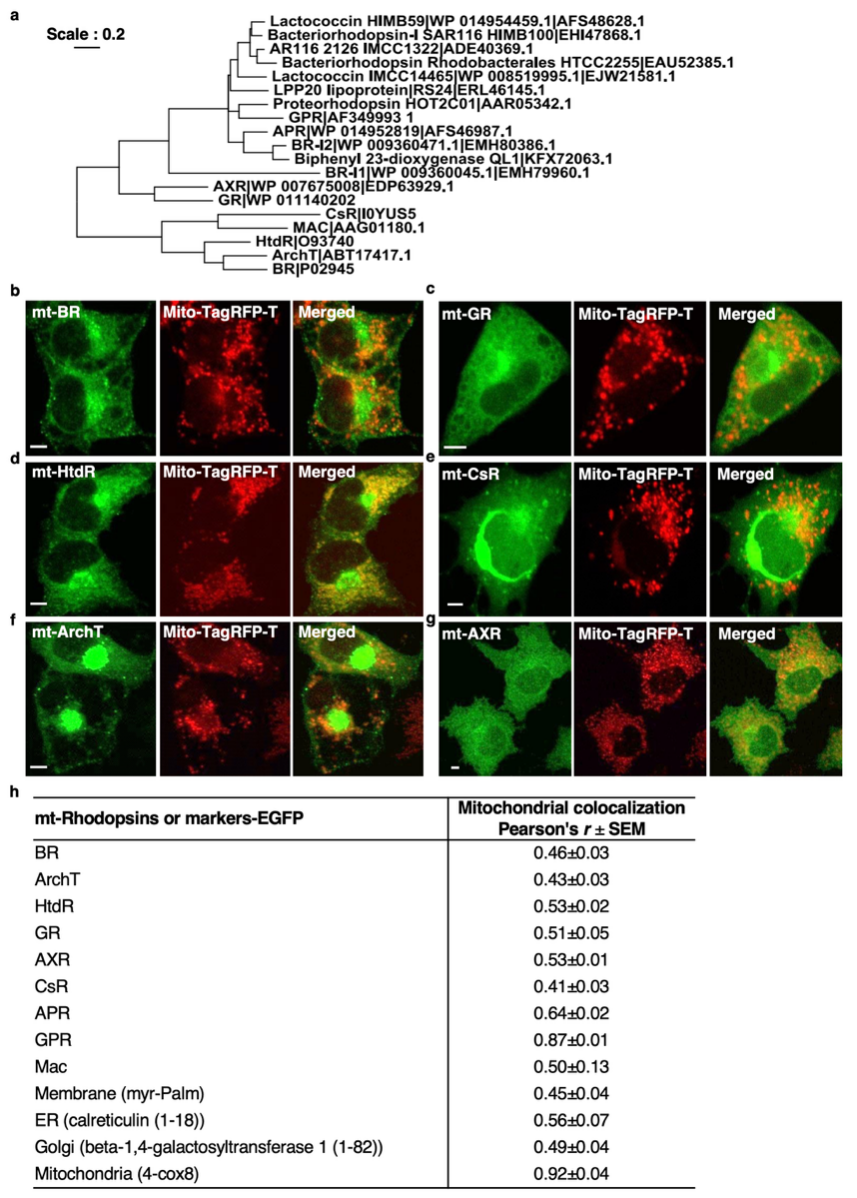


## Figure S1. Confocal imaging and analysis of PPR’s mitochondrial targeting efficiency

(a) Phylogeny analysis of microbial rhodopsin candidates.

(b-g) Representative confocal images of COS7 cells expressing mt (4COX8)-fused rhodopsins, including mt-BR (b), mt-GR (c), mt-HtdR (d), mt-CsR (e), mt-ArchT (f), and mt-AXR (g). Mito-tagRFP-T was used as a mitochondrial marker. Scale bars, 5 μm.

(h) Pearson's correlation coefficients of mt-fused rhodopsins and subcellular markers. Pearson's correlation coefficient (Pearson's r) was calculated to assess the extent of colocalization between the mt-fused rhodopsins and mitochondria. The mean ± SEM values of Pearson's r for each rhodopsin were listed. To provide a comparative analysis, the localization of ER, Golgi, plasma membrane, and mitochondrial markers was also evaluated. APR exhibited a partial colocalization with mitochondria, as indicated by its r value of 0.64. In contrast, GPR and the mitochondrial marker demonstrated a higher degree of colocalization with mitochondria, as evident from their larger r values. Rhodopsins with an r value below 0.6 exhibited targeting to the ER or Golgi rather than mitochondria.

Figure. S2.


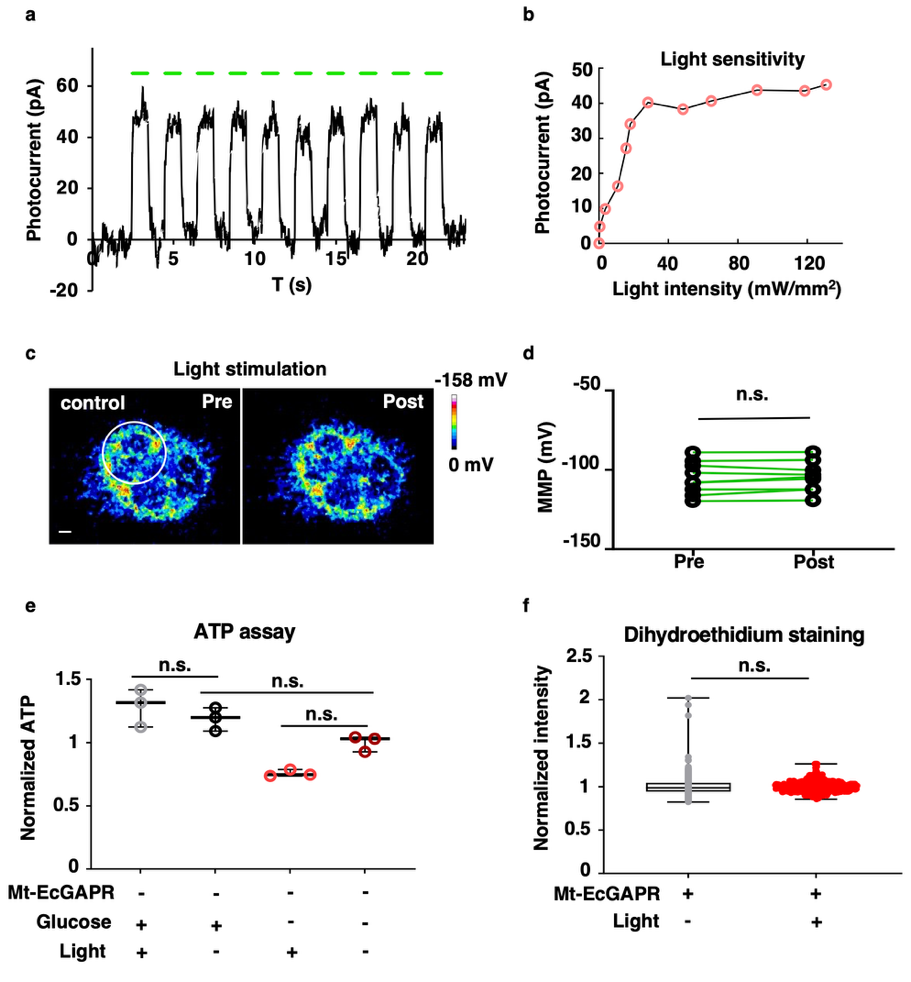


## Figure. S2. Characterization of EcGAPR and mt-EcGAPR in mammalian cells, related to Figures 2 and 3

(a) A representative trace of photocurrents of EcGAPR upon light-stimulation recorded in HEK293t cells.

(b) Light intensity of EcGAPR (n = 4 cells).

(c) Representative confocal images of control COS7 cell stained with Rhodamine 800 before and after light stimulation. The white circle indicated the region of light stimulation. Scale bar, 5 μm.

(d) Quantification of mitochondrial membrane potential before and after light stimulation (paired *t-test*, p > 0.05, ns).

(e) ATP levels of COS7 cells lacking mt-EcGAPR upon light stimulation. The ATP levels were evaluated in COS7 cells cultured in either high or low glucose medium, under dark or light illumination (green, 37.18 mW mm^-2^) (N = 3 replicates; no significant, n.s., *t-test*).

(f) Absence of intracellular ROS generation by mt-EcGAPR upon light stimulation indicated by dihydroethidium staining. Intracellular reactive oxygen species (ROS) levels were visualized using dihydroethidium in a COS7 cell line stably transfected with mt-EcGAPR, following light illumination (green, 37.18 mW mm^-2^). A total of 373 cells were analyzed in dark conditions, while 646 cells were examined under light stimulation (no significant, n.s., *t-test*).

The data are represented as the means ± SEMs.

Figure. S3.


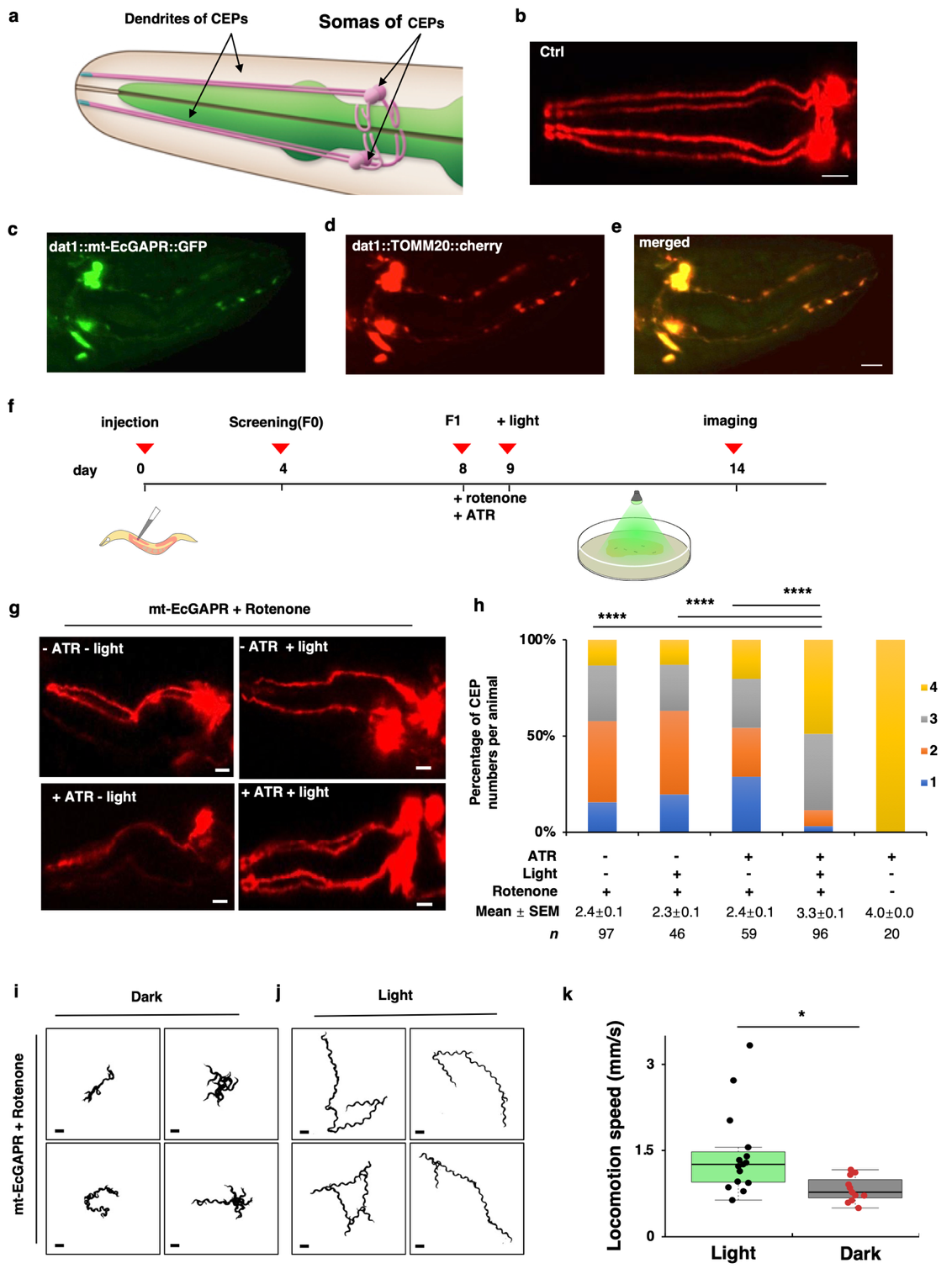


## Figure. S3. Protective effect of mt-EcGAPR in C. elegans Parkinson's disease model under light-stimulation.

(a) A diagram of cephalic dopaminergic neurons (CEPs) of *C. elegans* adapted from Wormatlas website.

(b) Fluorescence image of normal worms with intact CEPs. Scale bar, 10 μm.

(c-e) Mitochondrial targeting of mt-EcGAPR-GFP in cephalic dopaminergic neurons (CEPs). *C.elegans* was co-injected with *dat1::EcGAPR::GFP* (green) and *dat1::TOMM20::mcherry* (red) for colocalization analysis. Scale bar, 10 μm.

(f) Timeline for plasmid injection, rotenone treatment, and light stimulation in *C. elegans*. Plasmids containing *dat1::mt-EcGAPR::GFP* and *dat1::mCherry* were co-injected into the gonad of *C. elegans*. The injected worms (F0) were screened for positive expression and allowed to grow until the F1 generation. Positive expression F1 worms were selected and transferred to NGM plates containing 4 μM rotenone. The worms were fed with *E. coli* OP50 supplemented with all-trans retinal (ATR, 500 μM). Light illumination (green, 10000 lux, 1 Hz) was applied for 5 days in an incubator set at 20℃. Subsequently, the worms were subjected to fluorescence imaging and behavioral analysis.

(g) Fluorescence images of CEPs in *C. elegans* under four different experimental conditions: grown on NGM with rotenone (i) without ATR and without light illumination, (ii) without ATR supplementation and with light illumination, (iii) with ATR and without light illumination, and (iv) with ATR supplementation and with light illumination. Scale bar, 10 μm.

(h) Percentage stacked bar chart of CEP numbers of worms expressing mt-EcGAPR under different experimental conditions. Worms with ATR supplementation and light illumination preserved most CEP neurons compared to other groups (*p* < 0.0001, *t-test*).

(i-j) Photo-stimulation improves rotenone-induced locomotion deficiency in *C. elegans* with mt-EcGAPR overexpressed in dopamine neurons. Worms grown on NGM containing rotenone with or without light illumination were analyzed for locomotion activity. In behavior assay, single worm was picked on a new NGM plate and imaged for 2 min. The traces of worm locomotion with (J, n = 15) or without (I, n = 11) light illumination was shown (*p* = 0.015, *t-test*). Scale bars, 1 mm.

(k) The scatter and box plot of locomotion speed of worm with or without light illumination. The speed was significantly increased with light illumination (*p* = 0.0147, *, *t-test*).

The data are represented as the means ± SEMs.

Figure. S4.


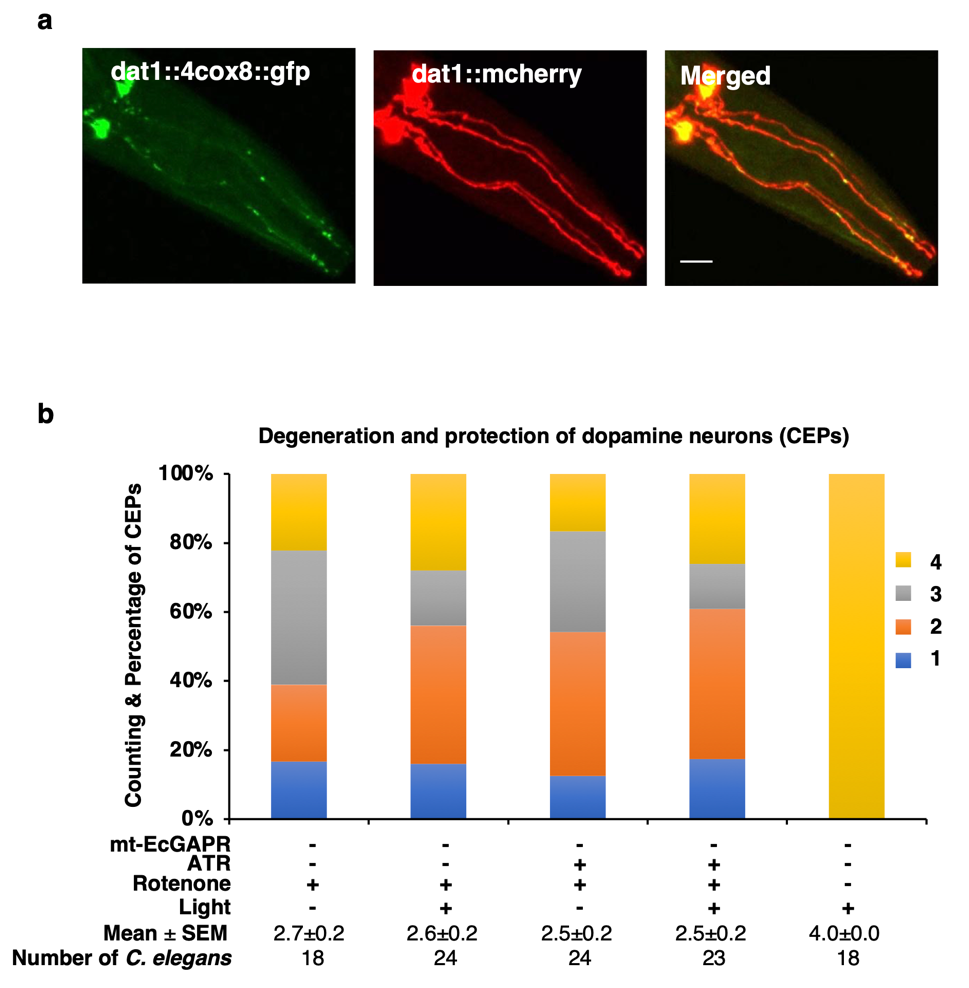


## Figure. S4. Rotenone-induced neuronal degeneration in C.elegans without mt-EcGAPR

(a) Fluorescence images of *C.elegans* injected with *dat1:4cox8::GFP/dat1::mcherry*. Scale bar, 10 μm.

(b) Percentage stacked bar chart of CEP numbers of worms expressing *dat1:4cox8::GFP/dat1::mcherry* under different experimental conditions. In worms lacking mt-EcGAPR, supplementation with ATR combined with light illumination did not prevent the rotenone-induced loss of dopaminergic neurons (n.s. not significant, *t-test*).

Figure. S5.


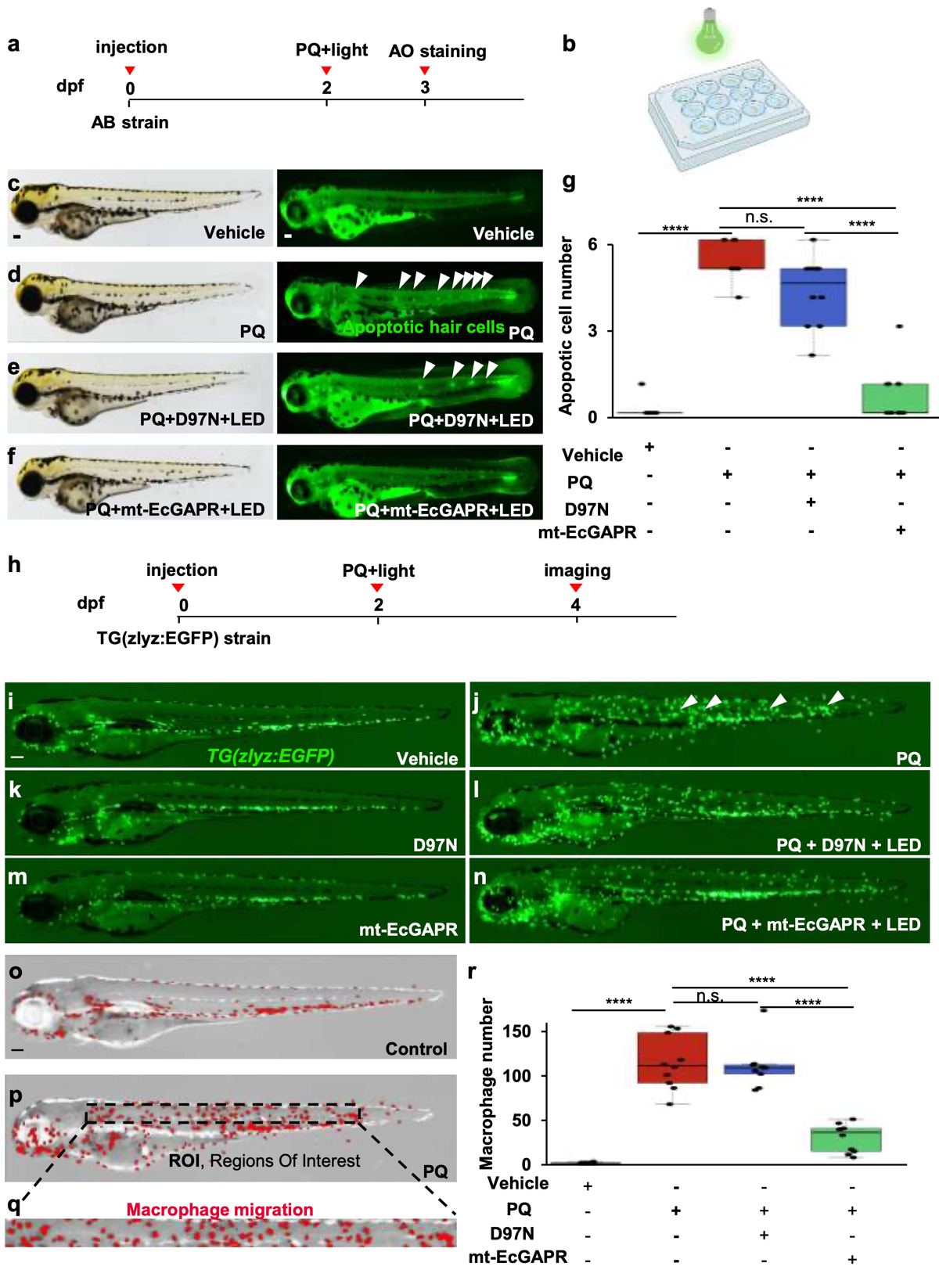


## Figure. S5. Photo-stimulation protects mt-EcGAPR transgenic zebrafish against paraquat-induced cell death

(a) Timeline of PQ-induced apoptosis assay. Zebrafish (AB strain) embryos were injected with mt-EcGAPR or mt-EcGAPR(D97N). At 2 pdf, embryos were subjected to paraquat treatment and light stimulation. The 3dpf embryos were stained with AO for apoptotic cell staining.

(b) The light stimulation setup. Green LED (540 nm, 10,000 lux, 900 ms ON, 100 ms OFF) was illuminated upon zebrafish cultured in 12-well plate in an incubator set at 28.5℃.

(c-f) Bright field and fluorescence images of zebrafish larvae at 3 dpf upon different treatments (lateral view, anterior, left). Zebrafish larvae following PQ and light treatments were stained with acridine orange (AO). Apoptotic cells were indicated by bright green spots. Control zebrafish (c) exhibited few or no apoptotic cells in hair cells of the lateral line. In contrast, paraquat-treated wildtype or mt-EcGAPR(D97N) injected zebrafish showed increased staining throughout the lateral line (d, e, white arrows). Zebrafish embryos expressing mt-EcGAPR (f) demonstrated a significant inhibition of hair cell apoptosis induced by paraquat. Scale bar, 100 μm.

(g) The scatter and box plot for the apoptotic number of hair cells. The apoptotic number in mt-EcGAPR injected zebrafish was significantly lower than wildtype or mt-EcGAPR(D97N) injected zebrafish (n =10, *p* < 0.0001, *t-test*).

(h) Timeline of PQ-induced macrophage migration assay. The *TG(zlyz:EGFP)* zebrafish embryos were injection with mt-EcGAPR or mt-EcGAPR(D97N). At 2 pdf, embryos were subjected to paraquat treatment and light stimulation. The 4dpf embryos were imaged for macrophage migration.

(i-n) Representative fluorescence images of the macrophages expressed EGFP in *TG(zlyz:EGFP)* larvae at 4 days post-fertilization with different treatments. Vehicle control fish (i), D97N (k) or mt-EcGAPR fish (m) show the normal distribution of labeled cells, mostly localized in the ventral trunk and tail. In paraquat-treated siblings, macrophages become localized preferentially to a few clusters along the horizontal midline of the trunk and throughout the head (j, white arrowheads). Zebrafish embryos expressing mt-EcGAPR (n) exhibited a significant inhibition of macrophage migration induced by paraquat. Scale bar, 100 μm.

(o-r) Quantification and the scatter and box plot (r) for the macrophage number in myoseptum (ROI in p and zoomed in q). (Scale bar, 100 μm, n = 10; n.s., not significant; ****, *p* < 0.0001, *t-test*).

The data are represented as the means ± SEMs.

Figure. S6.


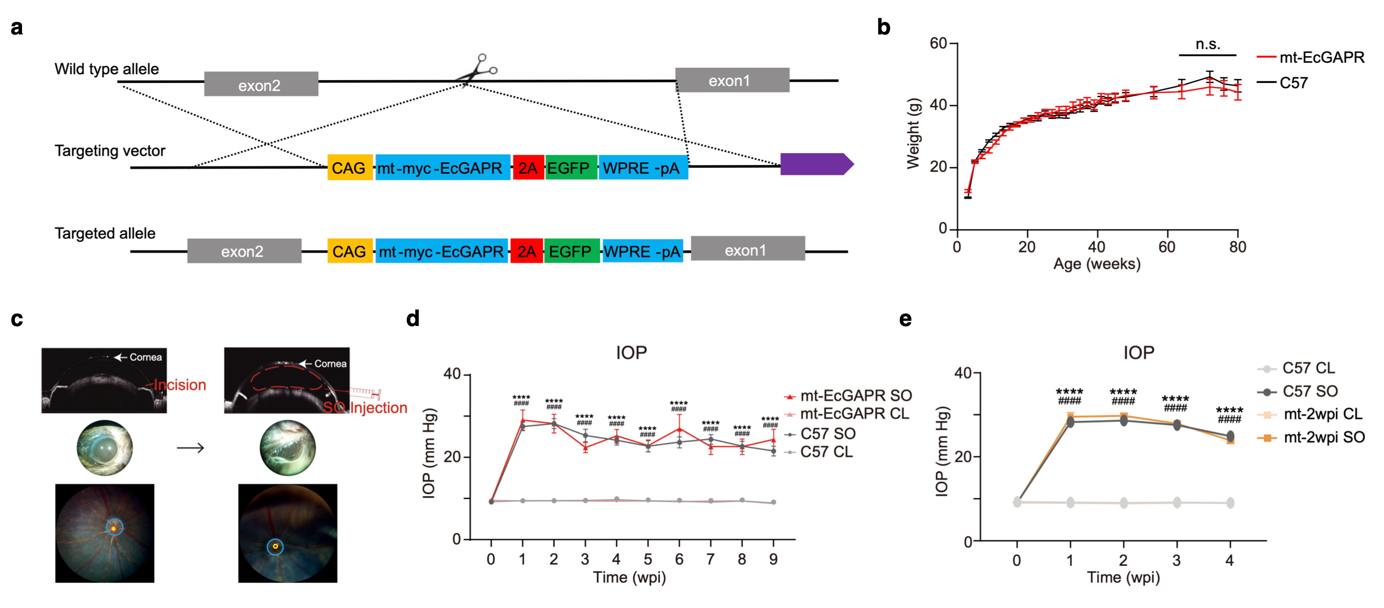


## Figure. S6. Silicone oil-induced ocular hypertension glaucoma model and intraocular pressure (IOP) analysis, related to Figures 4 and 6

(a) Illustration of generation of CAG-mt-myc-EcGAPR-2A-EGFP knock-in mice.

(b) Body weight curves of mt-EcGAPR knock-in mice and C57 BL/6J mice (n.s. not significant, *t-test*).

(c) Illustration of SO-induced ocular hypertension model. 1st row, anterior segment image before and after SO injection. 2nd row, images of eye before and after SO injection. 3rd row, fundus images of eye before and after SO injection. Yellow circle, optic cup. Blue circle, optic disk.

(d) IOP curves of C57 BL/6 mice and mt-EcGAPR mice before and after SO injection. Light gray circles, CL eyes of C57 BL/6J mice. Medium gray circles, SO eyes of C57 BL/6J mice. Pink tangles, CL eyes of mt-EcGAPR mice. Red tangles, SO eyes of mt-EcGAPR mice. N = 54, 58, 35, 44, 38, 15, 15, 15, 15 for C57 BL/6 mice and n = 40, 30, 26, 18, 18, 16, 9, 9, 9 for mt-EcGAPR mice (****, *p* < 0.0001, *t-test*).

(e) IOP curves of C57 BL/6J mice and mt-2wpi mice before and after SO injection. Light gray circles, CL eyes of C57 BL/6J mice. Medium gray circles, SO eyes of C57 BL/6J mice. Light orange rectangles, CL eyes of mt-2wpi mice. Orange rectangles, SO eyes of mt-2wpi mice. N = 15, 15, 15, 15 for C57 BL/6J mice and n = 8, 8, 8, 8 for mt-2wpi mice (****, *p* < 0.0001; *t-test*).

The data are represented as the means ± SEMs.

Figure. S7.


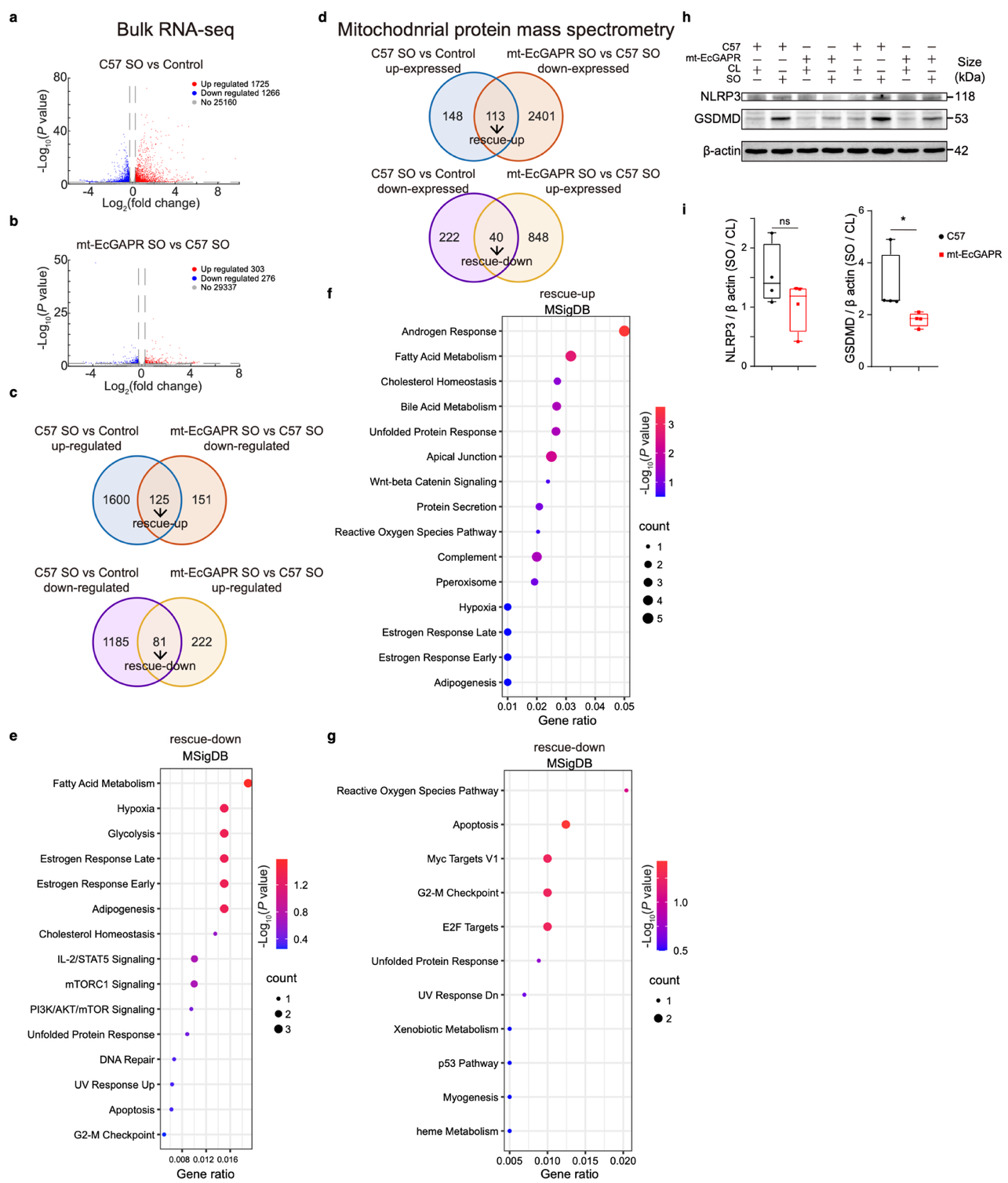


## Figure. S7. Analysis of bulk RNA-seq and mitochondrial protein mass spectrometry data, related to Figure 5

(a) Volcano plot of genes between SO eyes of C57 BL/6J mice and control C57 BL/6J mice.

(b) Volcano plot of genes between SO eyes of C57 BL/6J mice and mt-EcGAPR mice.

(c) Venn plots of rescue-up and rescue-down genes found in bulk RNA-seq experiments.

(d) Venn plots of rescue-up and rescue-down proteins found in mitochondria mass spectrum experiments.

(e) MSigDB pathways of rescue-down genes.

(f) MSigDB pathways of rescue-up proteins.

(g) MSigDB pathways of rescue-down proteins.

(h) Representative Western blots of NLRP3 and GSDMD with β-actin as the loading control.

(i) Quantification of relative protein expression for NLRP3. Black circles, C57 BL/6J mice (n = 4). Red rectangles, mt-EcGAPR mice (n = 4). The data are represented as the means ± SEMs. Statistical results:ns, not significant; *, *p* < 0.05.

Figure. S8.


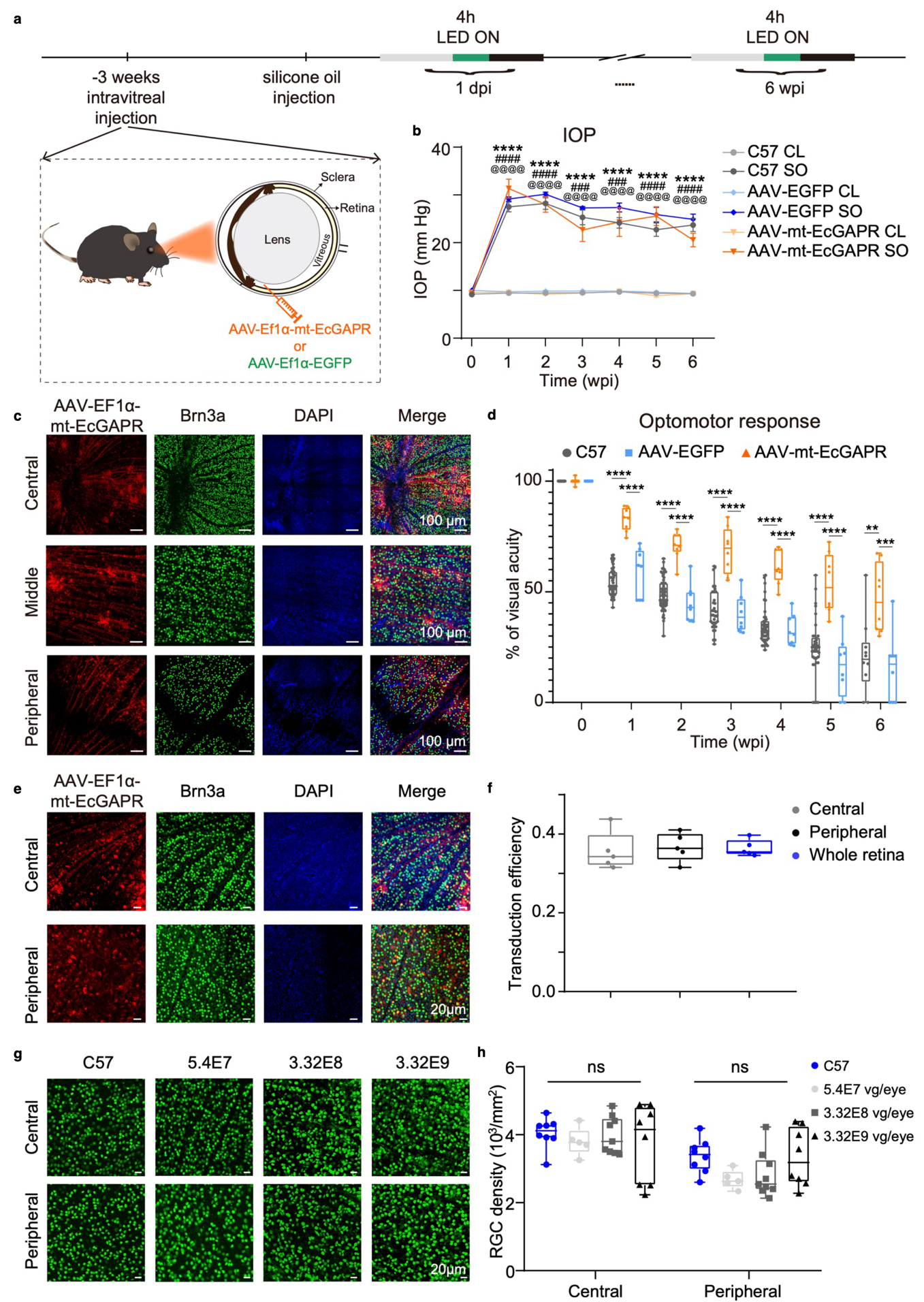


## Figure. S8. Optoenergetic activation of mt-EcGAPR could partially rescue vision in AAV-mt-EcGAPR-injected ocular hypertension mice, related to Figure 6

(a) Schematic of the AAV-mediated mt-EcGAPR delivery and optoenergetic activation experiments.

(b) IOP curves of C57 BL/6J mice, AAV-EGFP-injected mice (n = 8) and AAV-mt-EcGAPR-injected mice (n = 8) before and after SO injection. Light gray circles, CL eyes of C57 BL/6J mice. Medium gray circles, SO eyes of C57 BL/6J mice. Light blue diamonds, CL eyes of AAV-EGFP-injected mice. Blue diamonds, SO eyes of AAV-EGFP-injected mice. Light orange tangles, CL eyes of AAV-mt-EcGAPR-injected mice. Orange tangles, SO eyes of AAV-mt-EcGAPR-injected mice. N = 54, 58, 35, 44, 38, 15 for C57 BL/6 mice.

(c) Representative immunohistochemistry staining images of whole-mount retinae in AAV-EF1α-mt-EcGAPR (5.4E7 vg/eye) injected mice. Red: mt-EcGAPR immunostained with GFP antibody. Green: Brn3a, a marker for retinal ganglion cells. Blue, DAPI. Scale bar, 100 μm.

(d) Visual acuity measured by optomotor response of C57 BL/6J mice AAV-EGFP-injected mice (n = 8) and AAV-mt-EcGAPR-injected mice (n = 8) before and after SO injection. Gray circles, C57 BL/6J mice. Blue rectangles, AAV-EGFP-injected mice. Orange tangles, AAV-mt-EcGAPR-injected mice. N = 54, 58, 35, 44, 38, 10 for C57 BL/6 mice.

(e) Representative immunohistochemistry staining images of whole-mount retinae in AAV-EF1α-mt-EcGAPR (5.4E7 vg/eye) injected mice 9 weeks after injection. Green, Brn3a. Blue, DAPI. Scale bar, 20 μm.

(f) Transduction efficiency of AAV-EF1α-mt-EcGAPR (5.4E7 vg/eye) 9 weeks after injection (n = 5).

(g) Representative immunohistochemistry staining images of whole-mount retinae in mice injected with three different amount of viral genome of AAV-EF1α-mt-EcGAPR . Green, Brn3a. Scale bar, 20 μm.

(h) RGC density of C57 BL/6J mice (n = 8), AAV-EF1α-mt-EcGAPR-injected mice with 5.4E7 vg/eye (n = 5), AAV-EF1α-mt-EcGAPR-injected mice with 3.32E8 vg/eye (n = 9), and AAV-EF1α-mt-EcGAPR-injected mice with 3.32E9 vg/eye (n = 8).

Data are presented as mean ± SEM. Statistical results: ns, not significant, *, *p* < 0.05; **, *p* < 0.01; ***, *p* < 0.001; *****p* < 0.0001.

Figure. S9.


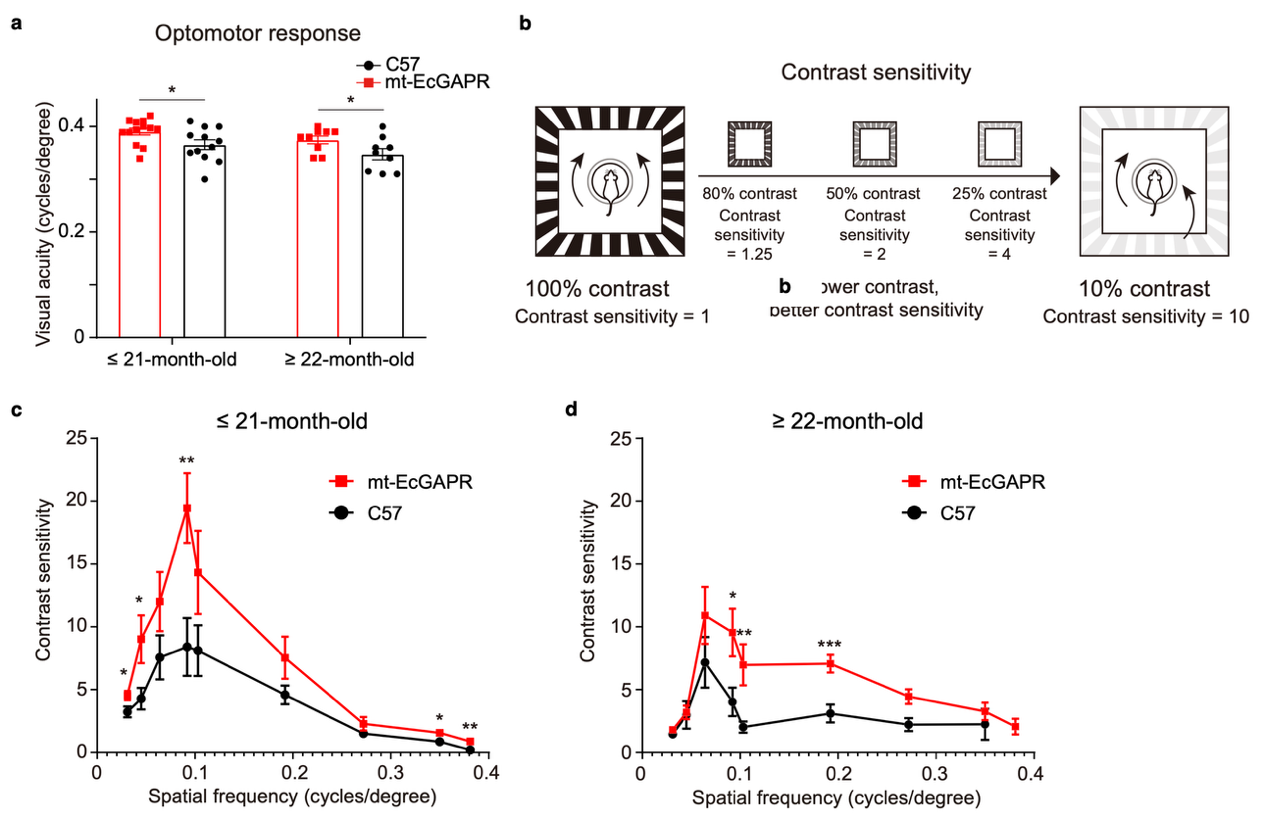


## Figure. S9. Enhanced visual function in mt-EcGAPR mice compared to age-matched C57 BL/6J controls, related to Figure 7

(a) Schematic of contrast sensitivity testing paradigm. Details of contrast sensitivity test is described in methods. In optomotor response test, 100% contrast is used.

(b) Visual acuity measured by optomotor response of C57 BL/6J mice and mt-EcGAPR mice up to 21-month-old or no less than 22-month-old. Black circles, C57 BL/6J mice. Red rectangles, mt-EcGAPR mice. N = 12, 9 for C57 BL/6J mice, n = 13, 9 for mt-EcGAPR mice.

(c) Contrast sensitivity of C57 BL/6J mice and mt-EcGAPR mice up to 21-month-old. Black circles, C57 BL/6J mice (n = 9). Red rectangles, mt-EcGAPR mice (n = 9).

(d) Contrast sensitivity of C57 BL/6J mice and mt-EcGAPR mice no less than 22-month-old. Black circles, C57 BL/6J mice (n = 8). Red rectangles, mt-EcGAPR mice (n = 9).

Data are presented as mean ± SEM. Statistical results: *, *p* < 0.05; **, *p* < 0.01; ***, *p* < 0.001; *t-test.*

Table S1.

| **Rhodopsin** | **Photocurrent or Pump activity** | | | **λ_max_**  **(nm)** | **Linear fitting** | | | **Mitochondrial Targeting** |
| --- | --- | --- | --- | --- | --- | --- | --- | --- |
|  | **HEK293** | **Neuron** | ***E.coli*** |  | **RP***  **(mV)** | **R**  **squared** | **95% CI** |  |
| BR | O | O | NA | 560 | -203 | 0.9947 | (-220.4, -188.7) | X |
| ArchT | O | O | O | 560 | -331 | 0.9875 | (-377.4, -294.9) | X |
| HtdR | O | O | O | 560 | -324 | 0.9545 | (-460.4, -250.1) | X |
| GR | O | O | O | 550 | -275 | 0.9903 | (-307.6, -248.2) | X |
| CsR | O | O | NA | 550 | -249 | 0.9904 | (-278.3, -225.2) | X |
| AXR | O | O | O | 540 | -257 | 0.9884 | (-297.4, -226.9) | X |
| Mac | O | O | NA | 540 | -258 | 0.9937 | (-288.7, -232.7) | ∆ |
| GPR | ∆ | ∆ | O | 530 | -348 | 0.9745 | (-439.8, -288.8) | O |
| APR | O | O | O | 530 | -214 | 0.9937 | (-236.9, -195.0) | ∆ |
| EcGAPR | O | O | O | 520 | -216 | 0.9842 | (-249.8, -190.3) | O |
| ‘O’ represents Yes; ‘X’ represents NA or No; ‘∆’ represents partial targeting. NA: Not available in this study.  ‘*’ RP represents reverse potentials resulted from linear fitting; 95% CI: 95% Confidence Intervals. | | | | | | | | |

## Table S1. Summary of properties for proton-pumping rhodopsins in the screening process.
