# Supplementary figures and images for: Ambient light alleviates retinal neurodegeneration in mice by powering mitochondria via the engineered optoenergetic rhodopsin
Source: Signal Transduct Target Ther. 2025 Oct 30;10:355. doi: 10.1038/s41392-025-02450-1 (PMC12572652; doi:10.1038/s41392-025-02450-1)

Fig.3d

IB: HSP60  
60 Kda

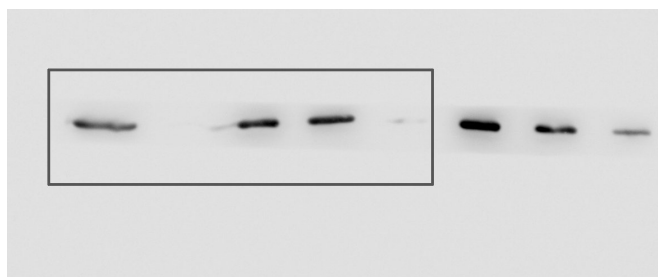

IB: EGFP  
55 KDa

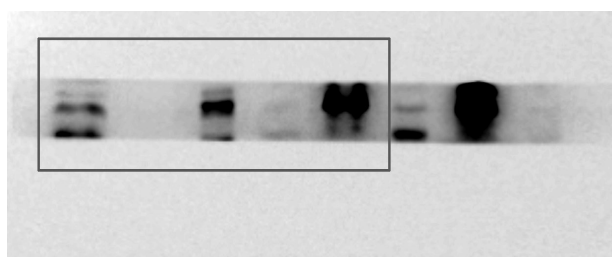

IB: TOM20  
20 KDa

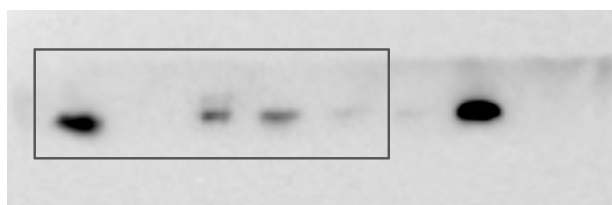

IB: mitofilin  
100 KDa

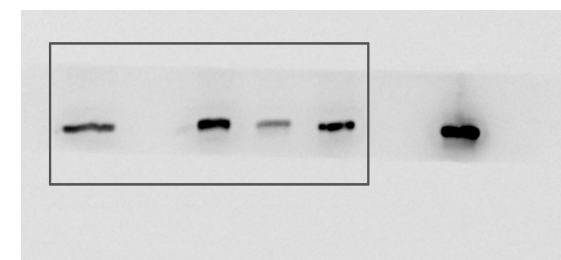

IB: GAPDH  
35 KDa

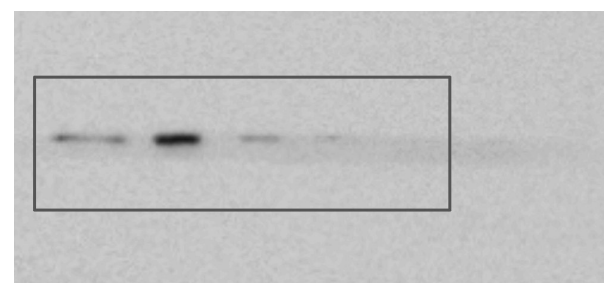

Fig. 5f

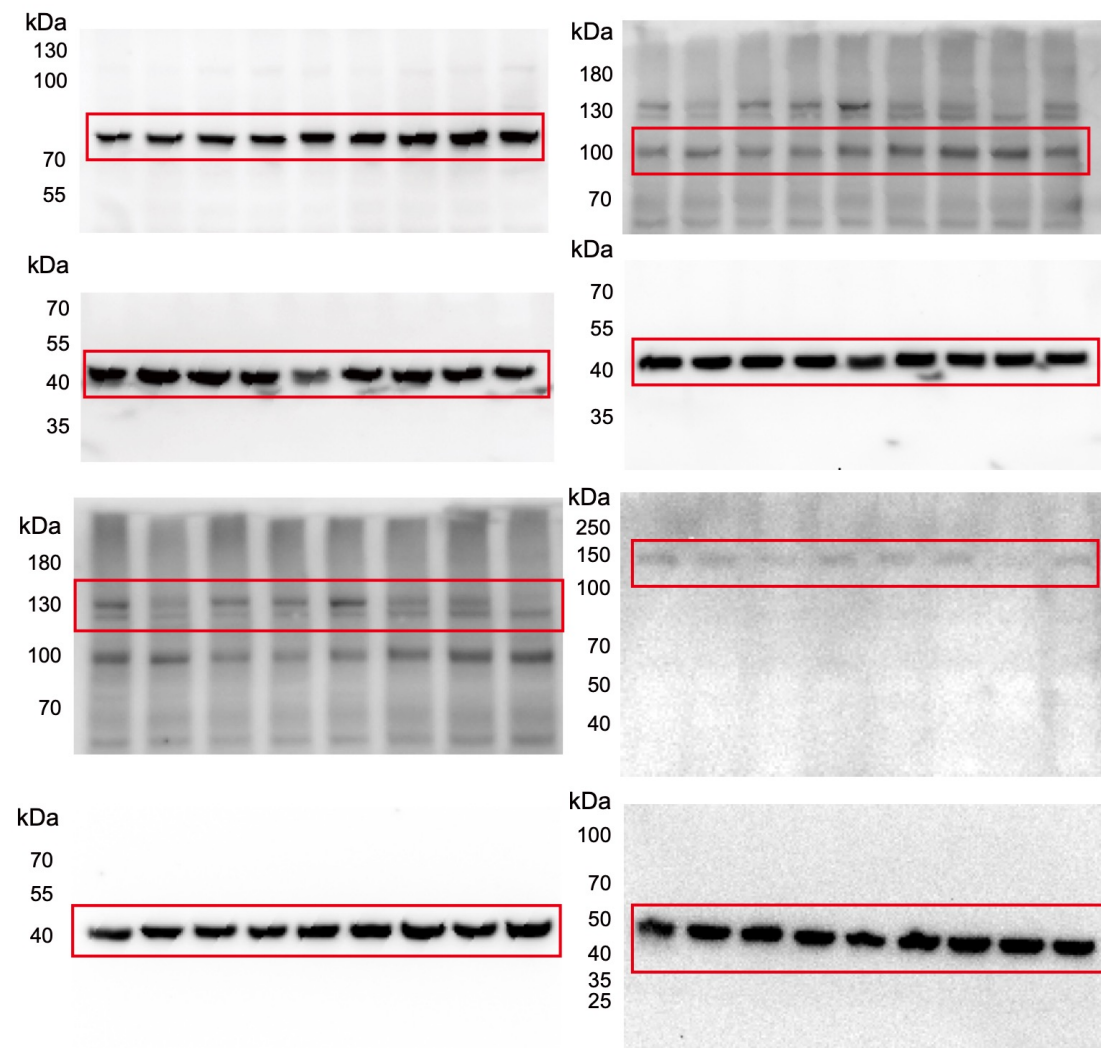

Fig. 5h

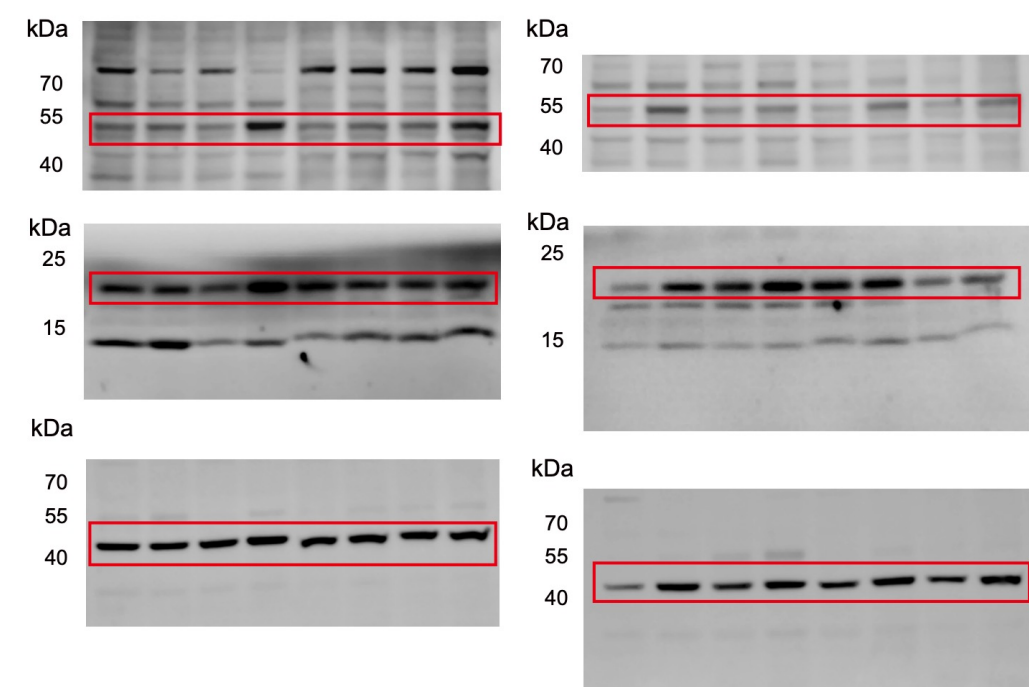

Supplementary Fig. 7h

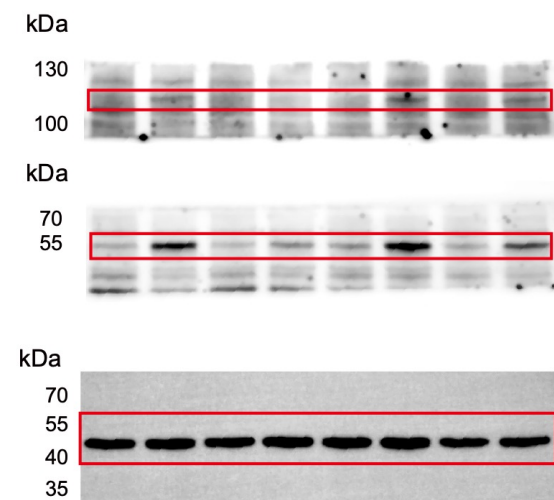

Supplement: Supplementary file 2 — Raw WB results [file 41392_2025_2450_MOESM2_ESM.pdf]
